# Kruppel-like factor 4-dependent Staufen1-mediated mRNA decay regulates cortical neurogenesis

**DOI:** 10.1038/s41467-017-02720-9

**Published:** 2018-01-26

**Authors:** Byoung-San Moon, Jinlun Bai, Mingyang Cai, Chunming Liu, Jiandang Shi, Wange Lu

**Affiliations:** 1 Department of Stem Cell Biology and Regenerative Medicine, Broad Center for Regenerative Medicine and Stem Cell Research, Keck School of Medicine, University of Southern California, Los Angeles, CA 90033 USA; 2 State Key Laboratory of Medicinal Chemical Biology and College of Life Sciences, Nankai University, 94 Weijin Road, 300071 Tianjin, China; 3 Department of Molecular and Cellular Biochemistry, University of Kentucky, Lexington, KY 40506 USA

## Abstract

Kruppel-like factor 4 (Klf4) is a zinc-finger-containing protein that plays a critical role in diverse cellular physiology. While most of these functions attribute to its role as a transcription factor, it is postulated that Klf4 may play a role other than transcriptional regulation. Here we demonstrate that Klf4 loss in neural progenitor cells (NPCs) leads to increased neurogenesis and reduced self-renewal in mice. In addition, Klf4 interacts with RNA-binding protein Staufen1 (Stau1) and RNA helicase Ddx5/17. They function together as a complex to maintain NPC self-renewal. We report that Klf4 promotes Stau1 recruitment to the 3′-untranslated region of neurogenesis-associated mRNAs, increasing Stau1-mediated mRNA decay (SMD) of these transcripts. Stau1 depletion abrogated SMD of target mRNAs and rescued neurogenesis defects in Klf4-overexpressing NPCs. Furthermore, Ddx5/17 knockdown significantly blocked Klf4-mediated mRNA degradation. Our results highlight a novel molecular mechanism underlying stability of neurogenesis-associated mRNAs controlled by the Klf4/Ddx5/17/Stau1 axis during mammalian corticogenesis.

## Abstract (web summary)

While being known as a transcription factor, Kruppel-like factor 4 (Klf4) may have other molecular functions. This study shows that Klf4 in neural progenitor cells regulate neurogenesis and self-renewal by interacting with RNA-binding protein Staufen1 and RNA helicase Ddx5/17 to control mRNA decay.

## Introduction

Neurogenesis is a complex process by which neurons and glial cells are generated from neural progenitor cells (NPCs). Depending on the stage of development, NPCs can either self-renew or differentiate to generate diverse types of neuronal and glial progeny^1^. This balance is finely controlled to ensure proper development of the nervous system and to maintain homeostasis in adult brain^2^. Not surprisingly, perturbation of this balance leads to various diseases, including cancer^3–5^. Although multiple signaling pathways influencing cell fate determination in NPCs have been investigated, including cell polarity, intra-cellular and inter-cellular signaling, transcription regulation, and epigenetic modification, questions remain, among which how post-transcriptional regulation of gene expression affects neurogenesis^6,7^.

Kruppel-like factor 4 (Klf4) is a zinc-finger-containing transcription factor that plays a critical role in various biological processes, including proliferation, differentiation, and apoptosis^8^. It was first characterized as a regulator of epithelial cell maturation in the skin^9,10^ and goblet cell differentiation in the colon^11^. Klf4 also regulates embryonic stem cell self-renewal^12,13^ and together with Oct4, Sox2, and c-Myc can reprogram somatic cells into induced pluripotent stem cells^14,15^. In the central nervous system, Klf4 expression inhibits axon regeneration in retinal ganglion cells by suppressing DNA-binding activity of phosphorylated signal transducer and activator of transcription 3^16,17^. Klf4 is also expressed in NPCs, where its developmental down-regulation is crucial for radial migration and maturation of newly born neurons^18^. Klf4 dysregulation is associated with hydrocephalus phenotypes seen in transgenic mice with Klf4 selectively overexpressed in NPCs^19^.

Staufen1 (Stau1) is a double-stranded (ds) RNA-binding protein functioning in post-translational mRNA regulation^20^. *Drosophila* Stau localizes *bicoid* and *oskar* mRNAs during oogenesis to form proper anteroposterior axis^21,22^. In the *Drosophila* developing nervous system, Stau is responsible for establishing asymmetry by localizing *prospero* mRNA into different daughter cells of the neuroblasts^23^. The mammalian homologs Stau1 and Stau2 contain several conserved dsRNA-binding domains and participate both in mRNA transport or localization activities and in mRNA decay^24–26^. In NPCs, asymmetric distribution of Stau2 and cargo mRNAs contributes to asymmetric cell division and subsequent neuronal differentiation^27,28^.

Stau1-mediated mRNA decay (SMD) is an mRNA degradation pathway that regulates biological processes as varied as myogenesis^29^, adipogenesis^30^, and cutaneous wound healing^31^. Unlike nonsense-mediated mRNA decay (NMD), SMD usually occurs following a normal translation termination event as a means to fine-tune the levels of transcripts harboring a Stau1 binding site (SBS)^20,32^. SBSs form either by intramolecular base pairing in the 3′-untranslated region (3′-UTR) of a target mRNA or by base pairing between a 3′-UTR *Alu* element in one mRNA and a partially complementary *Alu* element in a different mRNA or long noncoding RNA^31–34^. Stau1 recognizes SBSs located sufficiently downstream of a translation termination codon and recruits UPF1 to trigger mRNA decay^32^.

In this study, we show that neurogenesis-associated mRNAs in NPCs are degraded via the Stau1 pathway to maintain NPC identity, and this process is closely regulated by Klf4. Using immunohistochemistry and in vitro differentiation assays, we first show that Klf4 promotes NPC proliferation and inhibits differentiation in vivo and in vitro. Using mass-spectrometry (MS) and Western blot analysis, we then identified Stau1 and the RNA helicases Ddx5/17 as Klf4 interaction partners. We found that Stau1 recognizes specific neurogenesis-associated mRNAs and mediates their degradation. Through in vitro and in vivo photoactivatable ribonucleoside-enhanced crosslinking and immunoprecipitation (PAR-CLIP) and mRNA decay assays, we confirmed that mRNA degradation is controlled by binding of Stau1 with Klf4 and is dependent on Ddx5/17. Our results describe a new *Klf4* function in the nervous system and reveal a novel mechanism underlying mammalian neurogenesis.

## Results

### Klf4 deletion enhances neurogenesis in vivo and in vitro

**Fig. 1:**
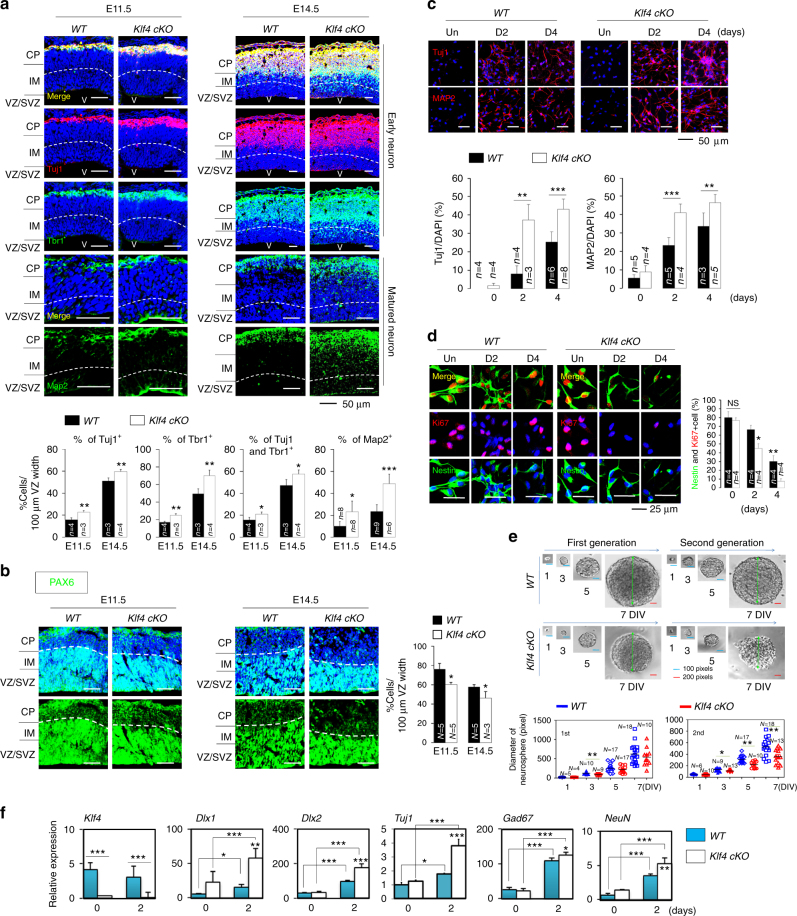
Klf4 down-regulation enhances neurogenesis in vivo and in vitro. **a** (Upper) Fixed coronal sections from E11.5 (left) or E14.5 (right) mouse forebrain stained with antibodies against Tuj1 (red), Tbr1 (green), or Map2 (green). Nuclear staining is shown by DAPI (blue). (Lower) Quantification of data shown above (E11.5: *Klf4* ^*fl/+*^ (*WT*), *n* = 4–8, *Klf4 conditional knockout* (*cKO*), *n* = 3–8, and E14.5: *Klf4*^*fl/+*^ (*WT*), *n* = 3–9, *Klf4 cKO*, *n* = 4–6). Scale bars, 50 μm. **b** (Left) Analysis of Pax6 in sections described in **a**. Scale bar, 50 μm. (Right) Quantification of data shown on the left (E11.5: *WT*, *n* = 5, *Klf4 cKO*, *n* = 5, and E14.5: *WT*, *n* = 5, *Klf4 cKO*, *n* = 3). **c** (Upper) Immunostaining with Tuj1 or Map2 (both red) antibodies in *WT* and *Klf4 cKO* NPCs in undifferentiation and differentiation conditions. Nuclear staining is shown by DAPI (blue). Scale bars, 50 μm. (Lower) Quantification of Tuj1-positive (*WT*-Un, *n* = 4, *WT*-D2, *n* = 4, *WT*-D4, *n* = 6, *Klf4 cKO*-Un, *n* = 4, *Klf4 cKO*-D2, *n* = 3, *Klf4 cKO*-D4, *n* = 8) and Map2-positive (*WT*-Un, *n* = 5, *WT*-D2, *n* = 5, *WT*-D4, *n* = 3, *Klf4 cKO*-Un, *n* = 4, *Klf4 cKO*-D2, *n* = 4, *Klf4 cKO*-D4, *n* = 5) cells in panels above. **d** Immunostaining with Ki67 (red) and Nestin (green) antibodies in *WT* and *Klf4 cKO* NPCs. DAPI (blue). Scale bars, 25 μm. (Right) Quantification of Ki67/Nestin double-positive cells in analysis shown at left (*n* = 4). **e** Single *WT* and *Klf4 cKO* NPCs were separated by serial dilution and neurosphere formation was induced for 7 days in vitro (DIV). Relative size of primary spheres grown to 7 DIV was quantified by Image J Software. Scale bars, blue (100 pixel), red (200 pixel). **f** qPCR analysis of indicated mRNAs in *WT* and *Klf4 cKO* NPCs. Values correspond to mean ± SD. ANOVA tests were performed to calculate significance (*P < 0.01, **P < 0.001, ***P < 0.0001). See also Supplementary Fig. 1a–f and Supplementary Table 4. Un undifferentiationn condition, D differentiation condition

To assess *Klf4* functions in neurogenesis, we generated a conditional knockout (cKO) mouse line by crossing *Nestin-Cre* with *Klf4*^*fl/fl*^ mice to delete *Klf4* in ventricular/subventricular zone NPCs (Supplementary Fig. 1a, b). The depletion of Klf4 gene expression is confirmed in NPCs at the (sub)ventricular zone of *klf4 cKO* (*Nes*^*cre*^;*Klf*^*fl/fl*^*)* mice in vivo and cultured NPCs derived from the mice in vitro using immunochemistry and quantitative PCR (qPCR) analysis (Supplementary Fig. 1c–f). Immunohistochemical analysis of forebrain regions of embryonic cortices derived from *wild-type* (*Klf4*^*fl/+*^) and *klf4 cKO* (*Nes*^*cre*^;*Klf*^*fl/fl*^) mice revealed an increase in the number of cells positive for Tuj1, an immature neuronal marker in *Klf4 cKO* versus *wild-type* controls (by ~7, ~9, and ~14% higher for Tuj1^+^ cells at E11.5, E14.5, and E18.5, respectively) (Fig. 1a; Supplementary Fig. 2a, b). We observed similar increases in post-mitotic deep-layer cortical neuron marker Tbr1 (by ~8, ~21, and ~6% higher for Tbr1^+^ cells at E11.5, E14.5, and E18.5, respectively) and in Tuj1 and Tbr1 double-positive cells (by ~5, ~10, and ~14% higher for Tuj1^+^ and Tbr1^+^ cells at E11.5, E14.5, and E18.5, respectively) (Fig. 1a; Supplementary Fig. 2a, b). The number of Map2-positive mature neurons or Dlx2-positive GABAergic neurons also significantly increased (Fig. 1a; Supplementary Fig. 2c, d and 3a, b). However, we observed slight differences in the number of cells positive for Tuj1, Tbr1, or Map2 in forebrain regions of embryonic cortices derived from postnatal day 2 (P2) (Supplementary Fig. 2a–d). To further examine neurogenesis defects in *Klf4 cKO*, we counted neurons in the six cortical layers that are born at different stages during corticogenesis, with deep-layer neurons preceding the birth of upper layer neurons. We performed immunostaining of triple markers to label different layers. No statistically significant differences in the number of Tbr1^+^/Ctip2^low^ layer 6 neurons that were born earlier in neurogenesis were observed between *WT* and *Klf4 cKO* (Supplementary Fig. 3c, d). However, the number of Ctip2-positive neurons at the layer 5 were significantly increased in Klf4 *cKO* brain (Supplementary Fig. 3c, d). The number of Satb2^+^/Ctip2^−^ neurons which label the upper layer 2 to 4 were also increased in the Klf4 *cKO* brain (by 62, 66, and 6.8% higher for Satb2^+^/Ctip2^−^ cells at E12.5, E18.5, and P2, respectively). However, the difference is significantly smaller at later stage of development compared with earlier stage. Overall, neurogenesis is increased in the Klf4 *cKO* brain. In *Klf4 cKO* mice, the number of Pax6-positive NPCs decreased significantly (by ~16 and ~11% lower for Pax6^+^ cells at E11.5 and E14.5, respectively) compared to *wild-type* mice (Fig. 1b). Moreover, in *Klf4 cKO* mice, the number of Ki67, Nestin, or Ki67 and Nestin double-positive NPCs decreased significantly (by ~8, ~5, and ~8% lower for Nestin^+^ cells, by ~0, ~6, and ~15% lower for Nestin^+^ cells, or by ~11, ~8, and ~4% lower for Ki67^+^/Nestin^+^ cells at E11.5, E14.5, and E18.5, respectively) compared with *wild-type* mice (Supplementary Fig. 4a, b). To rule out the possibility that the diminished number of progenitors during corticogenesis at *Klf4 cKO* mice is a result of increased cell death of progenitors, we stained E11.5, E14.5, E18.5, and P2 brain sections from the cortex with antibody against the activated form of cleaved caspase-3, a marker of apoptosis^35^. There was no significant difference in apoptosis between *wild-type* and *Klf4 cKO* mice (Supplementary Fig. 5a, b). These results demonstrate that *Klf4* loss in progenitors promotes an increase in neuron number and a concomitant decrease in NPC number. To assess direct effects of *Klf4* loss on NPC differentiation capacity, we cultured *wild-type* and *Klf4 cKO* NPCs derived from E11.5 mouse brain in the presence of basic fibroblast growth factor (bFGF) and then withdrew bFGF to induce neural differentiation. Consistent with our in vivo results, over the course of differentiation, the number of Tuj1-positive and Map2-positive cells increased, while the number of Ki67-positive and BrdU-positive, or Nestin/Ki67 double-positive cells slightly decreased in *Klf4 cKO* NPCs cultures relative to *wild-type* controls (Fig. 1c, d; Supplementary Fig. 6a–c). We also assessed *Klf4* function in NPC self-renewal activity using a single-cell clonal neural sphere formation assay. Spheres containing *Klf4 cKO* NPCs had significantly smaller diameters than those comprised of *wild-type* NPCs at 3 days of culture in vitro (DIV) during the first generation (*P* = 0.0013), and at 3, 5, and 7 DIV during the second generation (*P* = 0.024, 3DIV; *P* = 0.00087, 5 DIV; *P* = 0.0034, 7 DIV) (Fig. 1e), suggestive of a role for Klf4 in NPC self-renewal. Moreover, to determine the number of cells that exited the cell cycle in a 6-h period, we quantified the proportion of Ki67^+^, BrdU^+^, and Ki67^+^ or Ki67^−^ cells in the total BrdU^+^ cell population. Our results indicated that the self-renewing progenitor populations were increasingly depleted, as ~7 or ~11% more progenitors exited the cell cycle in *Klf4 cKO* NPCs compared with *wild-type* NPCs in P1 (passage 1) or P10 status (Supplementary Fig. 6d–g). Further analysis using reverse transcriptase qPCR (RT-qPCR) showed that *Klf4 cKO* NPCs exhibited increased expression of the neuronal markers *Dlx1*, *Dlx2*, *Tuj1*, *Gad67*, and *NeuN* (Fig. 1f). Taken together, these data suggest that a decreasing progenitor pool in *Klf4 cKO* mice available to generate the deep and low layer cortical neurons.

### Klf4 regulates neuronal differentiation of NPCs

**Fig. 2:**
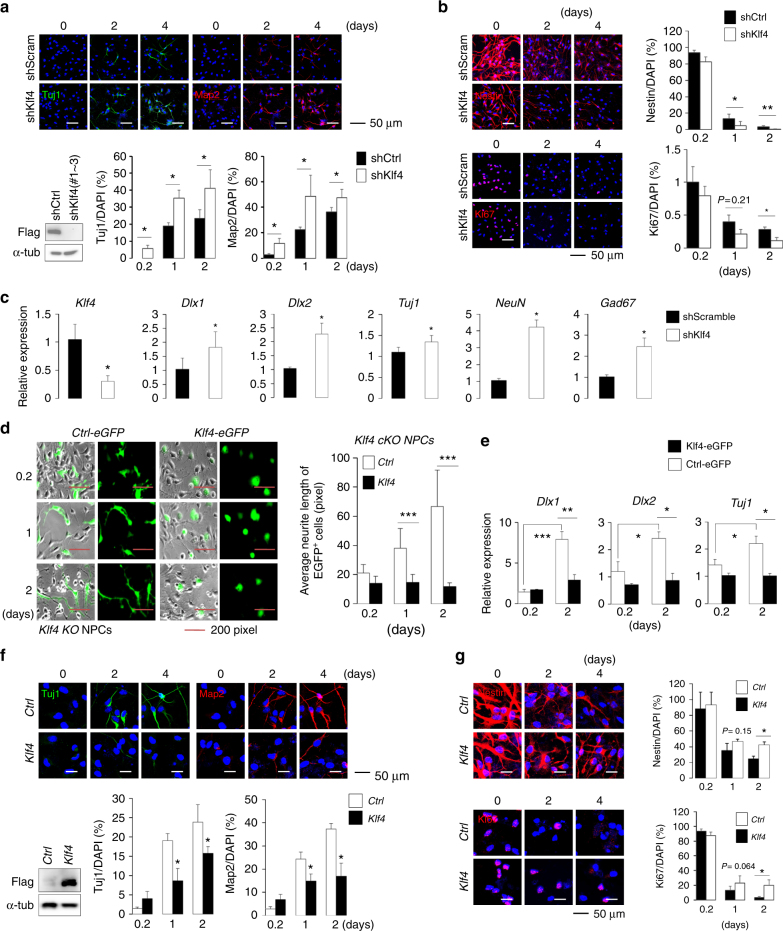
Klf4 regulates NPC neuronal differentiation and proliferation. **a** (Upper) Immunostaining with Tuj1 (red) or Map2 (red) antibodies in NPCs infected with pLKO.1-shScramble or pLKO.1-shKlf4 (#1, #2, and #3) lentivirus. DAPI (blue). Scale bars, 50 μm. (Lower left) NPCs expressing Flag-Klf4 were transfected with pLKO.1-shScramble or pLKO.1-shKlf4 #1, #2, and #3, and one day later, Flag and α-tubulin in lysates were detected by immunoblotting (*n* = 2). (Lower right) Quantification of the proportion of Tuj1^+^ or MAP2^+^ cells in the analysis shown above. **b** (Left) Immunostaining of samples equivalent to those shown in **a** with Nestin or Ki67 (both red) antibodies. DAPI (blue). Scale bars, 50 μm. (Right) Quantification of Ki67^+^ (*n* = 2 or 3) or Nestin^+^ (*n* = 3) cells in samples analyzed at left. **c** qPCR analysis of indicated transcripts in NPCs transfected with pLKO.1-shScramble or pLKO.1-shKlf4 lentiviral vector and grown 2 days in N2 medium without bFGF (*n* = 3). **d** (Left) Klf4 *cKO* NPCs transfected with Control-EGFP vector or Klf4-EGFP vector. GFP^+^ cells were assessed after 1 or 2 days of culture in N2 medium. (Right) Analysis of neurite length in GFP-positive NPCs on 1 or 2 days of differentiation. **e** qPCR analysis of indicated transcripts in samples equivalent to those shown in **d** (*n* = 3). **f** (Upper) Immunostaining with Tuj1 (green) or Map2 (red) antibodies in NPCs infected pCDH-Klf4 or pCDH-control lentivirus. DAPI (blue). Scale bars, 50 μm. (Lower left) NPCs were infected with pCDH-Flag-Klf4 or pCDH-control lentivirus, and one day later, Flag and α-tubulin in lysates were detected by immunoblotting (*n* = 2). (Lower) Quantification of results shown above. **g** (Left) Immunostaining with Nestin or Ki67 (both red) antibodies in samples shown in **f**. DAPI (blue). Scale bars, 50 μm. (Right) Quantification of results shown at left. Data are presented as mean ± SD. *t* test analysis was performed to calculate statistical significance (*P < 0.05, **P < 0.005, ***P < 0.0005). See also Supplementary Fig. 7a–d and 16 (upper panel)

To further investigate whether Klf4 regulates neurogenesis, we knocked down endogenous *Klf4* in cultured *wild-type* NPCs using shKlf4 lentivirus and then induced differentiation for 2 or 4 days. Under undifferentiated, 2-day or 4-day differentiated conditions, we observed a significant increase in Tuj1^+^ cells and Map2^+^ cells, a robust decrease in Nestin^+^ cells, and a slight decrease in Ki67^+^ cells in knockdown versus control cells (Fig. 2a, b). Further RT-qPCR analysis showed that cultures of *Klf4* knockdown NPCs exhibited increased *Dlx1*, *Dlx2*, *Tuj1*, *Gad67*, and *NeuN* expression relative to controls (Fig. 2c). Several Klf4 short hairpin RNAs (shRNAs) were able to inhibit Klf4 expression (Supplementary Fig. 7a; Supplementary Table 1), and comparable phenotypes were observed using a different Klf4 shRNA (data not shown).

We also used a rescue experiment to assess *Klf4* function in neurogenesis. To do so, we transfected *Klf4 cKO* NPCs with vectors expressing *EGFP-Klf4* or *EGFP-Ctrl* to assess potential rescue effects. Indeed, compared to *Klf4 cKO* NPCs, transfected cells showed more rounded morphology and exhibited significantly shorter neurites (62% shorter under the day-1 differentiation condition, *P* < 0.0001; 82% shorter under the day 2 differentiation condition, *P* < 0.0001) (Fig. 2d and Supplementary Fig. 7b), reflecting a less differentiated state. We then undertook RT-qPCR analysis using *wild-type* NPCs transfected with the same vectors. NPCs overexpressing *Klf4* (Klf4-NPCs) showed significantly decreased neuronal markers *Dlx1*, *Dlx2*, *Tuj1*, *NeuN*, *Pitx3*, *DAT*, *Lmx1a*, *Foxa2*, *En1*, and* Nurr1* expression and a slight increase in *Pax6* expression under the day 2 differentiation condition, suggesting inhibition of neurogenesis (Fig. 2e and Supplementary Fig. 7c, d). Immunocytochemistry confirmed these results and revealed a substantial decrease in percentages of cells positive for Map2 (D2, *P* = 0.048; D4, *P* = 0.0043) and Tuj1 (D2, *P* = 0.033; D4, *P* = 0.043) on day 2 and day 4 of differentiation (Fig. 2f). We also observed a slight increase in percentages of Nestin^+^ cells (D4, *P* = 0.02) and Ki67^+^ cells (D4, *P* = 0.017), supporting the idea that Klf4 promotes NPC self-renewal (Fig. 2g).

### Klf4 interacts with Stau1 during cortical neurogenesis

**Fig. 3:**
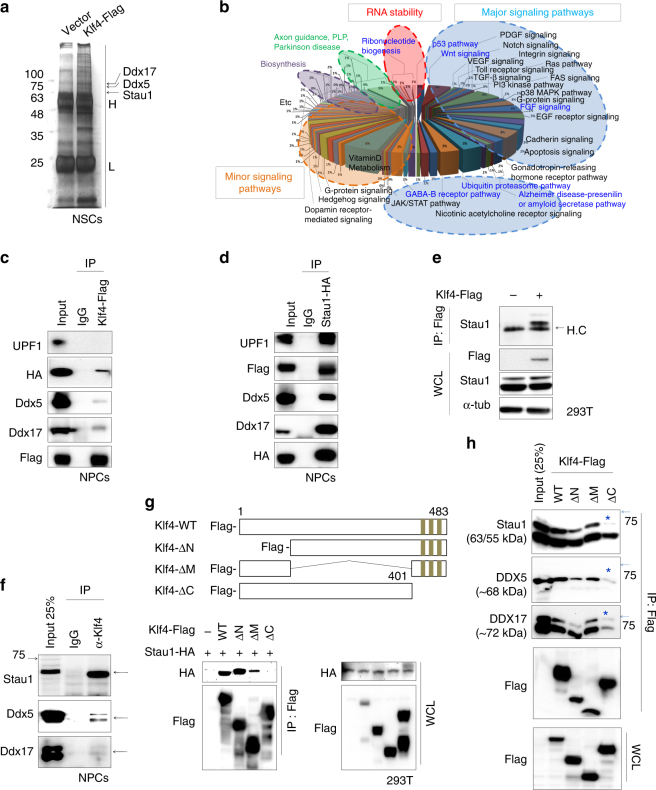
Stau1 interacts with Klf4 in NPCs. **a** Anti-flag immunoprecipitates from Flag-Klf4-NPCs were separated by SDS-PAGE and visualized by silver staining. Specific bands were cut and analyzed by mass spectrometry. **b** GO analysis of the Klf4 interactome in NPCs. Several enriched Klf4 interaction partners are associated with post-transcriptional RNA regulation, among them are the RNA-binding protein Stau1 and RNA helicases of the DDXs protein family. **c** Co-immunoprecipiation of HA-Stau1 or endogenous UPF1, Ddx5, and Ddx17 in NPCs infected with Flag-Klf4 and HA-Stau1 lentivirus using anti-IgG (negative control), or anti-Flag antibodies (*n* = 2). **d** Co-IP of Flag-Klf4 or endogenous UPF1, Ddx5, and Ddx17 in NPCs infected with Flag-Klf4 and HA-Stau1 lentivirus using anti-IgG (negative control), or anti-HA antibodies (*n* = 2). **e** Co-IP of endogenous Stau1 in HEK293T cells transfected with Flag-tagged Klf4. **f** Co-IP of endogenous Stau1 and Klf4 in undifferentiated NPCs using anti-IgG (negative control) and anti-Klf4. **g** Domain mapping of Klf4 interaction with Stau1. (Upper) Schematic showing domains deleted from wild-type Klf4 protein. (Lower) Co-IP of Klf4 mutants with HA-tagged Stau1 in cells expressing full-length HA-tagged Stau1 and Flag-tagged Klf4 mutants. IP was performed using anti-Flag antibody-conjugated agarose beads and immunoblotting with anti-HA and anti-Flag antibodies. **h** Co-IP of endogenous Stau1, Ddx5, and Ddx17 in NPCs transfected with Klf4 mutants and/or Stau1. Blue asterisks indicate position of each protein. See also Supplementary Fig. 7e–h and 16–18. H IgG heavy chain, L IgG light chain, WCL whole cell lysate

To define the molecular mechanisms underlying Klf4 regulating NPC differentiation, we searched for proteins interacting with Klf4 protein through MS analysis. Due to a lack of ChIP-grade antibodies, we expressed Flag-tagged Klf4 in *Klf4 cKO* NPCs, in which Flag-tagged Klf4 expression is similar to endogenous Klf4, and carried out immunoprecipitation (IP) with anti-Flag and control anti-IgG antibodies in parallel samples (Fig. 3a). Gene ontology (GO) analysis of MS results showed that a significant portion functioned in regulation of RNA stability (Fig. 3b). Among Klf4 interacting proteins, we focused on Stau1, Ddx5, and Ddx17, which showed an average enrichment of 2.99-, 2.81-, and 2.81-fold, respectively (Supplementary Fig. 7e). Stau1 is a dsRNA-binding protein functioning in post-transcriptional gene regulation with other factors such as UPF1^32,34,36^. Ddx5 and its paralog Ddx17 are DEAD box RNA helicases functioning in RNA splicing and post-transcriptional regulation^37,38^.

To confirm Klf4–Stau1 interaction, we overexpressed Flag-tagged Klf4 and HA-tagged Stau1 in NPCs or HEK293T cells, and performed co-immunoprecipiation (co-IP) with anti-Flag or anti-HA antibodies. As shown in Fig. 3c, d, Klf4 co-immunoprecipitated with Stau1, and inversely, Stau1 also co-immunoprecipitated with Klf4, while no signals were detected in IgG control or cells transfected with a negative control vector (Fig. 3c, d; Supplementary Fig. 7f, g). Moreover, endogenous Stau1 co-immunoprecipitated with overexpressed Flag-tagged Klf4 in HEK293T cells (Fig. 3e). Interaction of endogenous proteins was also confirmed by co-IP of endogenous Klf4 and Stau1 proteins in NPC lysates (Fig. 3f). Furthermore, our co-IP data also confirmed interaction of Klf4 or Stau1 with Ddx5/17 (Fig. 3c, d, f). Interestingly, we also found that Stau1 protein, but not Klf4, co-immunoprecipitated with endogenous UPF1 (Fig. 3c, d).

To map Klf4 domain(s) required for interaction with Stau1, we generated various Klf4 deletion mutants and assessed their interactions with Stau1 by co-IP in HEK293T cells (Fig. 3g). Based on this analysis, a Klf4 C-terminal deletion mutant (ΔC) that lacks the three zinc-finger domains did not interact with Stau1, while all other mutants tested showed binding comparable to the wild-type protein (Fig. 3g). In addition, Klf4 C-terminal deletion mutant (ΔC) did not interact with Ddx5 or Ddx17 (Fig. 3h). Because Stau1 protein is dsRNA-binding protein and is known to bind to its target mRNAs for SMD^32,34^, it is important to determine whether Klf4–Stau1 interaction is mediated by RNAs. To address this, we treated cell lysates with RNase A and then performed co-IP experiment. Interestingly, we found that Klf4–Stau1 interaction is independent of RNAs (Supplementary Fig. 7h). Together, these findings suggest that in NPCs, Klf4 interacts with Stau1, Ddx5, and Ddx17, all of which are known to function in post-transcriptional RNA regulation, and that the Klf4 zinc-finger domain is required for the interactions.

### Inhibition of neurogenesis by Stau1 is Klf4-dependent

**Fig. 4:**
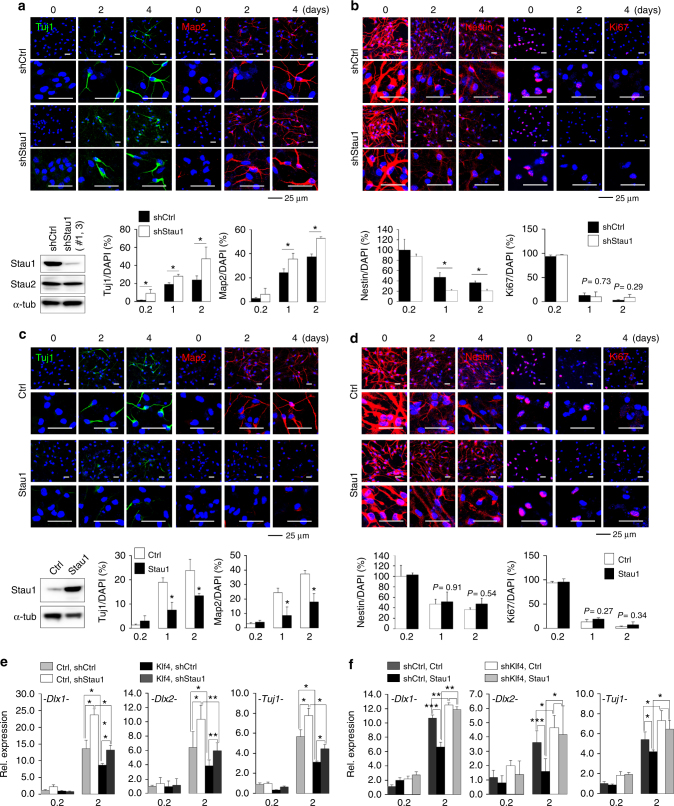
Stau1 inhibits neurogenesis in a Klf4-dependent manner. **a** (Upper) Immunostaining with Tuj1 (green) or Map2 (red) antibodies in NPCs infected with pLKO.1-shScramble or pLKO.1-shStau1 (#1 and #3) lentivirus. DAPI (blue). Scale bars, 25 μm. (Lower left) Stau1, Stau2, and α-tubulin in samples analyzed in **a** were were detected by immunoblotting (*n* = 2). See also Supplementary Fig. 8a. (Lower right) Corresponding quantification of number of Tuj1^+^ and Map2^+^ cells. **b** (Upper) Immunostaining with Nestin or Ki67 (both red) antibodies of samples analyzed in **a**. DAPI (blue). Scale bars, 50 μm. (Lower) Corresponding quantification of Nestin^+^ (*n* = 3) and Ki67^+^ (*n* = 2 or 3) cells. **c** (Upper) Immunostaining of NPCs infected with pCDH-control or pCDH-Stau1 lentivirus with Tuj1 (green) or MAP2 (red) antibodies. DAPI (blue). Scale bars, 25 μm. (Lower left) Stau1 and α-tubulin in samples analyzed in **c** were detected by immunoblotting (*n* = 2). (Lower) Corresponding quantification of data shown above. **d** (Upper) Immunostaining of samples equivalent to those shown in **c** with Nestin or Ki67 (both red) antibodies. DAPI (blue). Scale bars, 25 μm. (Lower) Corresponding quantification of Nestin^+^ (*n* = 3) and Ki67^+^ (Ctrl, *n* = 3; Stau1, *n* = 2) cells. **e** RT-qPCR of indicated transcripts in control- or Klf4-overexpressing NPCs infected with pLKO.1-shScramble or pLKO.1-shStau1 lentivirus cultured for 0.2 or 2 days in differentiation conditions (*n* = 3). **f** RT-qPCR of indicated transcripts in control- or Stau1-overexpressing NPCs infected with pLKO.1-shScramble or pLKO.1-shKlf4 lentivirus cultured for 0.2 or 2 days in differentiation conditions (*n* = 3). Data are shown as mean ± SD. ANOVA tests were performed to calculate statistical significance (*P < 0.05, **P < 0.005, ***P < 0.0005). See Supplementary Table 4 and Supplementary Fig. 18

Given that Stau1 interacts with Klf4 in NPCs, we asked what role Stau1 may play during neurogenesis. Stau1-deficient mice have been shown to have defects in neuronal morphogenesis^39^. Stau1 shRNA specifically knockdown endogenous Stau1 but not Stau2 in cultured NPCs (Supplementary Fig. 8a). Knockdown of endogenous Stau1 showed a significant increase in percentages of Tuj1^+^ cells and Map2^+^ cells at 2 or 4 days of differentiation (Tuj1, D2, *P* = 0.011; Tuj1, D4, *P* = 0.048; Map2, D2, *P* = 0.007; Map2, D4, *P* = 0.022) (Fig. 4a). A significant decrease in the percentage of Nestin^+^ cells at days 2 (*P* = 0.034) and 4 (*P* = 0.04) of differentiation was also observed (Fig. 4b). However, Stau1 knockdown did not significantly alter cell proliferation, as determined by the number of Ki67^+^ cells (Fig. 4b). We also tested whether Stau1 overexpression in NPCs would affect neurogenesis. Compared to control cells, NPCs infected with Stau1 lentivirus showed significantly decreased Tuj1 (D2, *P* = 0.02; D4, *P* = 0.0055) and Map2 (D2, *P* = 0.014; D4, *P* = 0.0028) staining under differentiation conditions (Fig. 4c). We also observed a slight but not statistically significant increase in percentages of Nestin^+^ cells in Stau1-overexpressing cells (Fig. 4d). Similarly, there was no change in the proliferation marker Ki67 between Stau1-overexpressing and control cells (Fig. 4d). We next assessed biological role of Stau1 in neurogenesis using an in utero electroporation system. Electroporated embryos with shStau1 lentiviral vectors were readily identifiable by EGFP expression (Supplementary Fig. 8b). The number of Tuj1^+^ cells significantly increased in the VZ/SVZ/IZ region in Stau1 knockdown GFP-positive cells relative to control GFP-positive cells (Supplementary Fig. 8b, c). Conversely, when embryos were electroporated with Stau1-mCherry vector, the number of Tuj1^+^ cells significantly decreased in the VZ/SVZ/IZ region in Stau1-overexpressing mCherry-positive cells relative to control-mCherry-positive cells (Supplementary Fig. 8d, e). These results suggested that neuronal differentiation is enhanced by Stau1 knockdown and inhibited by Stau1 overexpression. To determine whether this effect was Klf4-dependent, we tested the effect of *Stau1* loss by shStau1 lentiviral infection in control or Klf4-overexpressing NPCs cultured in undifferentiation or differentiation conditions. This analysis showed that enhanced neurogenesis seen following *Stau1* loss is significantly abolished in *Klf4*-overexpressing NPCs (Fig. 4e; Supplementary Fig. 9a, b). In other words, neurogenesis defects seen in Klf4-overexpressing NPCs were significantly reversed by *Stau1* loss. These findings were further confirmed by in utero electroporation experiments. As similar to in vitro data, the number of Tuj1^+^ cells significantly decreased in the VZ/SVZ/IZ region in shscramble-GFP and Klf4-mCherry co-expressing cells relative to shscramble-GFP and control-mCherry co-expressing cells. Additionally, reduced Tuj1^+^ cells shown in the shscramble-GFP and Klf4-mCherry co-expressing cells were significantly rescued in the VZ/SVZ/IZ region in shStau1-GFP and Klf4-mCherry co-expressing cells (Supplementary Fig. 9c). These findings suggest that the Stau1 protein in neurogenesis is downstream of Klf4 in regulating neuronal differentiation. Moreover, we also undertook qPCR to assess the effect of *Stau1* overexpression in control or *shKlf4* knockdown NPCs cultured in undifferentiation or differentiation conditions. Interestingly, Stau1 overexpression significantly reduced *Dlx1*, *Dlx2*, and *Tuj1* mRNA expression in control NPCs but not in the *shKlf4* knockdown NPCs (Fig. 4f; Supplementary Fig. 9d). These results suggest that Stau1 regulates neurogenesis in a Klf4-dependent manner.

### Stau1 promotes degradation of neurogenesis-associated mRNAs

**Fig. 5:**
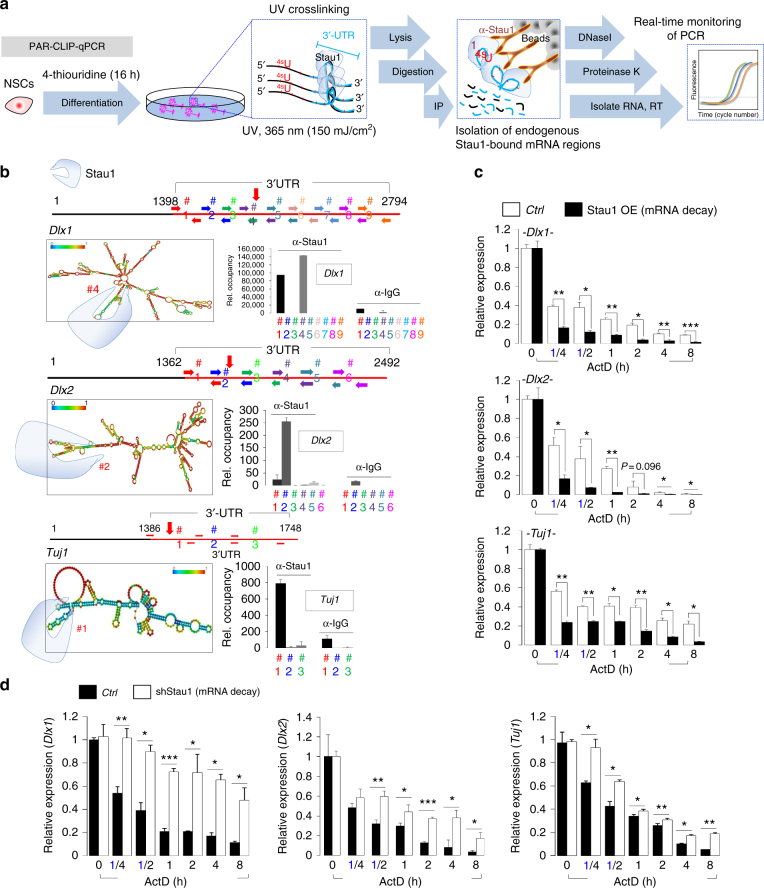
Stau1 binds to neurogenesis-associated mRNAs and promotes their degradation. **a** Schematic showing use of PAR-CLIP-qPCR to detect Stau1 target mRNAs and binding sites. **b** (Left) RNA secondary structure prediction using online software (http://rna.tbi.univie.ac.at/) of hairpin formation in 3′-UTR sequences of *Dlx1*, *Dlx2*, and *Tuj1*. Potential Stau1 binding regions are marked with vertical red arrows. (Right) PAR-CLIP qPCR shows enrichment of Stau1 binding at various locations on indicated mRNAs. *X*-axis, primer sets; *y*-axis, relative fold change. **c**, **d** NPCs stably overexpressing (OE) Stau1 or control vectors (**c**) or infected with shScramble or shStau1 lentiviral vectors (**d**) were treated with actinomycin D (5 μg/ml) for indicated times and mRNAs were prepared for RNA stability assays (*n* = 3). Results were normalized to control NPCs at 0 h. Data are presented as mean ± SD. *t* tests were conducted to calculate statistical significance (*P < 0.05, **P < 0.005, ***P < 0.0005). See also Supplementary Fig. 9a, d and 10a–c

Recent studies show that Stau1 functions as a post-transcriptional regulator by first recognizing specific secondary structure or sequences of the 3′-UTR of target mRNAs^40,41^. Therefore, to determine how neurogenesis-associated mRNAs are specifically recognized by Stau1, we initially analyzed Stau1 RIP or CLIP sequencing profiles performed in HEK293T cells. HEK293T cells were chosen based on the evidence suggesting a possible neural lineage origin of HEK293T cells, which explains the expression of neuro-specific genes in these cells^42,43^. This is further supported by an online database (GSE52447,www.ncbi.nlm.nih.gov/geo/query/acc.cgi?acc=GSE52447). Interestingly, we found that numerous mRNAs recognized by Stau1 function in neurogenesis (12.8%) (Supplementary Fig. 10a, b). Indeed, Stau1 protein bindings were highly enriched in 3′-UTRs of *Dlx1*, *Dlx2*, and *Tuj1* mRNAs (Supplementary Fig. 10c), suggesting that Stau1 might play a role in neurogenesis through degradation of these mRNAs. Stau1 protein is highly conserved with 95% identity in humans and mice. Nevertheless, to confirm whether Stau1 specifically binds *Dlx1*, *Dlx2*, and *Tuj1* mRNAs in mouse NPCs, we performed PAR-CLIP qPCR analysis in mouse NPCs using an anti-Stau1 antibody (Fig. 5a). We found that each potential target mRNA contained at least one region recognized and bound by Stau1 (Fig. 5b). The predicted secondary structures of these mRNAs confirmed the presence of a double-stranded loop at binding regions (Fig. 5b). To examine potential effects on decay of *Dlx1*, *Dlx2*, and *Tubb3* mRNAs, we carried out an RNA stability assay, in which we treated cells with actinomycin D, a transcriptional inhibitor, and then assess target mRNA levels at various time points by RT-qPCR. Compared to control NPCs, Stau1-overexpressing NPCs (Stau1-NPCs) showed significantly more rapid degradation of *Dlx1*, *Dlx2*, and *Tuj1* mRNAs (Fig. 5c). Conversely, Stau1 knockdown significantly inhibited decay of target mRNAs, suggesting that Stau1 regulates stability of these neurogenesis-associated mRNAs (Fig. 5d).

### Klf4 regulates SMD during neurogenesis

**Fig. 6:**
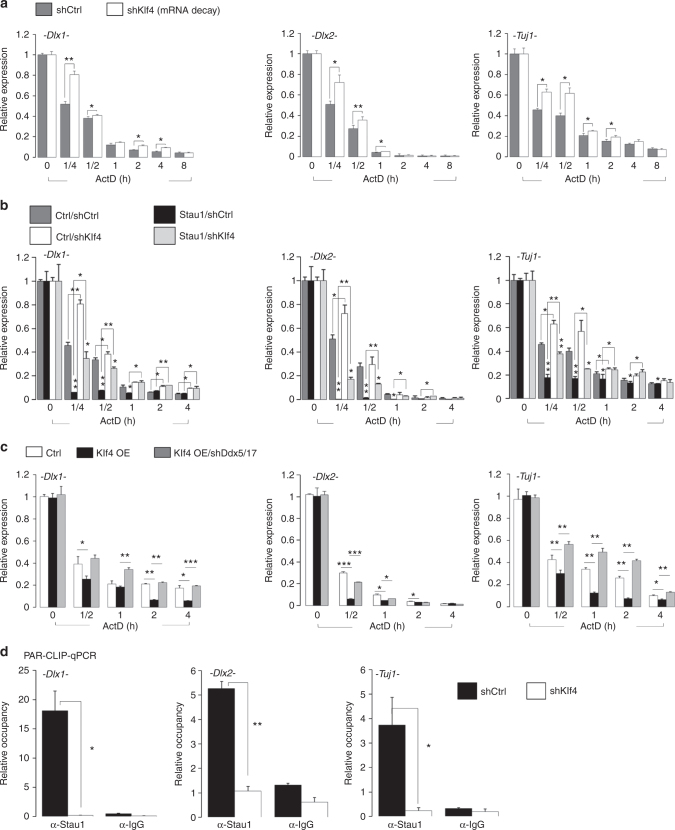
Klf4 knockdown decreases Stau1 binding to target mRNAs and slows the rate of SMD. **a** NPCs infected with shScramble or shKlf4 lentivirus were treated with actinomycin D (5 μg/ml) for indicated times and mRNAs were prepared for RNA stability assays (*n* = 3). **b** RT-qPCR to assess the mRNA decay following Stau1 overexpression in NPCs transduced with shScramble or shKlf4 knockdown constructs. NPCs were treated with actinomycin D (5 μg/ml) for indicated times, and mRNAs were prepared for RNA stability assay (*n* = 3). **c** RT-qPCR to assess the mRNA decay at various time points following Ddx5/17 loss in NPCs overexpressing (OE) Klf4 (*n* = 3). **d** PAR-CLIP qPCR to assess Stau1 enrichment on target mRNAs in NPCs transduced with shKlf4 knockdown or shScramble constructs. *Dlx1* (#6), *Dlx2* (#6), and *Tuj1* (#2) primers were used, and α-IgG was used as a negative control. Data are presented as mean ± SD. *t* tests were performed to calculate statistical significance (*P < 0.05, **P < 0.005, ***P < 0.0005). See also Supplementary Fig. 11a–d, 12c, and Supplementary Table 4

To assess the role of Klf4 in mRNA decay during neuronal differentiation, we carried out an RNA stability assay using NPCs infected with either control shscramble or shKlf4 lentivirus, and *wild-type* or *Klf4 cKO* NPCs. Degradation rates of *Dlx1*, *Dlx2*, and *Tuj1* mRNAs were all significantly lower in both Klf4 KO and knockdown NPCs (Fig. 6a; Supplementary Fig. 11a). To assess *Klf4* function in SMD during the course of neuronal differentiation, we compared SMD in NPCs overexpressing Stau1 and infected with either shscramble or shKlf4 lentivirus. Stau1 significantly promoted decay of neurogenesis-associated mRNAs in NPCs infected with control shRNA lentivirus at 15 min, 30 min, and 1 h, respectively) (Fig. 6b). However, enhanced mRNA decay by Stau1 overexpression was critically decreased in NPCs infected with shKlf4 lentivirus (Fig. 6b). These results demonstrated that Klf4 is required for Stau1-mediated SMD of *Dlx1*, *Dlx2*, and *Tuj1* mRNA.

SMD is reportedly triggered when Stau1 recruits UPF1, which has dsRNA helicase activity, to target mRNAs^32,44^. MS analysis and co-IP experiment identified RNA helicases Ddx5/17 instead of UPF1 as Klf4 interacting partners (Fig. 3c). Ddx5 and its paralog Ddx17 form heterodimers and they are involved in the regulation of target mRNA stability through cooperating with NMD factor, UPFs protein^45^. Therefore, we asked whether Ddx5/17 would modulate changes in mRNAs stability seen in Klf4-NPCs. To address this, we monitored endogenous target mRNA levels in wild-type and in Klf4-overexpressing NPCs infected with *shscramble* or *shDdx5/17* lentivirus. Interestingly, Klf4 overexpression significantly enhanced the decay of *Dlx1*, *Dlx2*, and *Tuj1* mRNAs, while Ddx5/17 knockdown blocked this effect (Fig. 6c). Because the increased decay of target neurogenesis-associated mRNAs is related to the neurogenesis defect seen in Klf4-NPCs, we assessed whether Ddx5/17 loss can rescue defect of neurogenesis following Klf4 overexpression in NPCs. As expected, our qPCR data revealed that knockdown of Ddx5/17 in Klf4-NPCs significantly rescued the neurogenesis defect seen in Klf4-NPCs, and these were further confirmed by Western blot analysis (Supplementary Fig. 11b–d). Moreover, Ddx5/17 interacts with Stau1 protein (Fig. 3d), and their interaction was increased by Klf4 overexpression (Supplementary Fig. 11e). The mRNA decay through Klf4-Ddx5/17 might be driven through the Stau1-mediated decay machinery. To test whether Ddx5/17 modulates Stau1’s role in the decay of target mRNAs, we carried out a RNA stability assay using *wild-type* NPCs and Stau1-NPCs infected with *shscramble* or *shDdx5/17* lentivirus. Stau1 overexpression significantly increased the decay of *Dlx1*, *Dlx2*, and *Tuj1* mRNAs, while Ddx5/17 knockdown delayed this effect (Supplementary Fig. 12a). Moreover, our qPCR data showed that Ddx5/17 knockdown also rescued defect of neurogenesis seen in Stau1-NPCs (Supplementary Fig. 12b). To further investigate Klf4’s relevance to SMD, we performed PAR-CLIP qPCR analysis using anti-Stau1 antibody in NPCs infected with control or shKlf4 lentivirus. Interestingly, in Klf4 knockdown cells, less *Dlx1*, *Dlx2*, and *Tuj1* mRNAs were bound by Stau1 protein relative to the controls (Fig. 6d; Supplementary Fig. 12c). Overall, these experiments demonstrate that activities of Klf4, Ddx5/17, and Stau1 govern neuronal differentiation by regulating stability of neurogenesis-associated mRNAs.

### Klf4 promotes Stau1 enrichment on neuronal target mRNAs

**Fig. 7:**
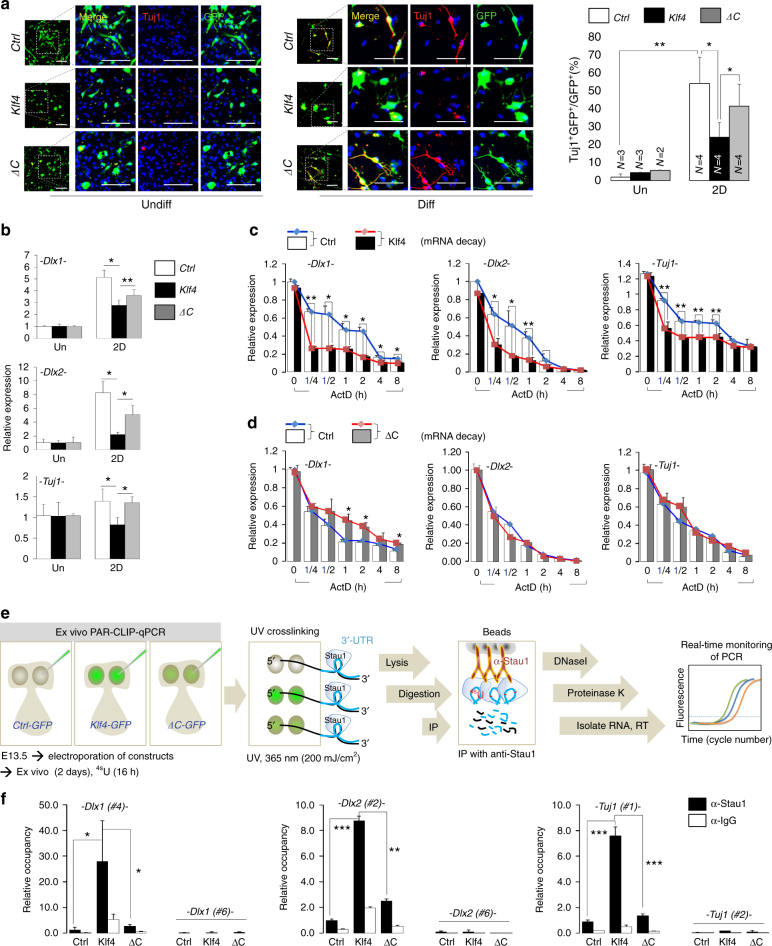
Klf4 increases Stau1 occupancy on target mRNAs in cortex ex vivo. **a** (Left) NPCs were transfected with pEGFP-Control, pEGFP-Klf4, or pEGFP-∆C vectors and grown for 0.2 or 2 days in N2 medium. Immunostaining to detect Tuj1 (red) or GFP (green) expression. DAPI (blue). Scale bar, 50 μm. (Right) Quantification of results shown at right. **b** qPCR analysis of indicated transcripts in NPCs transfected with pEGFP-Control, pEGFP-Klf4, or pEGFP-∆C constructs and cultured in undifferentiation (Un) conditions or for 2 days of differentiation (2D). **a**, **b** Data are presented as mean ± SD. ANOVA tests were performed to calculate significance (*P < 0.01, **P < 0.001). See Supplementary Table 4. **c**, **d** NPCs transfected with full-length (**c**) or ∆C Klf4 (**d**) were treated with actinomycin D for indicated times and mRNAs were prepared for RNA stability assay (*n* = 3). Results are normalized to control NPCs at 0 h. **e** Schematic showing the modified ex vivo PAR-CLIP protocol. Green images indicate GFP-labeled control, full-length Klf4 or ∆C expression in electroporated embryo brain. **f** PAR-CLIP-qPCR analysis in cortices electroporated with pEGFP-Control, pEGFP-Klf4, or pEGFP-∆C constructs and grown in ex vivo condition for 2D. See also Supplementary Fig. 13c

To investigate the role of Klf4 and Stau1 interaction in neurogenesis and SMD, cultured NPCs were transfected with full-length *Klf4* (*Klf4-GFP*) or the mutant *Klf4* lacking C terminus (*Klf4-*Δ*C-GFP*), which cannot interact with Stau1, and then cultured under differentiation condition for 2 days. The percentage of GFP/Tuj1 double-positive cells among all GFP-positive cells significantly decreased in cultures expressing full-length Klf4 compared to cultures expressing the Δ*C* mutant (Fig. 7a). Likewise, *Dlx1*, *Dlx2*, and *Tuj1* expression was significantly attenuated in Klf4-overexpressing NPCs, but not in cells overexpressing the Δ*C* mutant (Fig. 7b). We next compared wild-type or mutant (Δ*C*) *Klf4* function in neurogenesis using an ex utero electroporation system. Control vector, wild-type *Klf4*, or its mutant (Δ*C*) vectors expressing GFP were electroporated together with *shscramble* or *shKlf4* lentiviral vectors containing puromycin resistance selection marker into E13.5 embryos. The shRNA of *Klf4* targets 3′-UTR of *Klf4* mRNA and therefore can only knockdown endogenous *Klf4* with no effect on the overexpressed *Klf4*. After treatment of puromycin for 2 days, cultured embryos were readily identifiable by EGFP expression (Supplementary Fig. 13a). Quantitative analyses showed that the number of GFP/Tuj1 double-positive cells among all GFP-positive cells significantly increased in VZ/SVZ/IZ region in *shscramble* group compared to *shKlf4* group, and this increase in neurogenesis was rescued by overexpression of wild-type *Klf4* but not its mutant (Δ*C*) form (Supplementary Fig. 13b). To further assess effects of full-length or mutant *Klf4* on degradation of neurogenesis-associated mRNAs, we carried out an mRNA decay assay following expression of both constructs in NPCs. Klf4 overexpression significantly promoted degradation of target mRNAs, while we observed little change in the degradation rate of target mRNAs following expression of the Δ*C* mutant (Fig. 7c, d). This observation suggests that Klf4-mediated target mRNA decay requires interaction of Klf4 with Stau1. As noted (Fig. 5b), Stau1 binds *Dlx1*, *Dlx2*, and *Tuj1* mRNAs in NPCs, and this association decreases in NPCs lacking Klf4 (Fig. 6d). Thus, we asked whether decreased neuronal gene expression and up-regulated mRNA decay seen following Klf4 overexpression paralleled with Stau1 enrichment on target mRNAs. We also asked whether mutant Klf4 could affect Stau1 occupancy on these target mRNAs. To do so, we performed in vivo PAR-CLIP qPCR using ex vivo culture of cortices derived from embryos electroporated with constructs harboring full-length or mutant (Δ*C*) *Klf4* conjugated to *EGFP* (Supplementary Fig. 13c). Slices of EGFP-positive cortices were analyzed using a PAR-CLIP qPCR protocol modified for ex vivo cultured tissue (Fig. 7e; Supplementary Fig. 13c, right panel). Our analysis revealed a significant enrichment of Stau1 on the 3′-UTRs of *Dlx1*, *Dlx2*, and *Tuj1* mRNAs in NPCs expressing EGFP-Klf4 relative to the EGFP control vector (Fig. 7f). Not surprisingly, Stau1 occupancy on these mRNAs in NPCs expressing the *EGFP-∆C* construct was comparable to that of control (Fig. 7f). These *in vivo* findings suggest that Klf4 promotes Stau1 recruitment to neurogenesis-associated mRNAs, which subsequently promotes mRNA decay during corticogenesis.

## Discussion

In this study, we assessed how Klf4 and its interaction partner Stau1 function in neurogenesis. We found that Klf4 is down-regulated as NPCs differentiate, an event critical for neurogenesis. Using neural progenitor-specific Klf4 KO (*Nestin-Cre;Klf4*^*−/−*^) mice, we showed that Klf4 depletion enhances neurogenesis, as evidenced by a higher number of deep-layer mature neurons at cortical plates. At the same time, we observed decreased numbers of Nestin and Ki67 double-positive proliferating progenitor population and Pax6-positive apical progenitor population in the (sub)ventricular zone of *Klf4 cKO* mice, suggesting that enhanced neurogenesis is accompanied by Klf4 depletion. On the other hand, Klf4 overexpression inhibited neurogenesis and partially increased NPC proliferation.

Our MS analysis and co-IP identified Stau1 as a Klf4 interacting partner in NPCs. This is of particular interest because its homolog, Stau2, functions in NPC asymmetric cell division and lineage progression^27,28^. Relevant to the former, during cell division, Stau2 and its cargo mRNAs asymmetrically segregate into one of two daughter cells and induce cell cycle exit and differentiation of that cell. Although Stau1 functions in biological processes as varied as myogenesis, adipogenesis, and cutaneous wound healing^29–31^, its role in early stage of corticogenesis has not been clear. Recent reports^40,41^ using hiCLIP and RIP sequencing technologies in HEK293T cells showed that Stau1 binds to a subset of mRNAs that exhibit specific secondary structures. We investigated enrichment of Stau1 in several neuronal differentiation-associated mRNAs, and among these mRNAs, we chose *Dlx1*, *Dlx2*, and *Tuj1*, all of which contain high enrichment peak of Stau1 binding and play important roles in early neurogenesis. Here, we show that *Dlx1*, *Dlx2*, and *Tuj1* expression is up-regulated following Stau1 knockdown and down-regulated following Stau1 overexpression, indicating overall that Stau1 inhibits NPC differentiation. In support for this idea, our PAR-CLIP qPCR results and mRNA stability assay showed that Stau1 recognizes and binds to *Dlx1*, *Dlx2*, and *Tuj1* mRNAs and mediates their decay. Furthermore, the Klf4 C-terminal zinc-finger domain is required for binding to Stau1 and decay of target mRNAs, as evidenced by the fact that a deletion mutant of Klf4 lacking the C terminus does not co-immunoprecipitate with Stau1 or rescue neuronal differentiation phenotypes in *Klf4* KO NPCs, while other Klf4 deletion mutants do.

Interestingly, our MS results also identified Ddx5 and Ddx17 as Klf4 interacting proteins. Ddx5 (p68) and Ddx17 (p72) are DEAD box family RNA helicases^46–48^. Like Stau, both Ddx5 and Ddx17 function in many aspects of RNA processing and transcriptional regulation, such as coactivation of nuclear hormone receptors, p53 tumor suppressor, MyoD and Runx2^37,49^. Our co-IP analysis confirmed interaction between Klf4 and Ddx5/17, and our RNA stability assay showed that Ddx5 and Ddx17, like Stau1, participate in degradation of neurogenesis-related mRNAs. In SMD, after Stau1 recognizes and binds to a specific SBS, the RNA helicase UPF1 is recruited to unwind mRNA secondary structure, allowing decay to proceed^32^. We did not see an interaction between Klf4 and UPF1, but given that SMD requires RNA helicase activity, we propose that NPCs may utilize a novel SMD mechanism in which Ddx5 or Ddx17 is recruited to the adaptor protein Stau1 and its target mRNA and functions as an RNA helicase, a process regulated by Klf4 (Supplementary Fig. 14).

Finally, our observation that Klf4 inhibits neurogenesis but maintains NPC self-renewal differs from that reported by Qin et al.^19^, who showed that Klf4 overexpression inhibited both proliferation and differentiation in NPCs. These findings seem to be in conflict with ours; however, systemic difference used in studies may contribute to this discrepancy. Both of our studies used different systems: Qin et al.^19^ used *Klf4* transgenic mice to exam the role of dysregulated Klf4 in vivo, while we used *Nestin-cre:Klf4* *cKO* mice to evaluate the effect of Klf4 deletion in NPCs. Overexpression systems occasionally lead to aberrant phenotype^50^; therefore, it is not surprising that reduced self-renewal capacity was observed in NPCs expressing Klf4 ectopically. In this study, we found that Klf4 expression significantly reduced during neuronal differentiation of NPCs. In this respect, our reduced self-renewal capacity observed in *Klf4 cKO* NPCs compared to *WT* NPCs may reflect normal physiological event. Moreover, Stau1 gene KO mice exhibited dendritic spine morphogenesis defects in the adult brain with no strong neurogenesis phenotype^39^. This discrepancy could be due to different genetic models. In addition, the Stau1 KO mice is a gene deletion mutant, in which a 50 kDa truncation mutant protein is still expressed. Although the truncated protein with the two putative RBDs is incapable of binding RNA, this may contribute to this different effect in the developing brain.

In summary, we have demonstrated that Klf4 inhibits neurogenesis by regulating mRNA decay activity of Stau1. In NPCs, high levels of Klf4 allow Stau1 to bind to and degrade critical neurogenesis-associated mRNAs, allowing NPC self-renewal. During neurogenesis, while Stau1 level remains the same, reduced Klf4 level results in decreased SMD, allowing accumulation of these mRNAs (Supplementary Fig. 15). Our study reveals a novel mechanism associated with mRNA decay that regulates NPC self-renewal versus differentiation.

## Methods

### Animals

Generation of *Klf4*^*f/+*^ and *Klf4*^*f/f*^ mice was previously described^51^. Mice were maintained on a C57BL/6 background. To generate wild-type or *cKO* *Klf4* mouse embryos, *Nestin-cre* (*B6*.*Cg-Tg* (*Nes-cre*)*1Kln/J*; Jackson Lab, #003771) mice were crossed with either *Klf4*^*f/+*^ or *Klf4*^*f/f*^ mice. Offsprings were genotyped by PCR of genomic DNA extracted from mouse tail and analyzed with the following primers: F1, 5′-CTGGGCCCCCACATTAATGAG-3′; R1, 5′-CGCTGACAGCCATGTCAGACT-3′; and R2, 5′-CAGAGCCGTTCTGGCTGTTTT-3′. PCR was carried out using the S1000^TM^ Thermal Cycler (Bio-Rad, Hercules, CA, USA) with 2xTaq PCR premix (Bioland Scientific LLC, Paramount, CA, USA); conditions were 95 °C for 3 min followed by 34 cycles at 95 °C for 40 s, 60 °C for 40 s, and 72 °C for 1 min. In all experiments, *Klf4*^*fl/+*^, *Klf4*^*fl/+*^, and *Nestin-Cre:Klf4*^*fl/+*^ littermates served as controls. No significant phenotypic differences were seen in male versus female mice by blind analysis; thus, both genders were included in the analysis, randomizely. All mice were housed under a 12-h light–dark cycle and had ad libitum access to food and water in a controlled animal facility. All animal experimental procedures were approved by the Institutional Animal Care and Use Committee (IACUC) of the University of Southern California (protocol number, 11489) and the National Institutes of Health.

### Plasmids and shRNA transfection

To construct the lentiviral vector *pCDH-FLAG-Klf4*, which encodes N-terminally Flag-tagged Klf4 protein, *pFlag-Klf4* was amplified using 5′-TCTACCGCTAGCATGGACTACAAAGACGATGAC-3′ (sense) and 5′-TGGGATGGATCCTTAAAAGAGCCTCTTCATGTG-3′ (antisense) primers, where nucleotides contain overhangs to specify *Nhe*I and *Bam*HI sites, respectively. The fragment was digested with *Nhe*I and *Bam*HI and inserted into similarly digested *pCDH* vector. To construct *pCDH-Stau1-HA*, which encodes Stau1 protein with three tandem C-terminal HA tags, *pcDNA-Stau1-HA* was amplified using 5′-CTGTGCGCTAGCATGAAACTTGGAAAAAAACCA-3′ (sense) and 5′-AGATTATTCGAATCAGCGGCCGCACTGAGCAGC-3′ (antisense) primers, where nucleotides contain overhang to specify *Nhe*I and *Bst*BI sites, respectively. The resulting fragment was digested with *Nhe*I and *Bst*BI and inserted into similarly digested *pCDH* vector. NPCs were transfected with 4 μg *pCDH-Stau1-HA* or *pCDH-Klf4-Flag* for respective gain-of-function experiments or with *pLKO*.*1-shStau1* or *pLKO*.*1-shKlf4* for loss-of-function experiments using Lipofectamine^®^ LTX and Plus Reagent (Invitrogen, Carlsbad, CA, USA) or electroporation with an AMAXA nucleofector (Ronza AG, Basel, Switzerland). shRNAs were targeted at least two different coding sequence (CDS) or noncoding sequence (3′-UTR) regions. *pLKO*.*1-shScramble* vector serveds as the negative control in assays of *pLKO*.*1-shStau1* or *pLKO*.*1-shKlf4*. shRNA primer sequences were listed in Supplementary Table 1.Please check,the explanation mentions "where underlined nucleotides", but is not present in the text.We revised it.

### Preparation and culture of NPCs

NPCs were prepared from E11.5 cortices of wild-type or *Nestin-Cre:Klf4* *cKO* mice in Hank’s balanced salt solution (Invitrogen) and cultured as described^52,53^. NPCs were cultured in N2 medium containing bFGF for 4 days and then evaluated for Nestin and Sox2 expression. To induce differentiation, cells were seeded and further cultured without bFGF. Neurosphere culture was conducted as described^52–54^.

### Lentivirus production and viral transduction

Lentivirus was produced as described^55^. Briefly, HEK293T cells were transfected with the lentiviral packaging plasmid *psPAX2*, the envelope plasmid *pMD2*.*G*, and the target plasmid, using polyethylenimine (Polyscience). Culture medium was collected 48 h later and ultra-centrifuged (28,000 r.p.m., 90 min) to obtain high titer virus. Pelleted virus was re-suspended in 200 μl phosphate-buffered saline (PBS) at approximately 10^8^ TU/ml. For viral transduction, NPCs were plated onto poly-l-ornithine/fibronectin-coated plates in N2 medium. NPCs were transduced at a multiplicity of infection of 0.5–1 together with polybrene (Sigma-Aldrich) for 24 h and selected in puromycin 48 h after transduction.

### Antibodies and reagents

Antibodies used in this study were anti-Klf4 (rabbit polyclonal 1:500; Gene Tex, Irvine, CA, USA), anti-Flag (mouse, 1:1000; Sigma, St. Louis, MO, USA), anti-HA (rabbit, 1:1000; Santa Cruz Biotechnology, Santa Cruz, CA, USA), anti-Stau1 (rabbit, 1:500; Thermo Scientific, Rockford, IL, USA), anti-Map2 (rabbit polyclonal 1:200, Chemicon, Temecula, CA, USA), anti-Nestin (mouse monoclonal 1:350, BD Biosciences, San Jose, CA, USA), anti-Tuj1 (mouse polyclonal 1:200; Covance, Princeton, NJ, USA), anti-Tbr1 (rabbit polyclonal 1:200; Abcam Ltd, Cambridge, MA, USA), anti-Pax6 (rabbit polyclonal 1:200; Abcam Ltd), anti-Ddx5 (rabbit polyclonal 1:1000; Abcam Ltd), anti-Ddx17 (rabbit polyclonal 1:1000; Abcam Ltd), anti-GST (rabbit polyclonal 1:500; Santa Cruz Biotechnology), anti-BrdU (mouse monoclonal 1:500; Abcam Ltd), anti-Dlx1 (mouse monoclonal 1:250; Abnova, Walnut, CA, USA), anti-Dlx2 (rabbit polyclonal, 1:250; Abcam Ltd), anti-Ctip2 (Rat 1:200; Abcam Ltd), anti-cleaved caspase-3 (mouse 1:200; R&D Systems, Inc., Minneapolis, MN, USA), anti-UPF1 (rabbit 1:200; Cell Signaling Technology Inc., Danvers, MA, USA), and anti-α-tubulin (mouse monoclonal 1:5000; Santa Cruz Biotechnology). Secondary antibodies were anti-rabbit Alexa Fluor 488-conjugated, anti-mouse Alexa Fluor 488-conjugated, anti-rabbit Alexa Fluor 555-conjugated, or anti-mouse Alexa Fluor 555-conjugated IgG (1:200 dilution; Molecular Probes, Eugene, OR, USA). bFGF was purchased from PeproTech (Rocky Hill, NJ, USA). The protease inhibitor cocktail was from Roche Applied Science (Indianapolis, IN, USA). To inhibit RNA synthesis, actinomycin D was purchased from VWR International, LLC (San Dimas, CA, USA). The enhanced chemiluminescence (ECL) Kit and KOD Hot Start DNA polymerase were from EMD Millipore. Phenol:chloroform:isoamyl alcohol and the First Strand cDNA Synthesis Kit were from Thermo Scientific.

### Immunoprecipitation, Western blotting, and MS analysis

Cells were gently lysed with IP buffer (50 mM Tris-HCl, pH 7.4, 130 mM NaCl, 10 mM NaF, 2 mM EGTA, 2 mM EDTA, 0.5% Triton X-100, 0.5% NP-40, 5 % glycerol, 1 mM dithiothreitol (DTT), and a protease inhibitor cocktail) for 1 h on ice and then centrifuged at 14,000 r.p.m. at 4 °C for 15 min. Supernatants were collected and precleared with 30 μl of Protein A/G beads (Santa Cruz Biotechnology) for 2 h, and then precleared lysates were incubated with 4 μg of each specific antibody overnight at 4 °C. After immune complexes were washed six times with IP buffer, they were eluted by boiling for 3 min at 95 °C in sodium dodecyl sulfate (SDS) sample buffer and separated on 10% SDS-polyacrylamide gel electrophoresis (SDS-PAGE). After blocking, membranes were sequentially incubated with corresponding primary and horse radish peroxidase-conjugated secondary antibodies (anti-mouse or anti-rabbit 1:10,000; Santa Cruz Biotechnology). Protein bands were detected using ECL reagent (Santa Cruz Biotechnology). NPCs stably expressing Flag-Klf4 were used for MS analysis as described^56^. Briefly, anti-Flag antibody was used to immunoprecipitate Flag-Klf4 and associated proteins, and anti-IgG served as control. After separating immunoprecipitated proteins on 10% SDS-PAGE and silver staining, bands of interest were excised, washed, and subjected to MS analysis.

### Immunocytochemistry and immunohistochemistry

Cells grown on coverslips were fixed with 4% paraformaldehyde (PFA) for 10 min, permeabilized with 0.2% Triton X-100, incubated with blocking solution containing 5% bovine serum albumin (BSA) in PBS for 30 min, and incubated with primary antibodies overnight at 4 °C. After PBS washing, cells were incubated with secondary antibodies at room temperature for 1 h and counterstained with 4',6-diamidino-2-phenylindole (DAPI). For embryonic brain tissues, samples were fixed with 4% PFA overnight and cryopreserved in 30% sucrose for 2 more days before embedding in OCT compound (Tissue Tek, Torrance, CA, USA). Samples were sectioned on a cryostat at 20 μm. Slides were blocked with 5% BSA at room temperature for 30 min before staining with primary antibody overnight. Slides were then incubated with secondary antibody for 1 h at room temperature after three PBS washes for 10 min each and counterstained with DAPI. Stained slides were washed three times with PBS before mounting. Images were obtained using a fluorescent confocal microscope (LSM5 PASCAL; Zeiss, Jena, Germany). Values obtained from at least three independent experiments were averaged and reported as means ± SD. The two-tailed Student’s *t* test was used to compare two experimental groups.

### Reverse transcriptase qPCR

Cells were harvested and total RNA was isolated using TRIzol reagent (Invitrogen). A SuperScript III qRT-PCR Kit (Invitrogen) was used to synthesize cDNA from total RNA. qPCR was performed using a ViiA7 system (Applied Biosystems) with iTaq Universal SYBR Green Master Mix (Bio-Rad); conditions were 95 °C for 10 min followed by 50 cycles at 95 °C for 15 s and 60 °C for 3 s. Samples were run in triplicate and *Klf4*, *Stau1*, *Nestin*, *NeuN*, *Dlx1*, *Dlx2*, *Tuj1*, and *Gad67* transcripts were quantitated by comparing cycle threshold (Ct) values for each reaction with a *Gapdh* reference. Primer sets for quantitative PCR are listed in Supplementary Table 2.

### mRNA degradation analysis

NPCs were treated with 5 μg/ml of actinomycin D (VWR) for 15 min, 30 min, and 1, 2, 4, or 8 h. RNA was extracted using TRIzol reagent (Invitrogen) according to the manufacturer’s instructions. qPCR was performed as described, with *Gapdh* serving as normalization control. Primer sequences for qPCR are listed in Supplementary Table 2. Statistical significance of changes in mRNA half-life were determined using a *t* test.

### In vitro and ex vivo PAR-CLIP qPCR analysis

PAR-CLIP analysis was performed as described^57^. For in vitro analysis, NPCs were incubated in medium supplemented with 100 μM 4-thiouridine (SU) for 16 h, washed with PBS, and irradiated with 150 mJ/cm^2^, 365 nm ultraviolet light in a Spectrolinker XL-1500 UV crosslinker to crosslink RNA to Stau1. Alternatively, E13.5 embryos derived from total four pregnant mice (E13.5) were electroporated with GFP-control, GFP-Klf4, and GFP-Klf4 (∆C) vectors and grown ex vivo for 2 days in N2 medium with bFGF. After incubation in 200 μM 4-thiouridine (SU) for 16 h, embryos were dissected to obtain forebrain cortices and tissues were irradiated with 200 mJ/cm^2^, 365 nm ultraviolet light in a Spectrolinker XL-1500 UV crosslinker to crosslink RNA to endogenous Stau1. Samples were harvested and lysed in an equivalent of three pellet volumes of NP-40 lysis buffer. Cleared cell or tissue lysates were treated with 1 U/μl RNase T1 (Thermo Fisher Scientific, Fremont, CA, USA), and endogenous Stau1 protein was immunoprecipitated with polyclonal anti-Stau1 antibody (Thermo Fisher Scientific) bound to Protein A/G Dynabeads. RNA in immunoprecipitates was further trimmed with 100 U/ml RNase T1. Beads were washed in lysis buffer, and DNA and proteins were removed by digestion with DNase I (Zymo Research Corporation, Orange, CA, USA) and 0.2 mg/ml proteinase K in proteinase K buffer (Thermo Fisher Scientific), respectively. RNA was recovered by acidic phenol/chloroform extraction and ethanol precipitation and used in qPCR analysis. Primer sets for PAR-CLIP qPCR are listed in Supplementary Table 3.

### In utero and ex utero electroporation

All procedures followed guidelines of the IACUC and the National Institutes of Health. A total of 2.5% (w/v) avertin (1 g/ml solution of 2,2,2-Tribromoethanol, 97% in *tert*-amyl alcohol (99%); Aldrich, catalog nos. T4, 840-2 and 24, 048-6, respectively) in 0.9% saline was injected i.p. (15 µl/g of body weight) to anesthetize total 12 pregnant mice (E13.5). A laparotomy was performed, and the uterus with embryos was exposed. A total of 2–5 µl of plasmid DNA (approximately 2 µg/µl, dissolved in water) was injected into the lateral ventricle using a fine-glass microcapillary using a PV830 pneumatic PicoPump. Electroporation was performed using a Nepagene CUY21SC electroporator (amplitude, 50 V (E13.5); duration, 50 ms; intervals, 150 ms). To deliver electrical pulses, tweezer-type circular electrodes (7 mm diameter) were used with the positive side directed to the medial wall of the ventricle into which DNA was injected. Uterine horns were repositioned in the abdominal cavity, and the abdominal wall and skin were sewed with surgical sutures. Mice were kept on a warm plate (37 °C) for recovery. For ex utero experiment, embryos were obtained from a total of six pregant mice (E13.5) and E13.5 embryos were electroporated with GFP-control, GFP-Klf4, and GFP-Klf4 (∆C) together with pLKO.1-shscramble or pLKO.1-shKlf4 lentiviral vectors. Embryos were grown in N2:B27 (1:1) medium with bFGF (20 ng/ml) during 1 day and then further incubated with selection medium containing puromycin (5 μg/ml) for 48 h. Embryos were then fixed with 4% (w/v) PFA (Sigma) in PBS (pH 7.4). After a 24 h fixation at 4 °C, embryo brains were transferred to a 30% (w/v) sucrose solution in 4% PFA. Tissues were sectioned at 30 µm using a cryotome (Leica) and analyzed by immunohistochemistry.

### Statistical analysis

Statistical analyses were performed using Excel statistical tools or Prism 6 (GraphPad Software). Where differences between treatment groups were experimentally hypothesized, statistical differences among two groups were analyzed using Student’s *t* test (*P < 0.05, **P < 0.005, and ***P < 0.0005). Analysis of variance (ANOVA) tests (Tukey’s multiple comparison test) were used to test hypotheses about effects in multiple groups. Differences are indicated in figures as follows: *P < 0.01, **P < 0.001, and ***P < 0.0001. *P < 0.01 was considered statistically significant.

### Data availability

All information that supports the findings of this study are obtained from our experimental subjects and the data are available from the corresponding author on reasonable request.

## Electronic supplementary material

Supplementary Information

## References

[1] Paridaen JT, Huttner WB (2014). Neurogenesis during development of the vertebrate central nervous system. EMBO Rep..

[2] Huttner WB, Kosodo Y (2005). Symmetric versus asymmetric cell division during neurogenesis in the developing vertebrate central nervous system. Curr. Opin. Cell Biol..

[3] Balu DT, Lucki I (2009). Adult hippocampal neurogenesis: regulation, functional implications, and contribution to disease pathology. Neurosci. Biobehav. Res..

[4] Kempermann G, Krebs J, Fabel K (2008). The contribution of failing adult hippocampal neurogenesis to psychiatric disorders. Curr. Opin. Psychiatr..

[5] Winner B, Kohl Z, Gage FH (2011). Neurodegenerative disease and adult neurogenesis. Eur. J. Neurosci..

[6] Dai W (2015). A post-transcriptional mechanism pacing expression of neural genes with precursor cell differentiation status. Nat. Commun..

[7] Kiebler MA, Scheiffele P, Ule J (2013). What, where, and when: the importance of post-transcriptional regulation in the brain. Front. Neurosci..

[8] McConnell BB, Yang VW (2010). Mammalian Kruppel-like factors in health and diseases. Physiol. Rev..

[9] Garrett-Sinha LA, Eberspaecher H, Seldin MF, de Crombrugghe B (1996). A gene for a novel zinc-finger protein expressed in differentiated epithelial cells and transiently in certain mesenchymal cells. J. Biol. Chem..

[10] Segre JA, Bauer C, Fuchs E (1999). Klf4 is a transcription factor required for establishing the barrier function of the skin. Nat. Genet..

[11] Shields JM, Christy RJ, Yang VW (1996). Identification and characterization of a gene encoding a gut-enriched Kruppel-like factor expressed during growth arrest. J. Biol. Chem..

[12] Jiang J (2008). A core Klf circuitry regulates self-renewal of embryonic stem cells. Nat. Cell Biol..

[13] Li Y (2005). Murine embryonic stem cell differentiation is promoted by SOCS-3 and inhibited by the zinc finger transcription factor Klf4. Blood.

[14] Takahashi K, Yamanaka S (2006). Induction of pluripotent stem cells from mouse embryonic and adult fibroblast cultures by defined factors. Cell.

[15] Takahashi K (2007). Induction of pluripotent stem cells from adult human fibroblasts by defined factors. Cell.

[16] Moore DL (2009). KLF family members regulate intrinsic axon regeneration ability. Science.

[17] Qin S, Zou Y, Zhang CL (2013). Cross-talk between KLF4 and STAT3 regulates axon regeneration. Nat. Commun..

[18] Qin S, Zhang CL (2012). Role of Kruppel-like factor 4 in neurogenesis and radial neuronal migration in the developing cerebral cortex. Mol. Cell. Biol..

[19] Qin S, Liu M, Niu W, Zhang CL (2011). Dysregulation of Kruppel-like factor 4 during brain development leads to hydrocephalus in mice. Proc. Natl. Acad. Sci. USA.

[20] Park E, Maquat LE (2013). Staufen-mediated mRNA decay. Wiley Interdiscip. Rev. RNA.

[21] Riechmann V, Ephrussi A (2001). Axis formation during Drosophila oogenesis. Curr. Opin. Genet. Gev..

[22] Sporik R, Johnstone JH, Cogswell JJ (1991). Longitudinal study of cholesterol values in 68 children from birth to 11 years of age. Arch. Dis. Child.

[23] Broadus J, Fuerstenberg S, Doe CQ (1998). Staufen-dependent localization of prospero mRNA contributes to neuroblast daughter-cell fate. Nature.

[24] de Lucas S, Oliveros JC, Chagoyen M, Ortin J (2014). Functional signature for the recognition of specific target mRNAs by human Staufen1 protein. Nucleic Acids Res..

[25] Kiebler MA (1999). The mammalian staufen protein localizes to the somatodendritic domain of cultured hippocampal neurons: implications for its involvement in mRNA transport. J. Neurosci..

[26] Miki T, Takano K, Yoneda Y (2005). The role of mammalian Staufen on mRNA traffic: a view from its nucleocytoplasmic shuttling function. Cell Struct. Funct..

[27] Kusek G (2012). Asymmetric segregation of the double-stranded RNA binding protein Staufen2 during mammalian neural stem cell divisions promotes lineage progression. Cell Stem Cell.

[28] Vessey JP (2012). An asymmetrically localized Staufen2-dependent RNA complex regulates maintenance of mammalian neural stem cells. Cell Stem Cell.

[29] Gong C, Kim YK, Woeller CF, Tang Y, Maquat LE (2009). SMD and NMD are competitive pathways that contribute to myogenesis: effects on PAX3 and myogenin mRNAs. Gene Dev..

[30] Cho H (2012). Staufen1-mediated mRNA decay functions in adipogenesis. Mol. Cell.

[31] Gong C, Maquat LE (2011). lncRNAs transactivate STAU1-mediated mRNA decay by duplexing with 3’ UTRs via Alu elements. Nature.

[32] Kim YK, Furic L, Desgroseillers L, Maquat LE (2005). Mammalian Staufen1 recruits Upf1 to specific mRNA 3’UTRs so as to elicit mRNA decay. Cell.

[33] Gong C, Tang Y, Maquat LE (2013). mRNA-mRNA duplexes that autoelicit Staufen1-mediated mRNA decay. Nat. Struct. Mol. Biol..

[34] Kim YK (2007). Staufen1 regulates diverse classes of mammalian transcripts. EMBO J..

[35] Gervais FG (1999). Involvement of caspases in proteolytic cleavage of Alzheimer’s amyloid-beta precursor protein and amyloidogenic A beta peptide formation. Cell.

[36] Maquat LE, Gong C (2009). Gene expression networks: competing mRNA decay pathways in mammalian cells. Biochem. Soc. Trans..

[37] Dardenne E (2014). RNA helicases DDX5 and DDX17 dynamically orchestrate transcription, miRNA, and splicing programs in cell differentiation. Cell Rep..

[38] Gustafson EA, Wessel GM (2010). DEAD-box helicases: post-translational regulation and function. Biochem. Biophys. Res. Commun..

[39] Vessey JP (2008). A loss of function allele for murine Staufen1 leads to impairment of dendritic Staufen1-RNP delivery and dendritic spine morphogenesis. Proc. Natl. Acad. Sci. USA.

[40] Ricci EP (2014). Staufen1 senses overall transcript secondary structure to regulate translation. Nat. Struct. Mol. Biol..

[41] Sugimoto Y (2015). hiCLIP reveals the in vivo atlas of mRNA secondary structures recognized by Staufen 1. Nature.

[42] Shaw G, Morse S, Ararat M, Graham FL (2002). Preferential transformation of human neuronal cells by human adenoviruses and the origin of HEK 293 cells. FASEB J..

[43] Lin YC (2014). Genome dynamics of the human embryonic kidney 293 lineage in response to cell biology manipulations. Nat. Commun..

[44] Isken O, Maquat LE (2008). The multiple lives of NMD factors: balancing roles in gene and genome regulation. Nat. Rev. Genet..

[45] Geissler V, Altmeyer S, Stein B, Uhlmann-Schiffler H, Stahl H (2013). The RNA helicase Ddx5/p68 binds to hUpf3 and enhances NMD of Ddx17/p72 and Smg5 mRNA. Nucleic Acids Res..

[46] Hirling H, Scheffner M, Restle T, Stahl H (1989). RNA helicase activity associated with the human p68 protein. Nature.

[47] Lamm GM, Nicol SM, Fuller-Pace FV, Lamond AI (1996). p72: a human nuclear DEAD box protein highly related to p68. Nucleic Acids Res..

[48] Uhlmann-Schiffler H, Rossler OG, Stahl H (2002). The mRNA ofDEAD box protein p72 is alternatively translated into an 82-kDa RNA helicase. J. Biol. Chem..

[49] Fuller-Pace FV (2013). DEAD box RNA helicase functions in cancer. RNA Biol..

[50] Prelich G (2012). Gene overexpression: uses, mechanisms, and interpretation. Genetics.

[51] Katz JP (2002). The zinc-finger transcription factor Klf4 is required for terminal differentiation of goblet cells in the colon. Development.

[52] Moon BS (2011). Sur8/Shoc2 involves both inhibition of differentiation and maintenance of self-renewal of neural progenitor cells via modulation of extracellular signal-regulated kinase signaling. Stem Cells.

[53] Moon BS (2017). Smek promotes corticogenesis through regulating Mbd3’s stability and Mbd3/NuRD complex recruitment to genes associated with neurogenesis. PLoS Biol..

[54] Kim MY, Moon BS, Choi KY (2013). Isolation and maintenance of cortical neural progenitor cells in vitro. Methods Mol. Biol..

[55] Ye Z, Yu X, Cheng L (2008). Lentiviral gene transduction of mouse and human stem cells. Methods Mol. Biol..

[56] Lyu J (2013). Protein phosphatase 4 and Smek complex negatively regulate Par3 and promote neuronal differentiation of neural stem/progenitor cells. Cell Rep..

[57] Yoon JH (2014). PAR-CLIP analysis uncovers AUF1 impact on target RNA fate and genome integrity. Nat. Commun..

