# Supplementary material for: Kruppel-like factor 4-dependent Staufen1-mediated mRNA decay regulates cortical neurogenesis
Source: Nat Commun. 2018 Jan 26;9:401. doi: 10.1038/s41467-017-02720-9 (PMC5785957; doi:10.1038/s41467-017-02720-9)
Supplement: Supplementary file 1 — Supplementary Information [file 41467_2017_2720_MOESM1_ESM.pdf]

SUPPLEMENTARY FIGURE LEGENDS

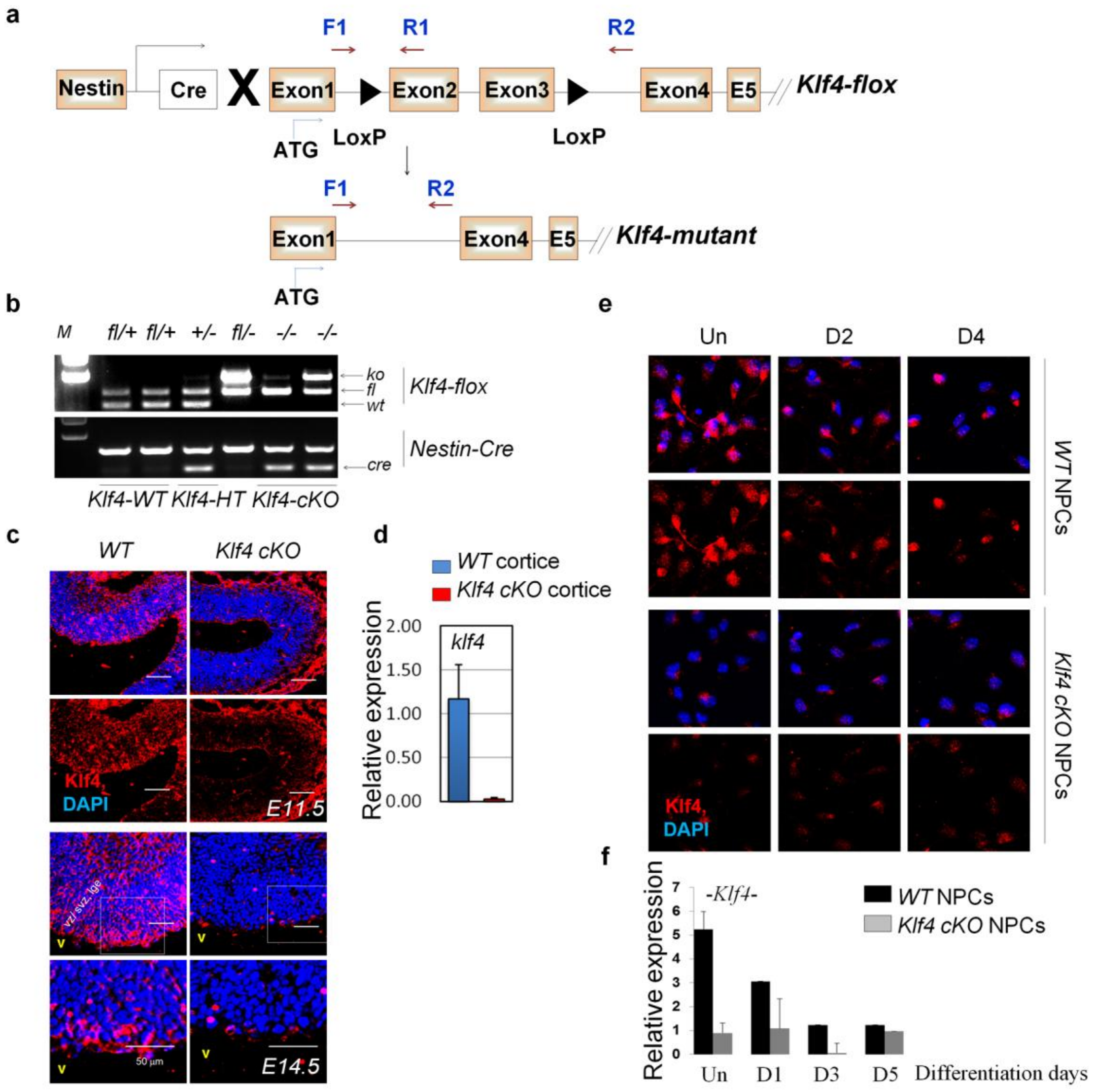

Supplementary Figure 1. Generation of Klf4 conditional knockout mice. (**a**, **b**) *Nestin-cre* mice were crossed with *Klf4*$^{fl/+}$ or *Klf4*$^{fl/fl}$ mice to generate *wild-type* and *Klf4* conditional knockout embryos. Genotyping was performed with indicated primers. (**b**) Upper band is Klf4 knockout, middle band is floxed, and lower band indicates wild-type. Left lane (*M*) indicates size

marker. (**c**) Fixed cyro-embedded coronal sections from E11.5 or E14.5 mouse forebrain stained with antibodies against Klf4 (red). DAPI (blue). Scale bars, 50 µm. (**d**) qPCR analysis of Klf4 mRNA cortices equivalent to those shown in (c, lower panel). (**e**) Immunostaining to detect Klf4 expression in *wild-type* and *Klf4 cKO NPCs*. Nuclear staining shown by DAPI (blue). Scale bar, 50 µm. (**f**) qPCR analysis of Klf4 levels in *wild-type* and *Klf4 NPCs* at indicated days (d) of differentiation (n=2-3).

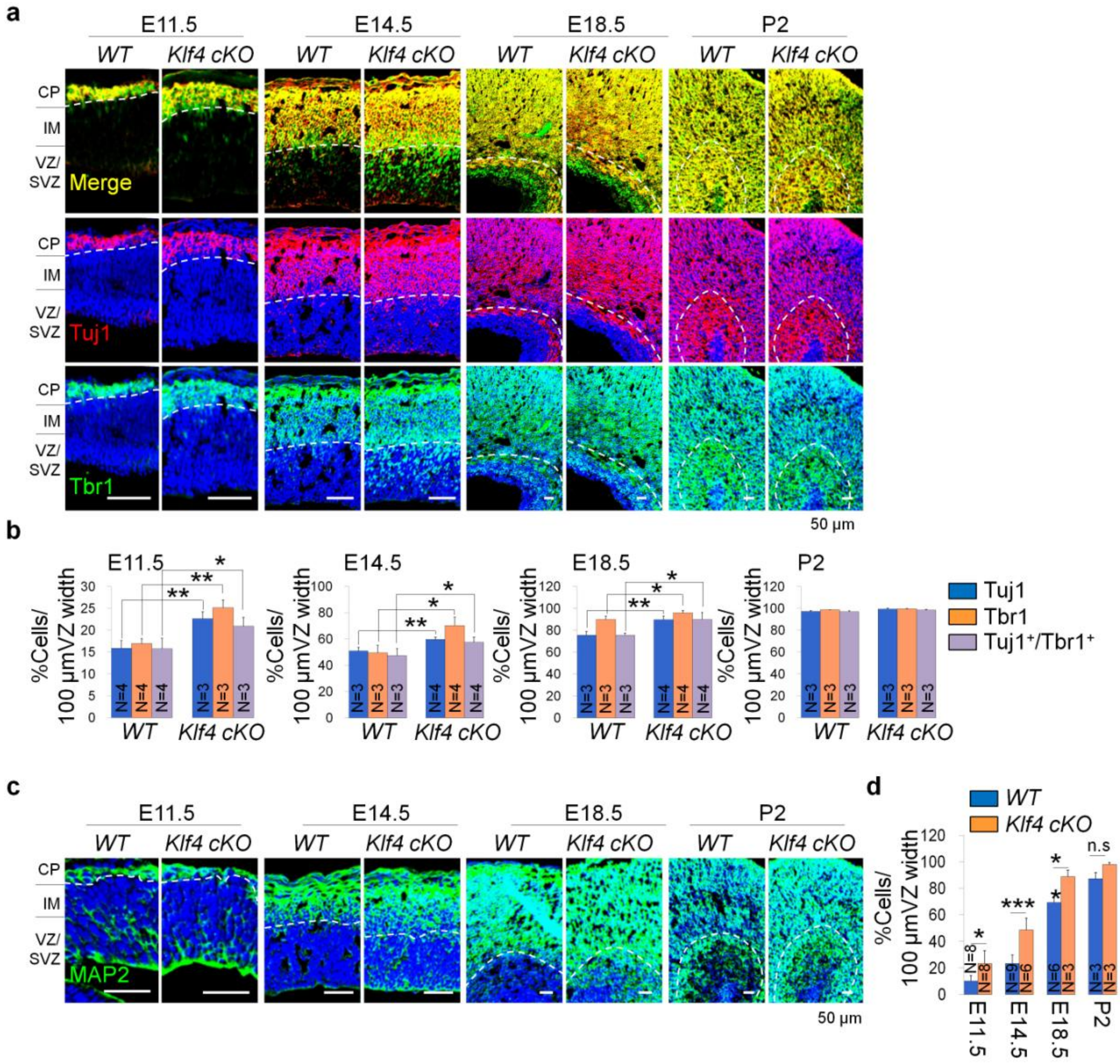

Supplementary Figure 2. Klf4 down-regulation enhances neurogenesis *in vivo*. (**a, c**) Fixed coronal sections from E11.5, E14.5, E18.5 or P2 mouse forebrain stained with antibodies against Tuj1 (red), Tbr1 (green) or Map2 (green). DAPI (blue). (**b, d**) Images shown above were quantified in cortex areas of 100 μm ventricular width extending from VZ to the pial surface (E11.5: *Klf4* $^{fl/+}$*(WT)*, n=4-8, *Klf4 cKO*, n=3-8, and E14.5: *Klf4*$^{fl/+}$ *(WT)*, n=3-9, *Klf4 cKO*, n=4-6, E18.5: *Klf4*$^{fl/+}$ *(WT)*, n=3-6, *Klf4 cKO*, n=3-4, P2: *Klf4*$^{fl/+}$ *(WT)*, n=3, *Klf4 cKO*, n=3). Scale bars, 50 μm. Values correspond to mean±SD. t-tests were performed to calculate significance (*P < 0.05, **P < 0.005, ***P < 0.0005).

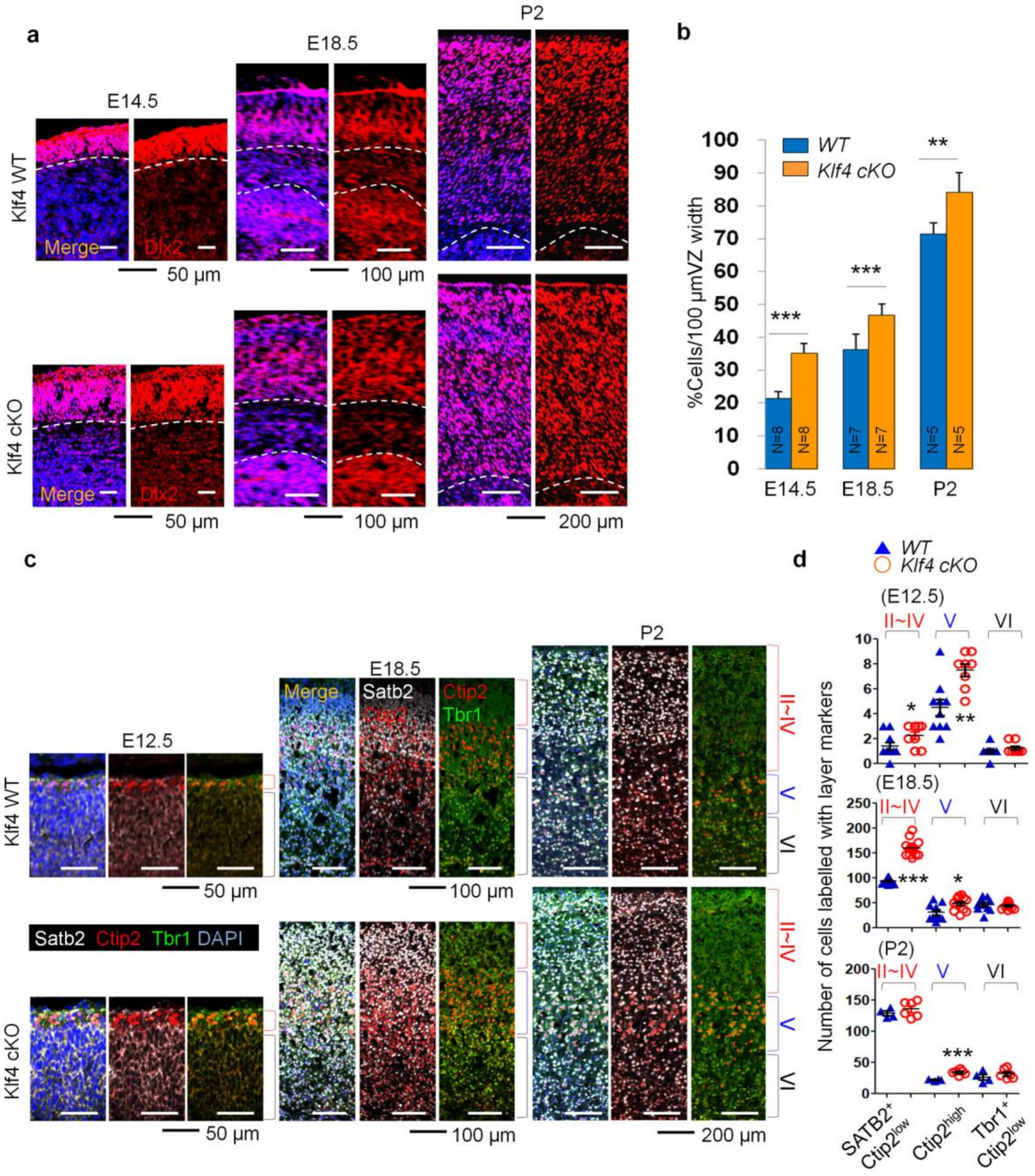

Supplementary Figure 3. Klf4 down-regulation enhances deep layer corticogenesis *in vivo*. (**a**) Fixed coronal sections from E14.5, E18.5 or P2 mouse forebrain stained with antibodies against Dlx2 (red). DAPI (blue). (**b**) Images shown in (a) were quantified in cortex in areas of 100 μm ventricular width extending from VZ to the pial surface in E14.5 (n=8/genotype), E18.5 (n=7/genotype), and P2 (n=5/genotype). (**c**) Representative immuno-labeling of E12.5, E18.5

and P2 cortical sections with indicated antibodies revealing cortical layering in *WT* and *Nestin*^{cre}*:Klf4 cKO* mice. (**d**) Quantification of (c) numbers over brackets denote cortical layers where the cells were counted. CTIP2^{low} (layer 6) and CTIP2^{high} (layer 5) expressing cells were used to differentiate between Tbr1^{+}/CTIP2^{low} cells of layer 5 and CTIP^{high} cells in layer 5. SATB2^{+}/CTIP2- cells were counted above layer of CTIP^{high} cells in E12.5 (n=8~10/genotype), E18.5 (n=10~12/genotype), and P2 (n=4~6/genotype). Values correspond to mean±SD. t-tests were performed to calculate significance (*P < 0.05, **P < 0.005, ***P < 0.0005).

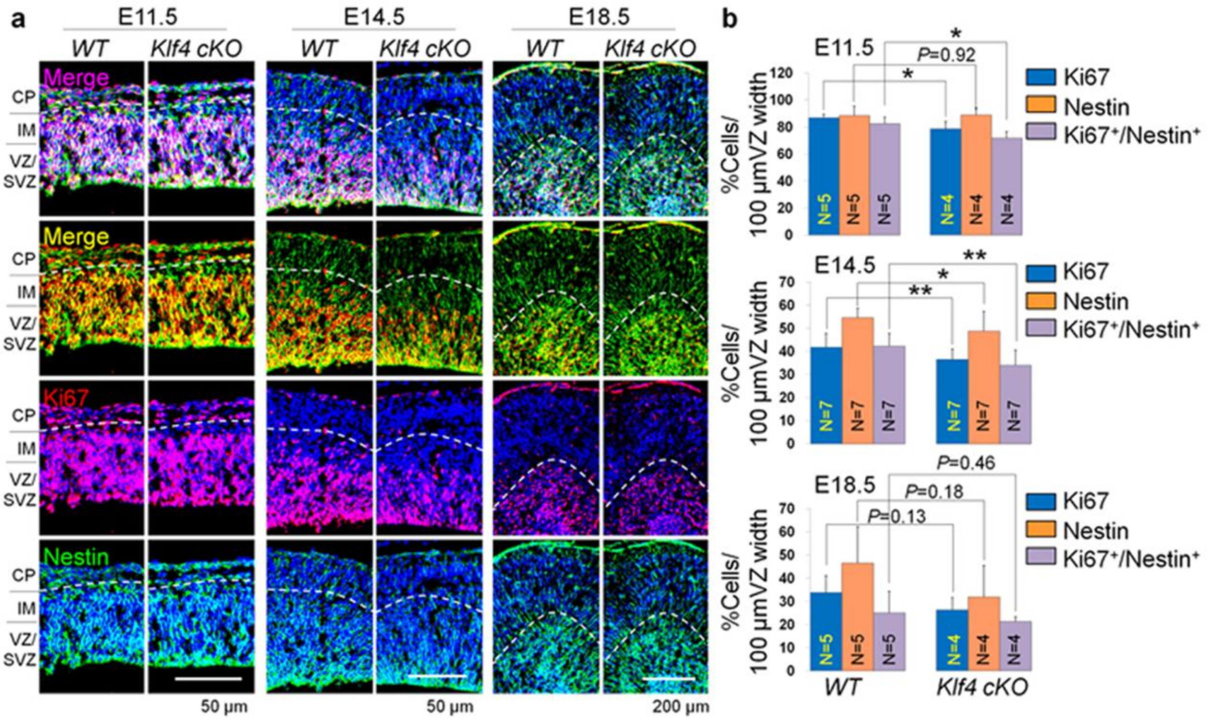

Supplementary Figure 4. Klf4 down-regulation enhances neuronal maturation and decreases proliferative neural progenitor population in developmental neocortex. (**a**) Fixed coronal sections from E11.5, E14.5, or E18.5 mouse forebrain stained with antibodies against Nestin (green) and Ki67 (red). DAPI (blue). (**b**) Images shown above were quantified in cortex

in areas of 100 μm ventricular width extending from VZ to the pial surface. Scale bars, 50 μm. Values correspond to mean±SD. *t*-tests were performed to calculate significance (*P < 0.05, **P < 0.005).

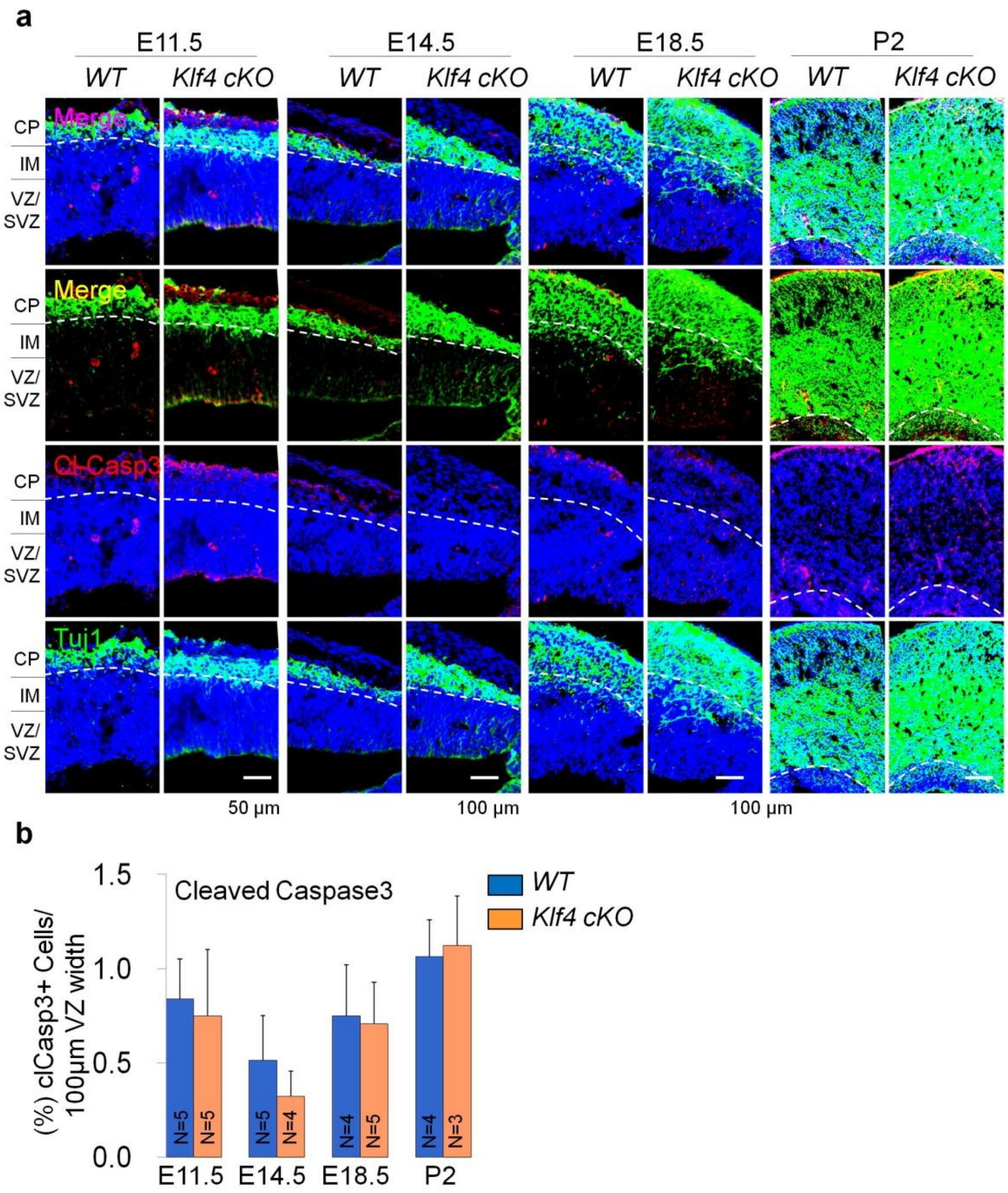

Supplementary Figure 5. Klf4 down-regulation does not change cell death of progenitors and neurons in developmental neocortex. (**a**) Representative immuno-labeling for apoptotic cells with clCasp3 and neuronal marker TuJ1 in coronal telencephalic sections from E11.5, E14.5, or P2 mice. (**b**) Apoptosis of cells were quantified in cortex in areas of 100 mm ventricular width

extending from VZ to the pial surface (E11.5: *Klf4* $^{fl/+}$*(WT)*, n=5, *Klf4 cKO*, n=5, and E14.5: *Klf4*$^{fl/+}$ *(WT)*, n=5, *Klf4 cKO*, n=4, E18.5: *Klf4*$^{fl/+}$ *(WT)*, n=4, *Klf4 cKO*, n=5, P2: *Klf4*$^{fl/+}$ *(WT)*, n=4, *Klf4 cKO*, n=3). Scale bars, 50 or 100 μm. Values correspond to mean±SD. *t*-tests were performed to calculate significance.

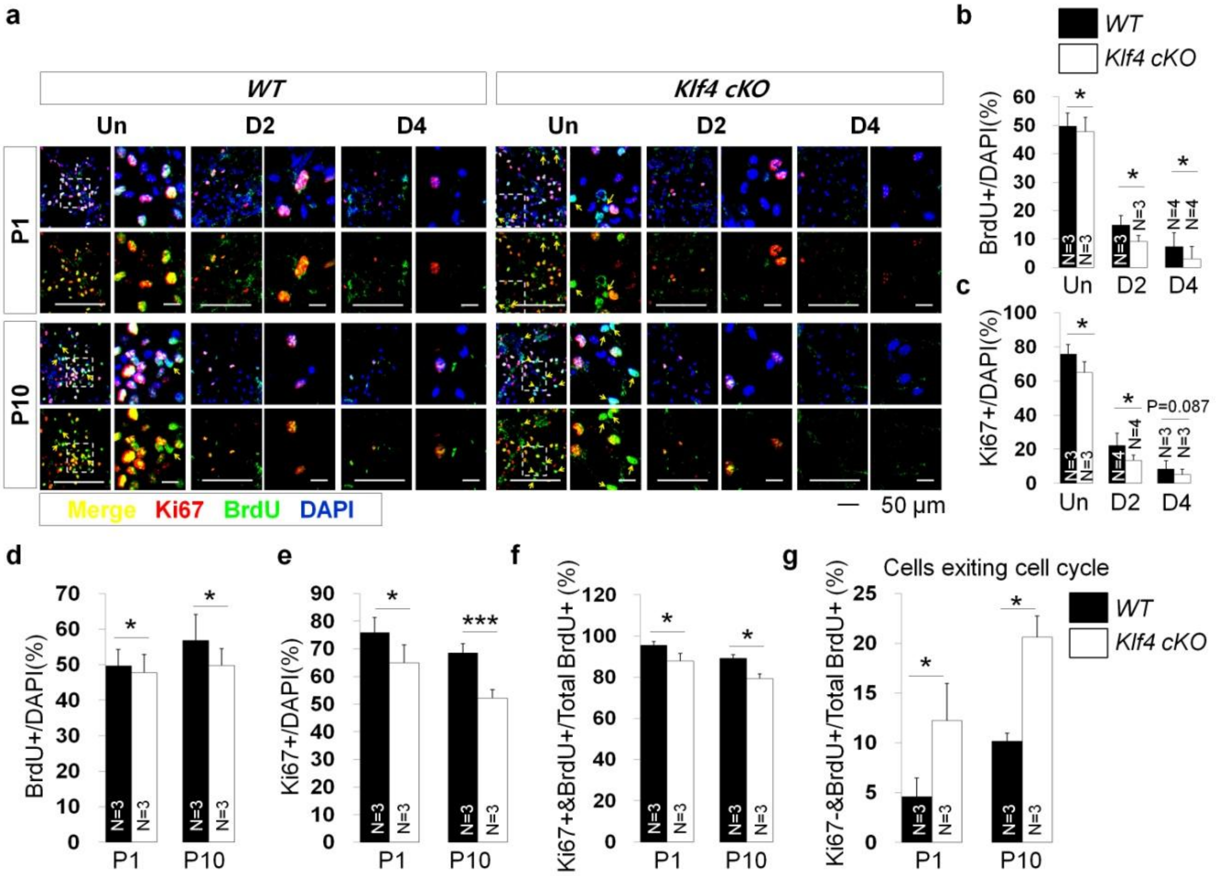

Supplementary Figure 6. Klf4 down-regulation decreases proliferative neural progenitor cells and increases cells exiting cell cycle. (**a**) Representative immuno-labeling for Ki67 and BrdU in the P1 (passage 1) or P10 (passage 10) NPCs pulsed with BrdU (30 μg/ml) for 5 hrs. The area outlined by dotted lines is magnified in the adjacent images. Un, undifferentiation; D2, differentiation 2 days *in vitro*; D4, differentiation 4 days *in vitro*. Yellow arrows indicate cells exiting cell cycle which are Ki67 negative cells incorporated BrdU in the S phase. (**b-e**) Quantification of (a). Histogram showing percent stained cells relative to the number of DAPI^{+}

nuclei. Scale bar, 50 μm. D, differentiation days *in vitro*. (**f**) Fraction of Ki67^{+}BrdU^{+} cells in total BrdU^{+} cell population. (**g**) Fraction of cells exiting the cell cycle defined as fraction of Ki67^{-} BrdU^{+} cells in total BrdU^{+} cell population. Scale bars, 50 μm. Values correspond to mean±SD. *t*-tests were performed to calculate significance (*P < 0.05, **P < 0.005, ***P < 0.0005).

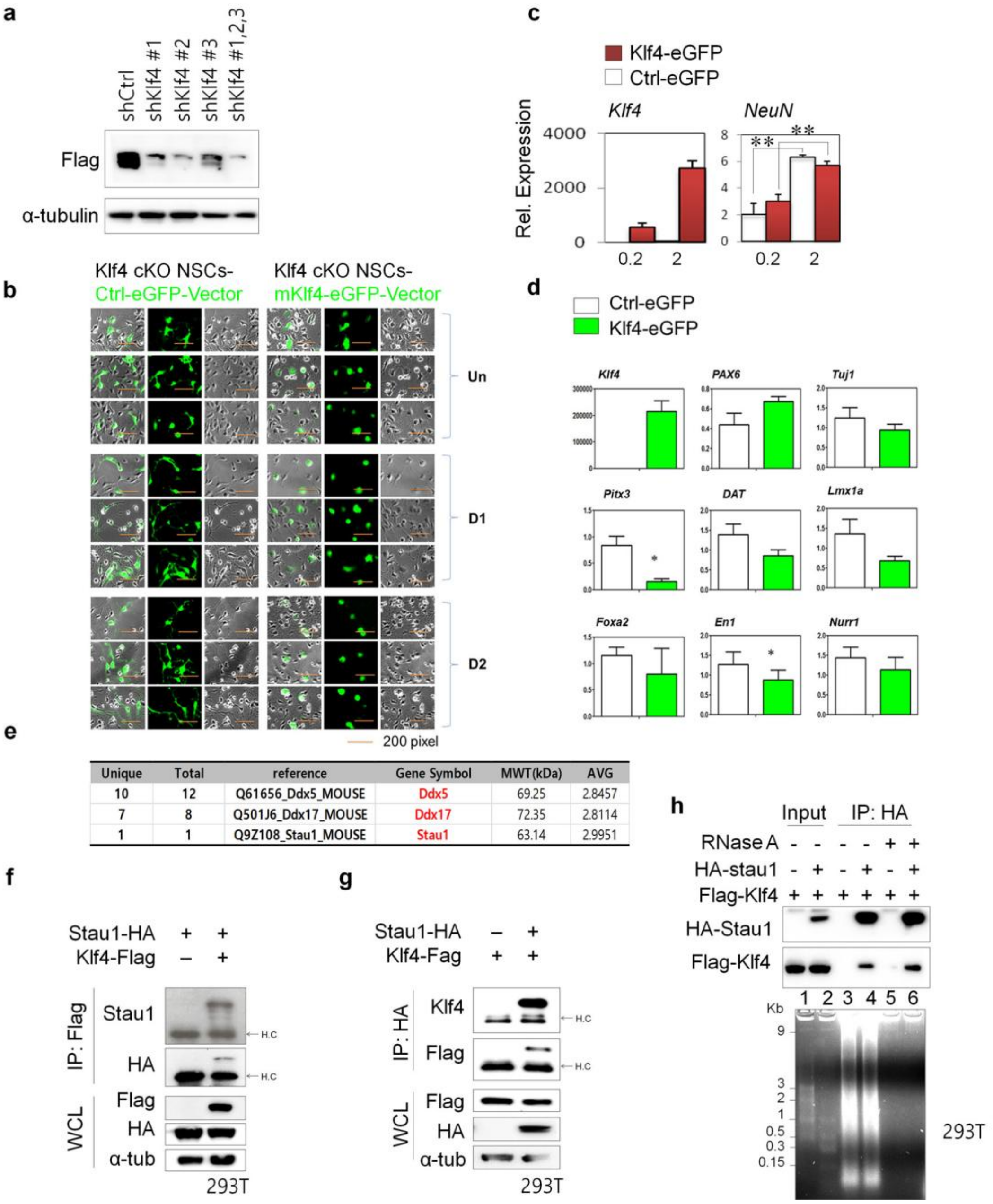

Supplementary Figure 7. Klf4 regulates NPC neuronal differentiation and interacts with post-transcriptional regulators. (**a**) NPCs expressing Flag-Klf4 were transfected with pLKO.1-

shScramble or pLKO.1-shKlf4 #1, #2, and #3, and one day later, Flag and α-tubulin in lysates were detected by immunoblotting (n=2). (**b**) *Klf4 cKO* NPCs transfected with *Control-EGFP* vector or *Klf4-EGFP* vector. GFP+ cells were assessed after 1 or 2 days of culture in N2 medium. (**c**, **d**) qPCR analysis of indicated transcripts in samples equivalent to those shown in (b) (n=3). (**e**) MS spectrometry analysis with Flag-Klf4 in NPCs (**f, g**) Co-Immunoprecipitation (co-IP) of HA-tagged Stau1 or Flag-tagged Klf4 in HEK293T cells transfected with these constructs. (**h**) (upper) Co-IP of above samples with or without RNase A (10µg/ml). Values correspond to mean±SD. *t*-tests were performed to calculate significance (*P < 0.05, **P < 0.005). (lower) Agarose gel of total RNA in above samples were sperated with standard electrophoresis procedures. We used a 7.5×10 cm mini-gel horizontal system (C.B.S. Scientific) to run a 1.5% agarose gel at 100 V (6 V/cm) for 2 h. Marker lanes (1–2) contained commercial RNA ladders (1. New England Bio Labs ssRNA ladder #No362s, 2. NEB low Range ssRNA ladder #No364s). Lane 3 and 4, total RNA in IP lysates without RNase A (10µg/ml). Lane 5 and 6, total RNA in IP lysates with RNase A (10µg/ml). RNA ladders and each total RNA samples were premixed with 2xRNA loading dye (Thermo Scientific, #Ro641). The bands were visualized using Molecular Imager® Gel Doc™ XR+ (Bio-Rad). See also Supplementary Figs. 19, 20.

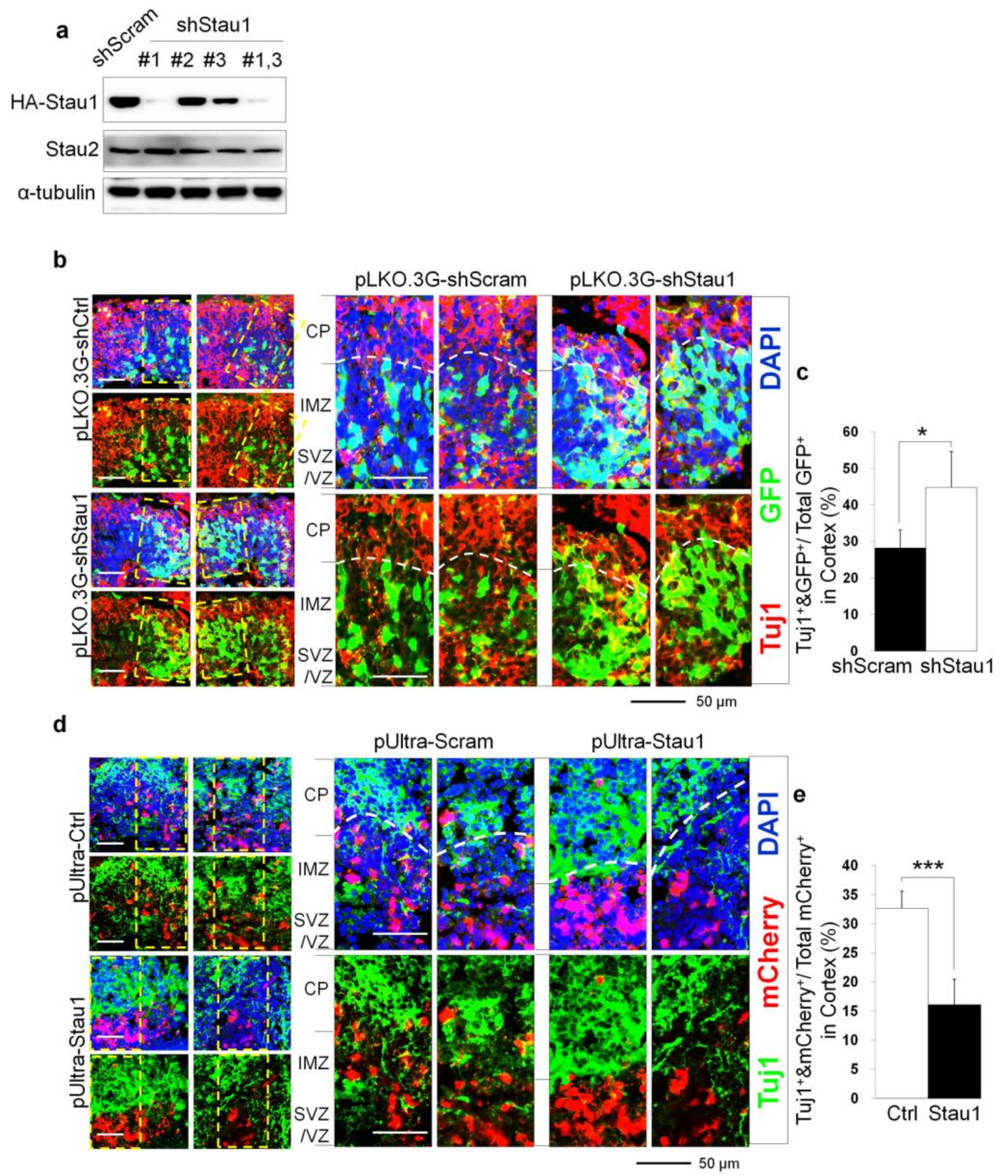

Supplementary Figure 8. Stau1 regulates NPC neuronal differentiation *in vivo*. (**a**) NPCs were infected with pLKO.1-shScramble or pLKO.1-shStau1 #1, #2, and #3, and one day later, Stau1, Stau2 and α-tubulin in lysates were detected by immunoblotting (n=2). (**b**) Effect of Stau1 knockdown on embryonic mouse cortex. Green fluorescent images indicate GFP labeled shScramble or shStau1 expression in electroporated embryo brain. CP, cortical plate; IMZ, intermediate zone; SVZ/VZ, (sub)ventricular zone. (**c**) The effect of Stau1 knockdown on

neurogenesis was evaluated by counting Tuj1 (red)/EGFP (green) double positive cells versus total EGFP-positive cells in cortex. shScramble (n=4), shStau1 (n=6). (**d**) Effect of Stau1 overexpression on embryonic mouse cortex. Red fluorescent images indicates mCherry labeled control or mStau1 expression in electroporated embryo brain. CP, cortical plate; IMZ, intermediate zone; SVZ/VZ, (sub)ventricular zone. (**e**) The effect of Stau1 overexpression on neurogenesis was evaluated by counting Tuj1(green)/mCherry(red) double positive cells versus total mCherry-positive cells in cortex. Control (n=5), Stau1 (n=7). Values correspond to mean±SD. *t*-tests were performed to calculate significance (*P < 0.05, **P < 0.005). See also Supplementary Fig. 20.

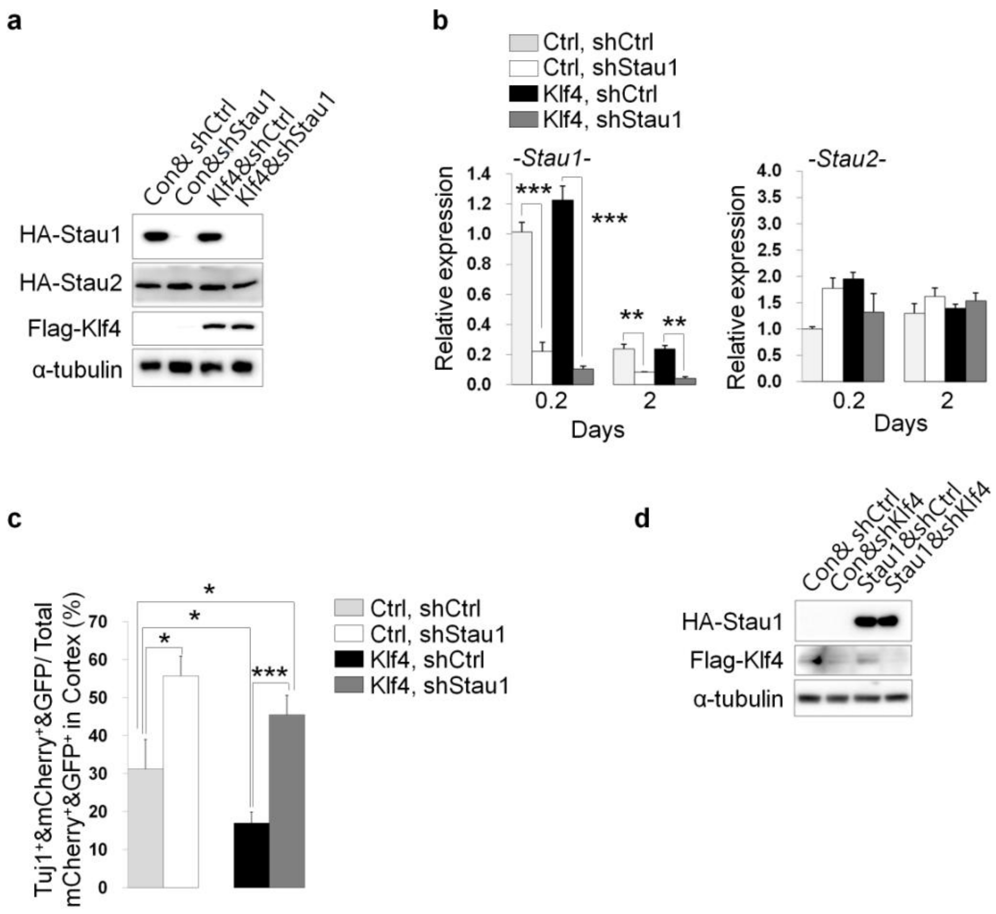

Supplementary Figure 9. Stau1 rescues defect of neurogenesis seen in Klf4 overexpression *in vivo*. (**a**) Immunoblot of indicated antibodies in Klf4-overexpressing or control NPCs infected with pLKO.1 shScramble or pLKO.1 shStau1 lentivirus (n=1-2). (**b**) RT-qPCR of indicated transcripts in samples shown in (a) (n=3). (**c**) The rescue effect of Stau1 knockdown on neurogenesis was evaluated by counting Tuj1$^+$mCherry$^+$GFP$^+$ triple positive cells versus total mCherry$^+$GFP$^+$ cells in electroporated cortex. shScramble/Control (n=3), shStau1/Control (n=3), shScramble/Klf4 (n=3), shStau1/Klf4 (n=6). (**d**) Immunoblots of indicated antibodies in Stau1-overexpressing or control NPCs infected with pLKO.1 shScramble or pLKO.1 shKlf4 lentivirus (n=1). Values correspond to mean±SD. Anova tests were performed to calculate significance (*P < 0.01, **P < 0.001, ***P < 0.0001). See also Supplementary Table 4, Supplementary Fig. 20.

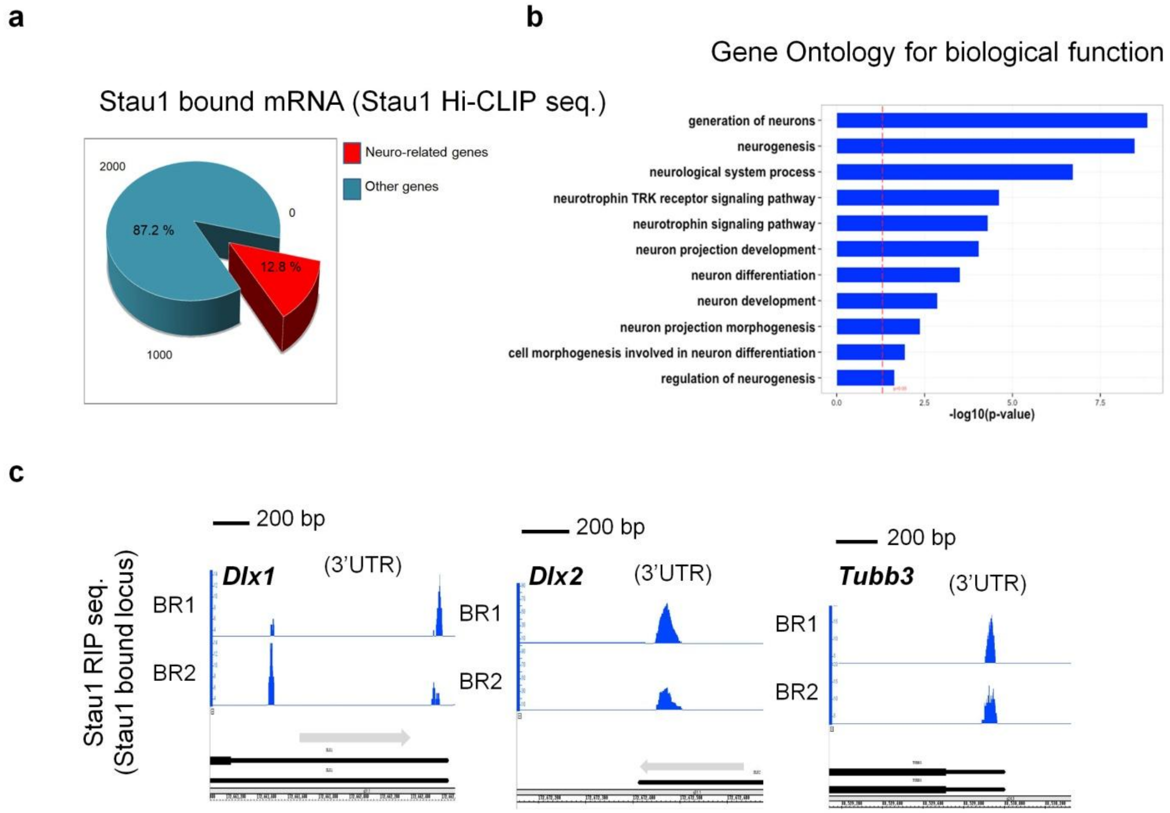

Supplementary Figure 10. Stau1 specifically binds to neurogenesis-associated mRNAs. (**a**) Stau1 hi-CLIP sequencing analysis in HEK293 cells (GSE52447)[1]. Among genes enriched by Stau1, 12.8% are neuro-related genes. (**b**) Gene ontology (GO) analysis of annotated genes at Staufen1 binding sites. (**c**) Stau1 RIP sequencing analysis[2]. Distribution of Stau1 binding peaks in HEK293 cells. Stau1 protein was highly enriched in 3'UTRs of *Dlx1*, *Dlx2* and *Tubb3* mRNAs.

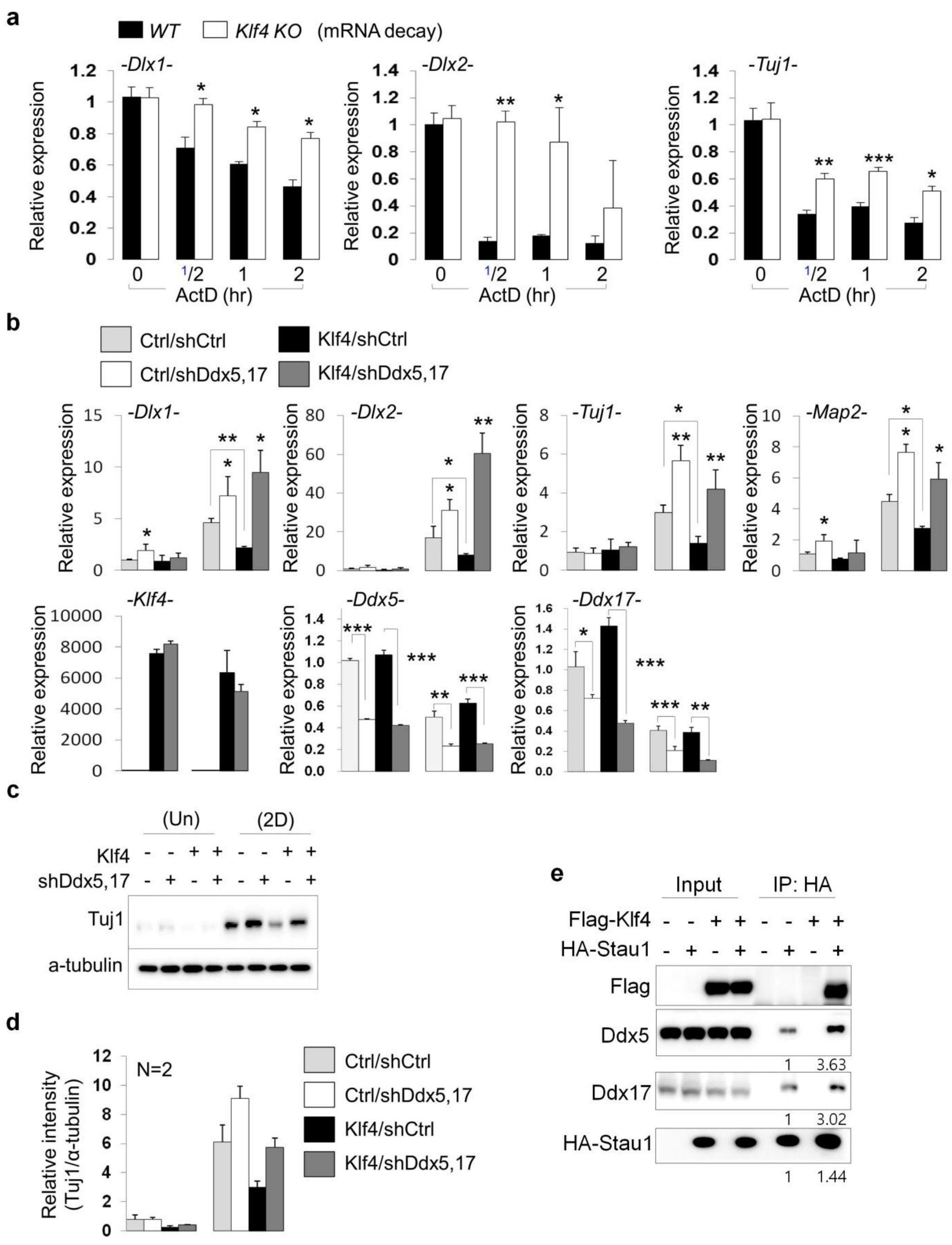

Supplementary Figure 11. Klf4 promotes mRNA decay and inhibit neurogenesis by cooperating with Stau1/Ddx5/17 complex. (**a**) Wild-type and Klf4 cKO NPCs were treated with actinomycin D (5 μg/ml) for indicated times and mRNAs were prepared for RNA stability assays (n=3). Values correspond to mean±SD. *t*-tests were performed to calculate significance

(*P < 0.05, **P < 0.005, ***P < 0.0005). (**b**) RT-qPCR of indicated transcripts in control- or Klf4-overexpressing NPCs infected with pLKO.1 shScramble or pLKO.1 shDdx5/17 lentivirus cultured for 0.2 or 2 days in differentiation conditions (n=3). Values correspond to mean±SD. Anova tests were performed to calculate significance (*P < 0.01, **P < 0.001, ***P < 0.0001). (**c**) Immunoblot of indicated antibodies in samples shown in (b) (n=2). See Supplementary Table 4. (**d**) Quantification of (c). (**e**) HEK293T cells were transfected with empty or Flag-Klf4 expression plasmids with control or HA-Stau1 vector. At 24 hours after transfection, lysates were immunoprecipitated with anti-IgG or anti-Mbd3 (n=1) and were analyzed by immunoblotting for indicated proteins. See also Supplementary Fig. 21.

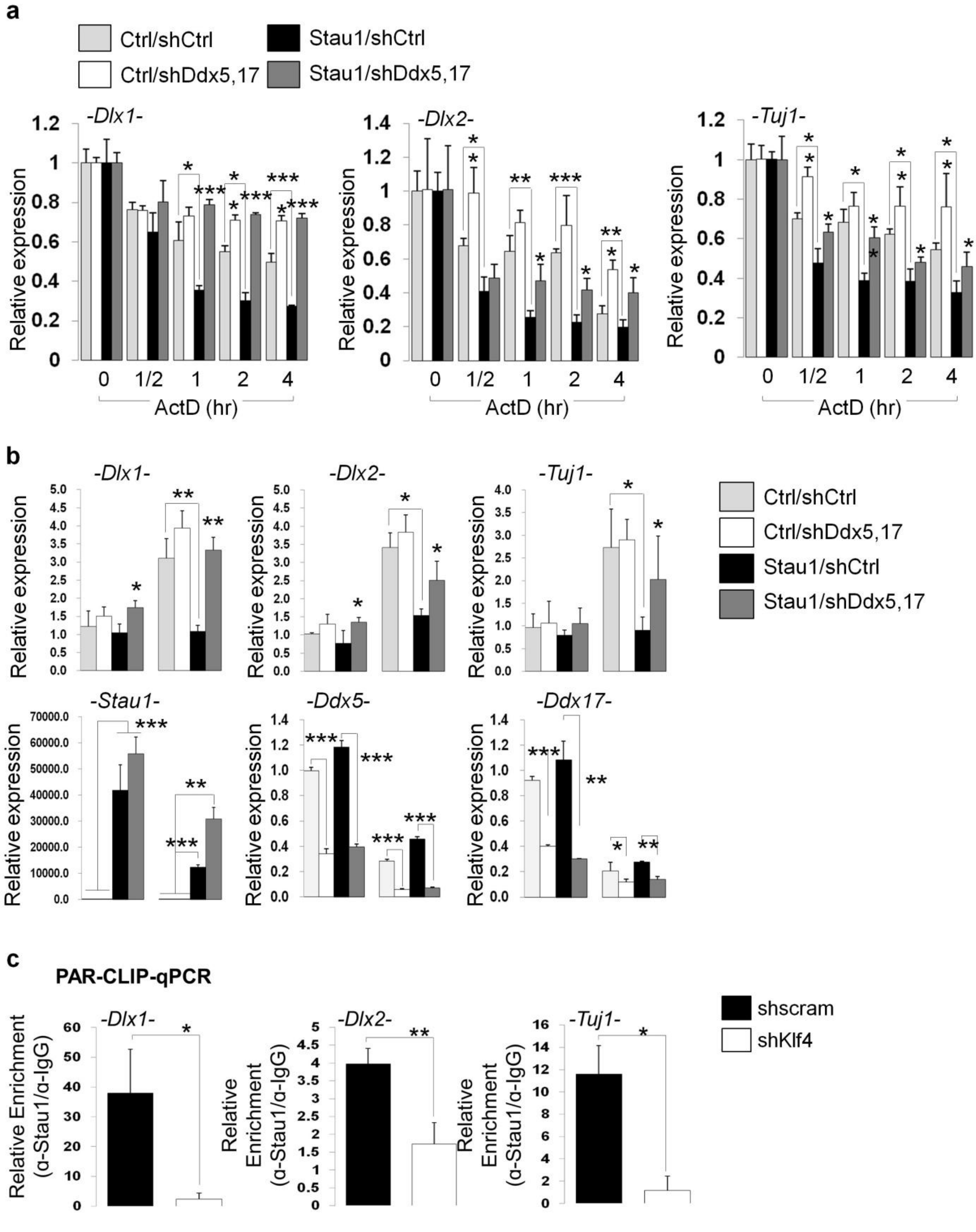

Supplementary Figure 12. Ddx5/17 double stranded RNA helicases are novel components

for SMD (**a**) RT-qPCR to assess the mRNA decay following Stau1 overexpression in NPCs transduced with shScramble or shDdx5/17 knockdown constructs. NPCs were treated with actinomycin D (5 μg/ml) for indicated times, and mRNAs were prepared for RNA stability assay (n=3). (**b**) RT-qPCR of indicated transcripts in control- or Stau1-overexpressing NPCs infected with pLKO.1 shScramble or pLKO.1 shDdx5/17 lentivirus cultured for 0.2 or 2 days in differentiation conditions (n=3). (**c**) PAR-CLIP qPCR to assess Stau1 enrichment on target mRNAs in NPCs transduced with shKlf4 knockdown or shScramble constructs. Each values enriched by Stau1 protein were divided by IgG control. Data is presented as mean±SD. Anova tests were performed to calculate statistical significance (*P < 0.01, **P < 0.001, ***P < 0.0001). See Supplementary Table 4.

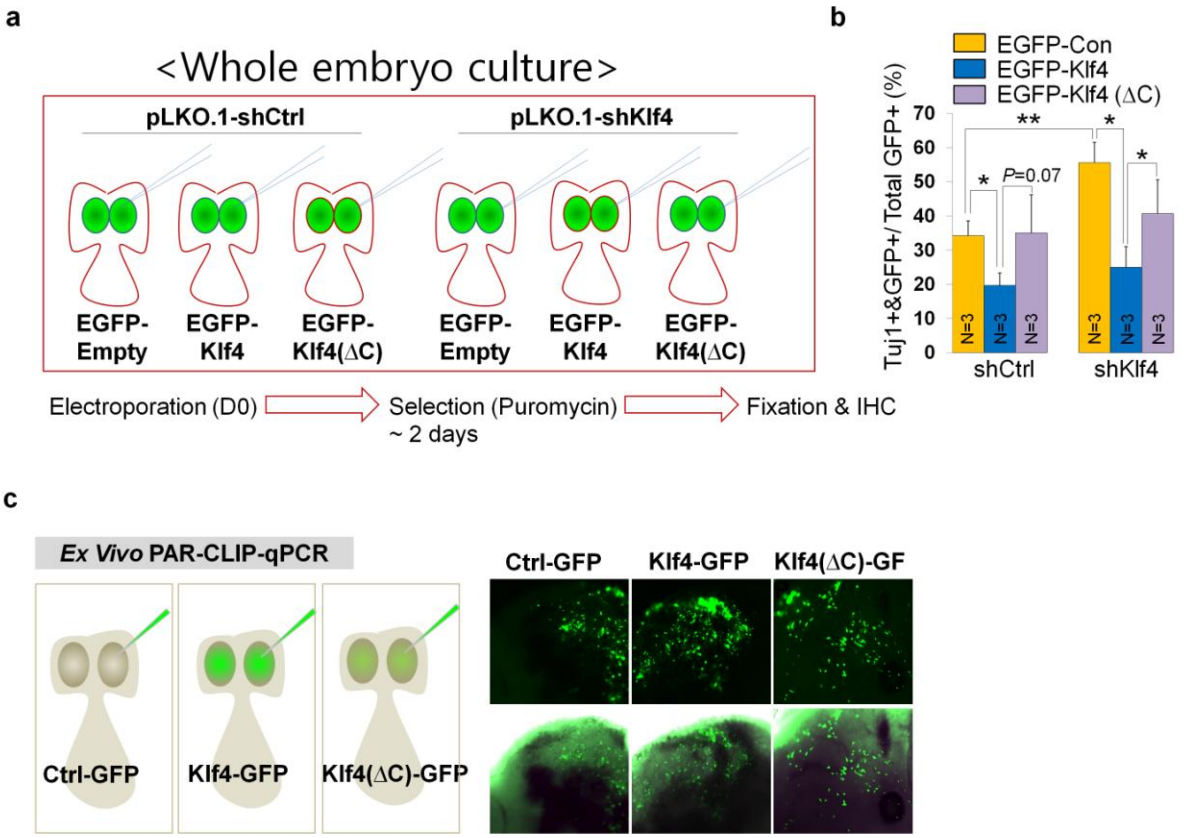

Supplementary Figure 13. Klf4 inhibits neurogenesis through formation of Klf4/Stau1 complex *ex vivo*. (**a**) Schematic of injection site (green) and experimental process for *ex utero* electroporation. pEGFP-Control, pEGFP-Klf4, or pEGFP-ΔC vectors were electroporated in mouse embryonic cortex together with shscramble or shKlf4 lentiviral vectors. (**b**) The effect of wild-type Klf4 or its mutant (ΔC) overexpression on neurogenesis was evaluated by counting Tuj1/EGFP double positive cells versus total EGFP-positive cells in VZ/SVZ/IMZ/CP compartments (n=3). (**c**) Schematic of PAR-CLIP-qPCR experiment in cortices electroporated with pEGFP-Control, pEGFP-Klf4, or pEGFP-ΔC constructs and grown in *ex vivo* condition for 2 days (2D).

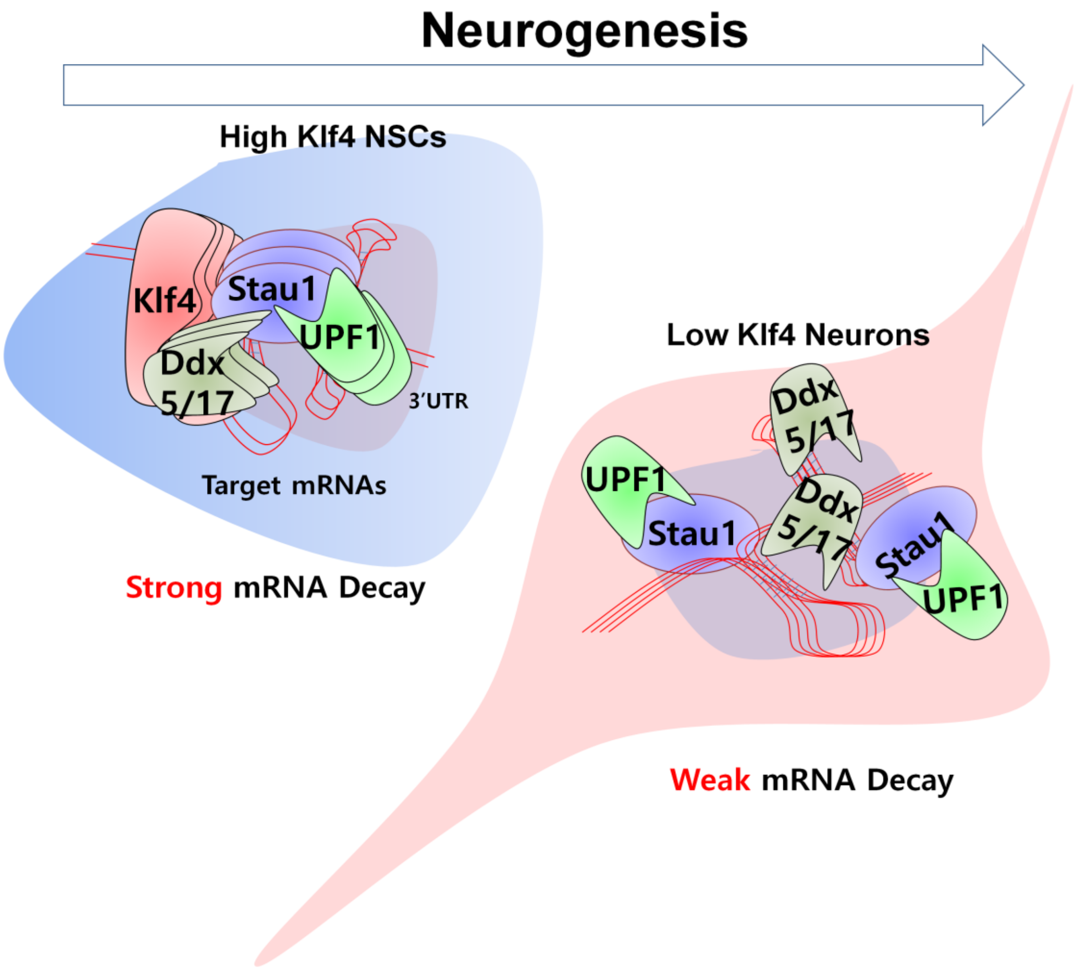

Supplementary Figure 14. Klf4/Ddx5/17 dependent Stau1 mediated mRNA decay (SMD) regulates cortical neurogenesis. A model of proposed function of the Klf4-Ddx5/17-Stau1 axis in NPC cell fate determination during neurogenesis.

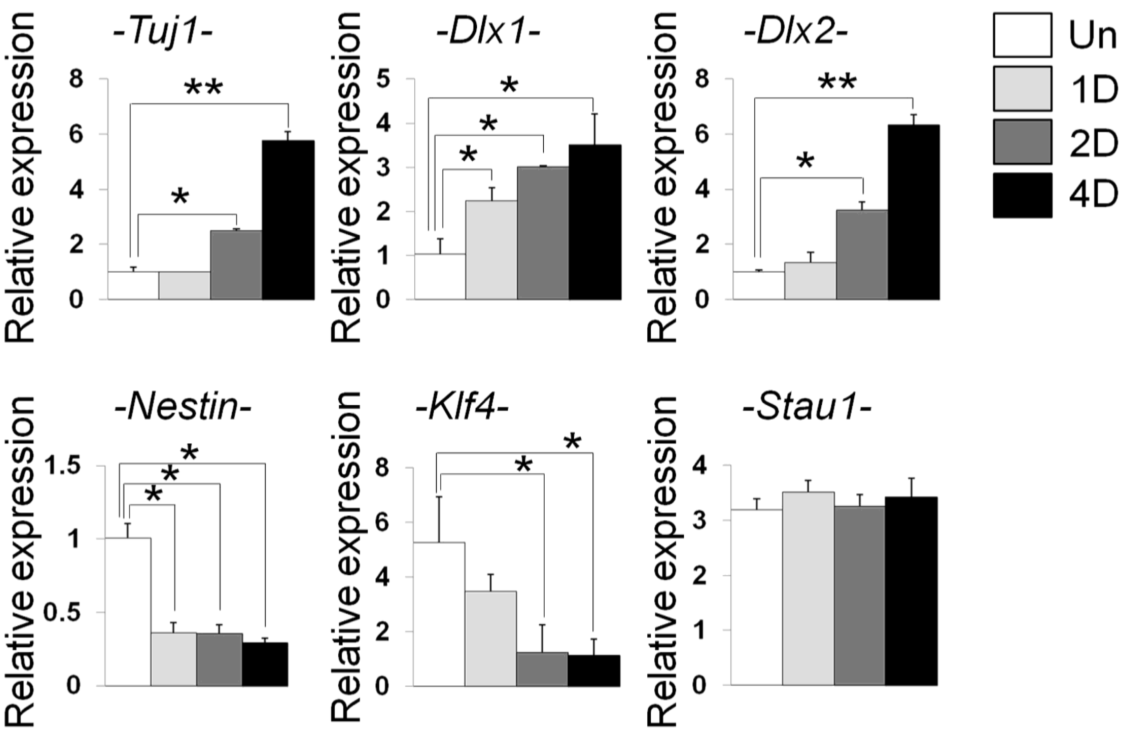

Supplementary Figure 15. *Klf4*, but not *Stau1*, decreases during neural differentiation of NPCs. qPCR analysis of indicated mRNAs. Values correspond to the mean±SD. Diff. (d), days in differentiation. Statistical t-test analysis was performed to calculate significance (*P < 0.05, **P < 0.005, ns, P > 0.05).

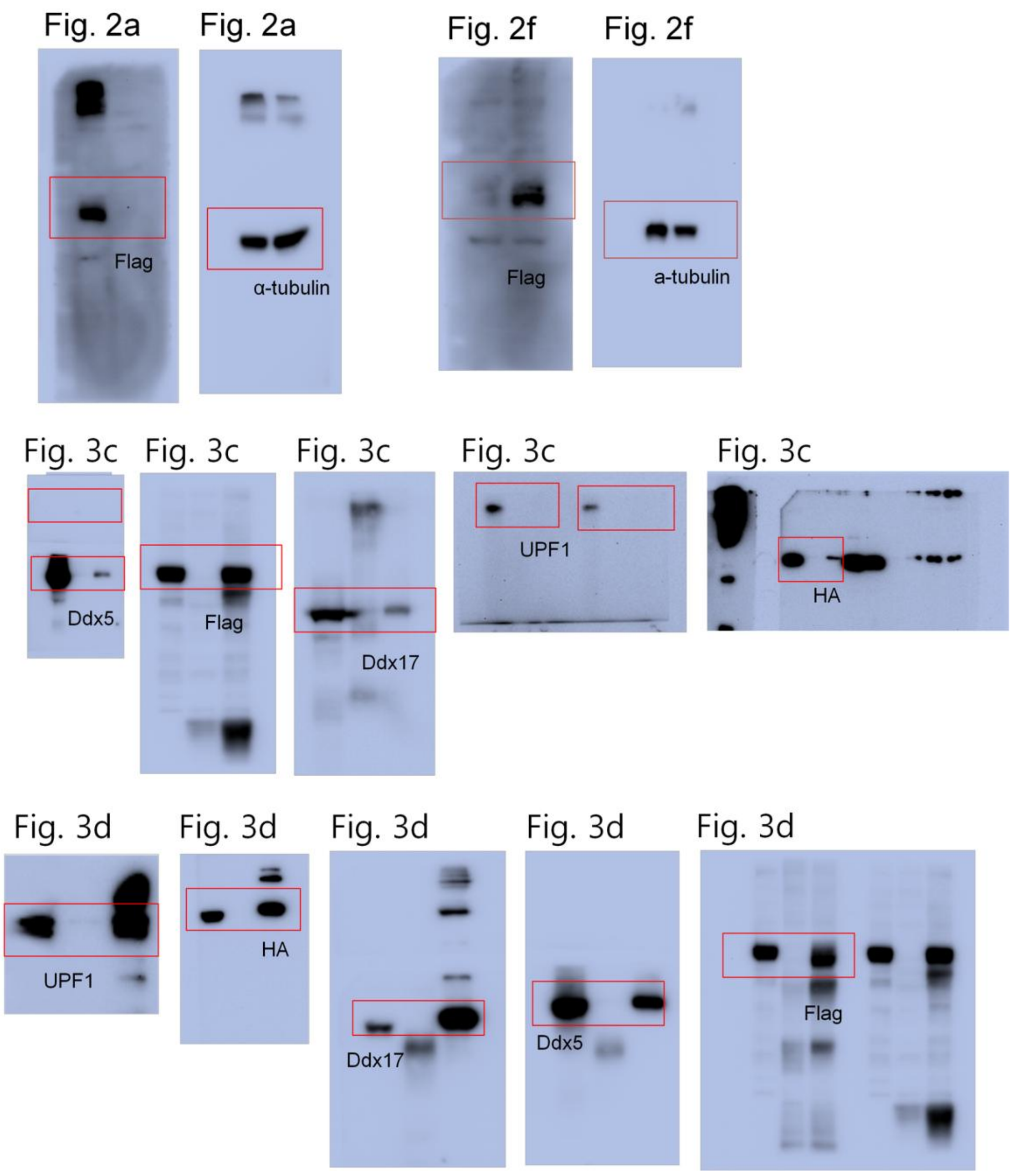

Supplementary Figure 16. Full blots from main figures. The full blots used for Figure 2a, f, 3c, d. The specific bands shown in the main figures are indicated by red boxes.

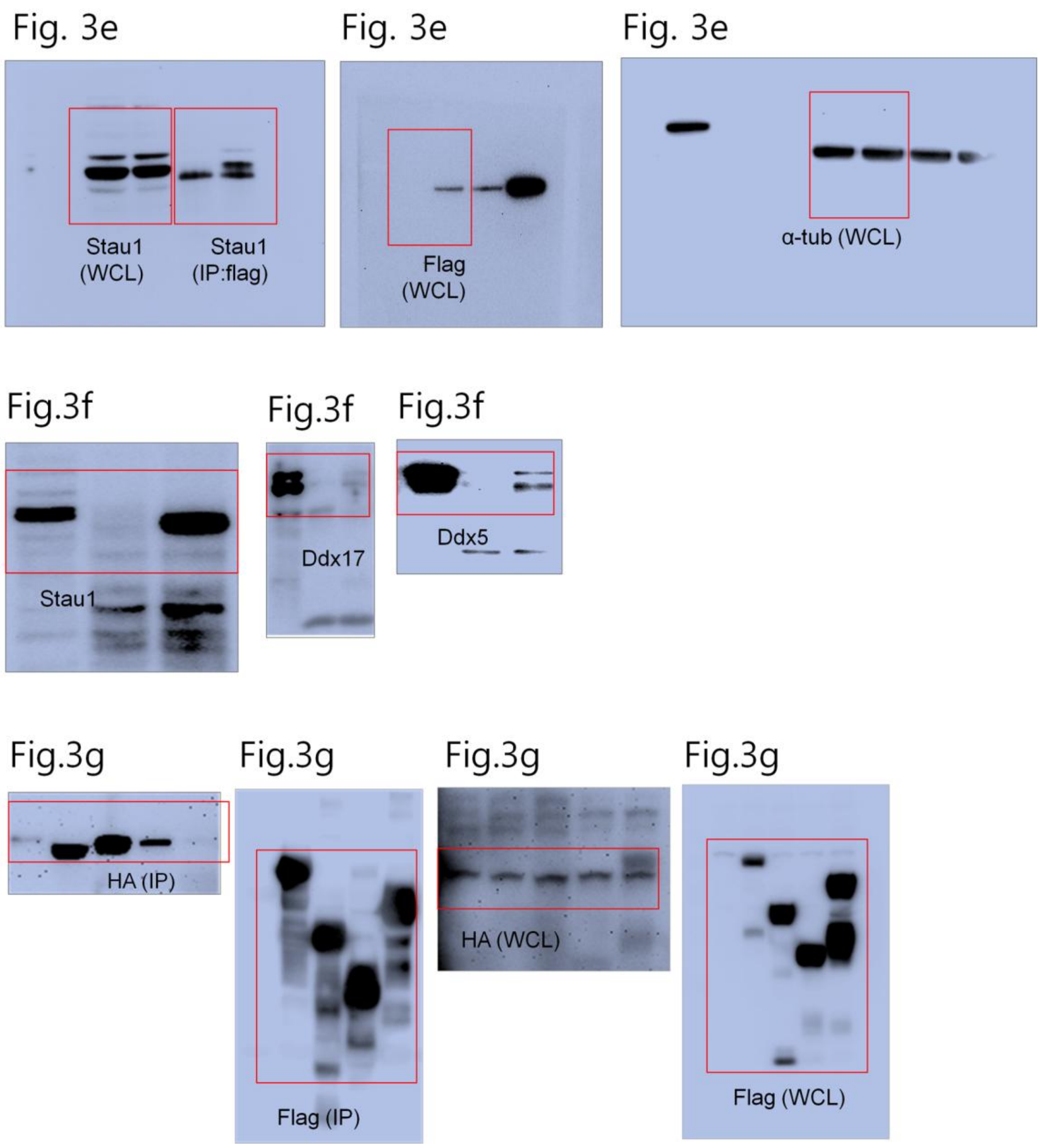

Supplementary Figure 17. Full blots from main figures. The full blots used for Figure 3e-g.

The specific bands shown in the main figures are indicated by red boxes.

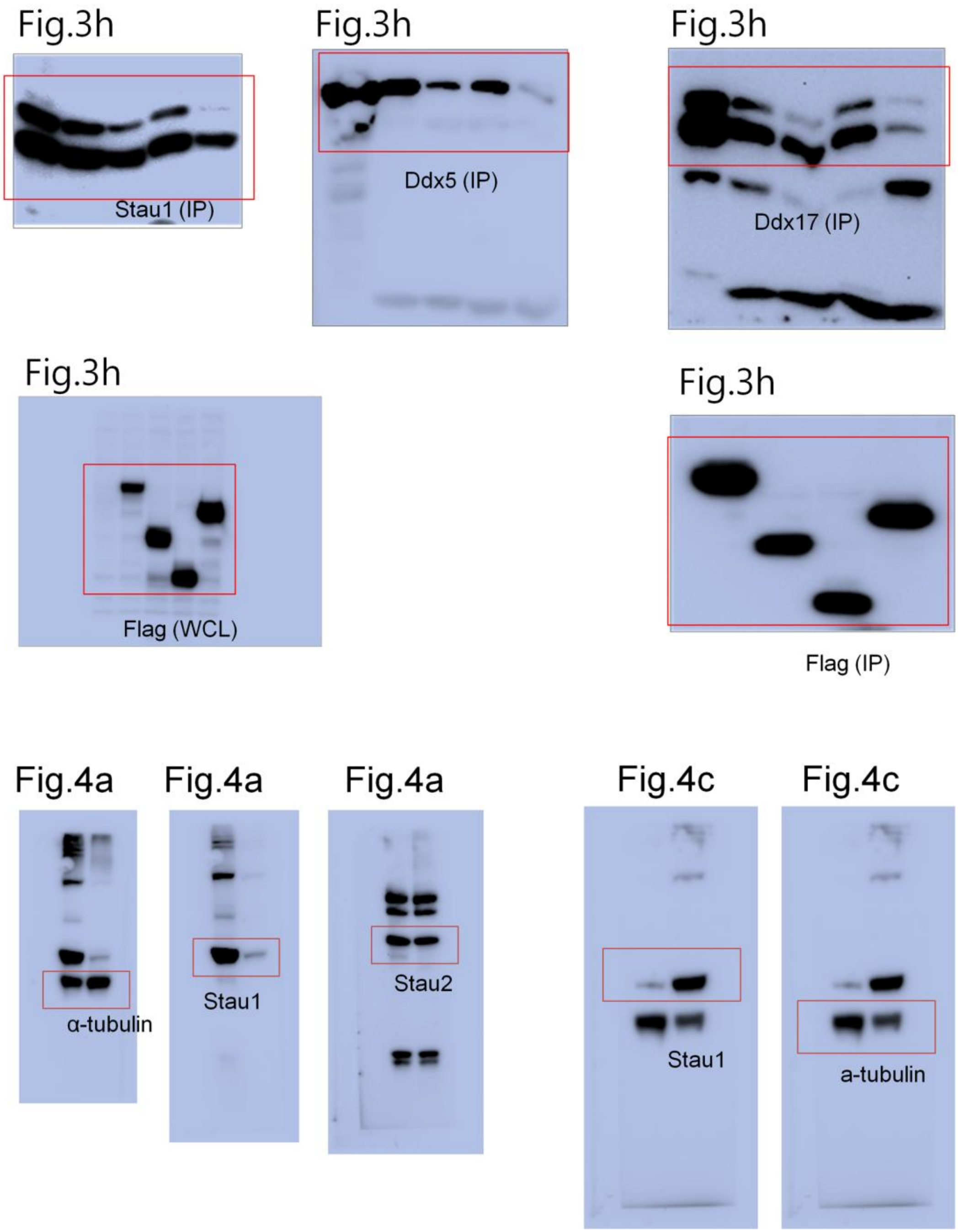

Supplementary Figure 18. Full blots from main figures. The full blots used for Figure 3h, 4a, c. The specific bands shown in the main figures are indicated by red boxes.

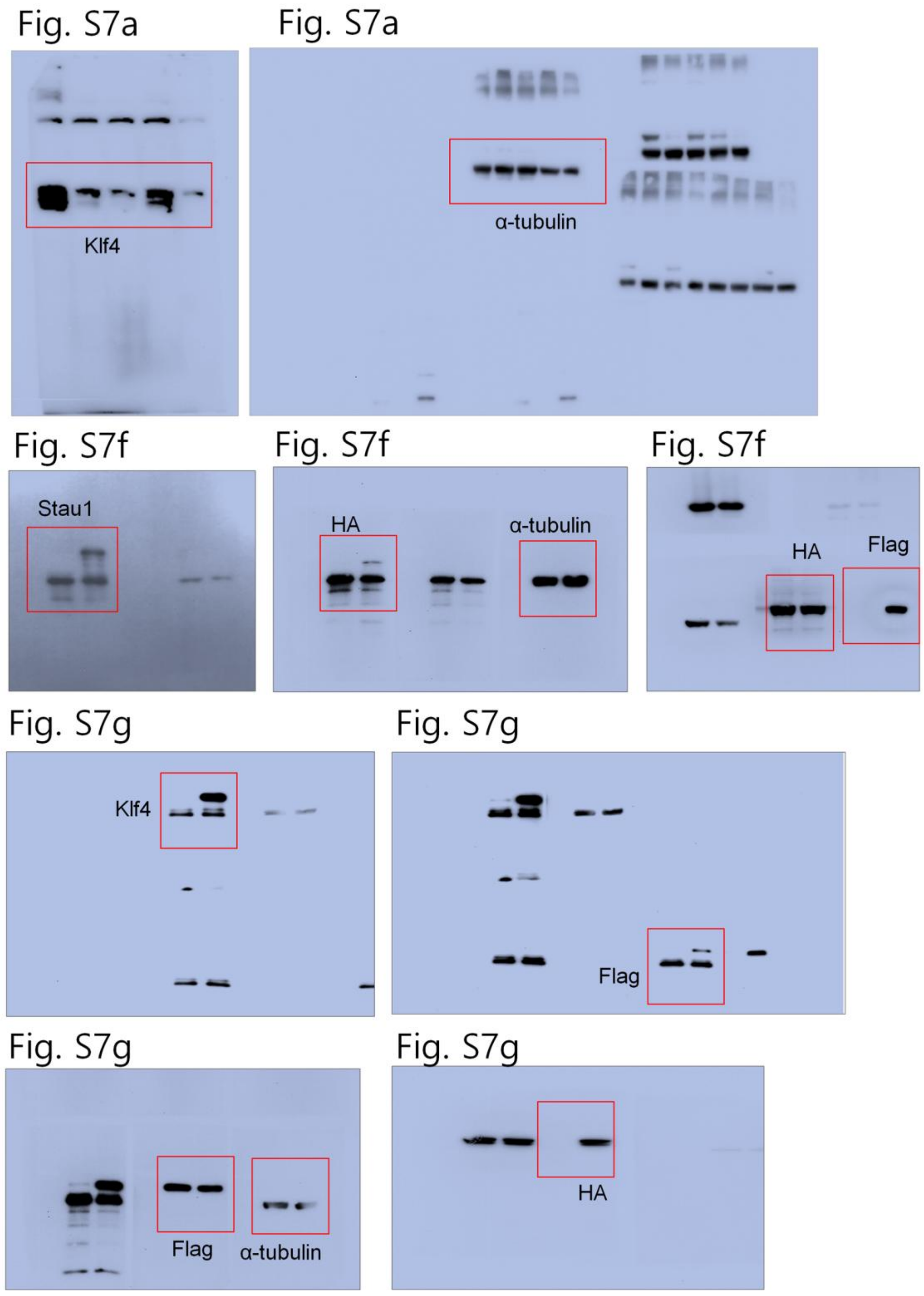

Supplementary Figure 19. Full blots from main figures. The full blots used for Supplementary Figure 7a, f, g. The specific bands shown in the main figures are indicated by red boxes.

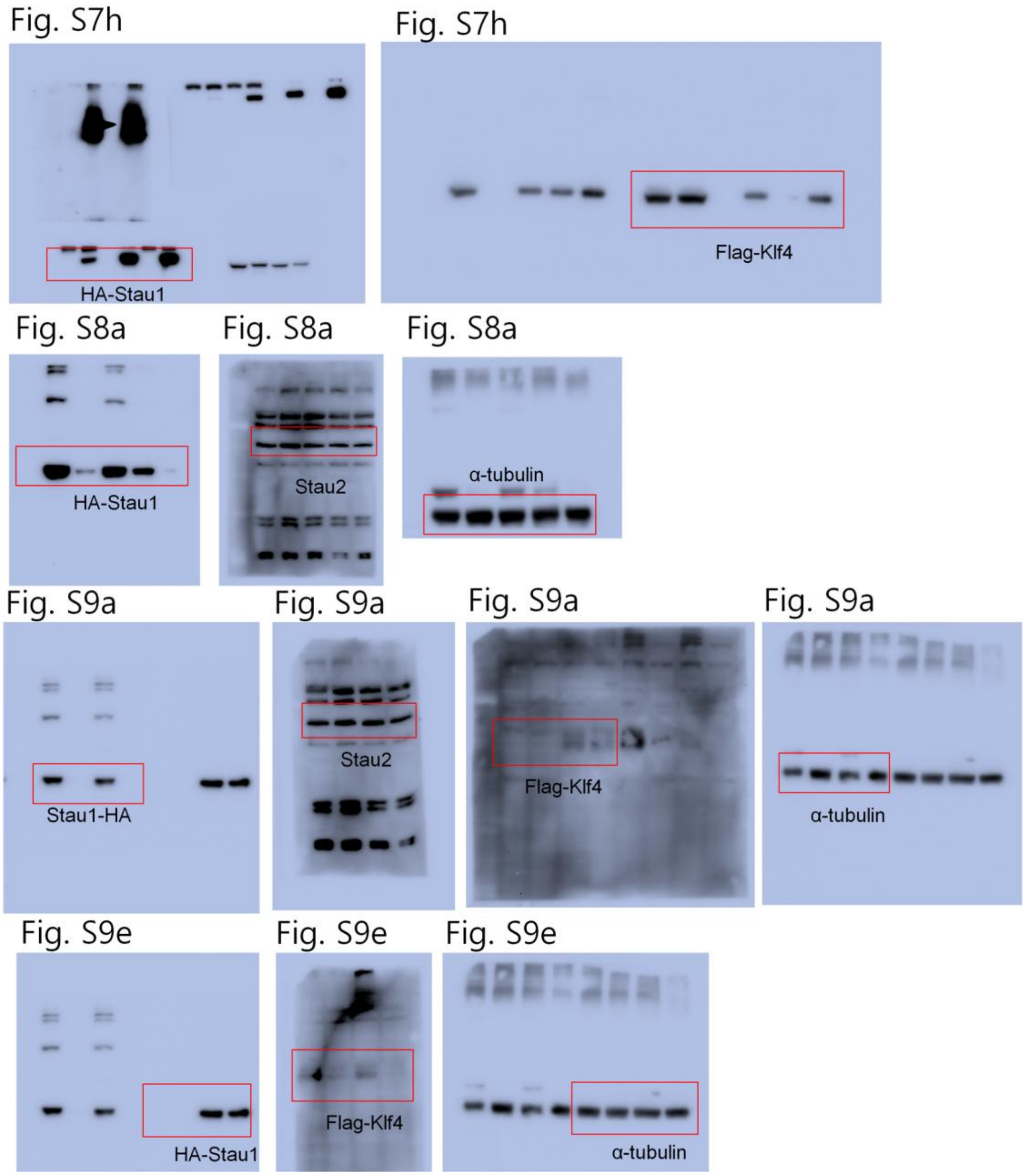

Supplementary Figure 20. Full blots from main figures. The full blots used for Supplementary Figure 7h, 8a, 9a, e. The specific bands shown in the main figures are indicated by red boxes.

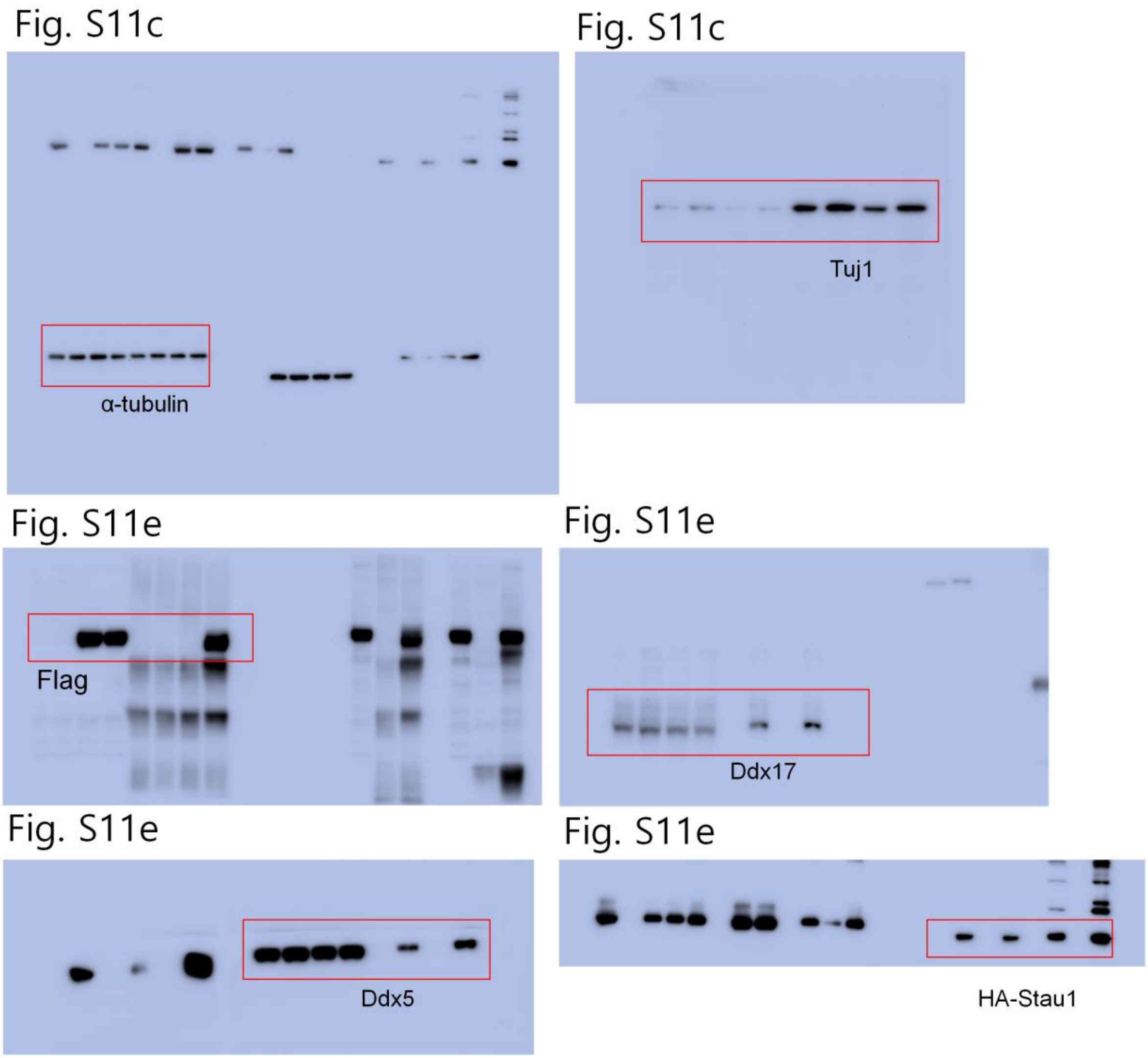

Supplementary Figure 21. Full blots from main figures. The full blots used for Supplementary Figure 11c, e. The specific bands shown in the main figures are indicated by red boxes.

Supplementary Table 1. Construction of pLKO3G shRNA and target sequences.

To knock down Klf4 and Stau1 expression in NSCs we generated pLKO.3G short hairpin (sh)RNA lenti-viral vector system. Target sequences of Klf4 and Stau1 were chosen using RNAi consortium shRNA library (http://www.broadinstitute.org/rnai/public/) (three clones targeting different sequences in the three coding regions of Klf4 and Stau1 gene) against Klf4 and Stau1. To generate oligos for cloning, sense and antisense sequences of chosen target sequences were ordered from IDT. Sequence-verified shRNA lentiviral plasmid vectors for mouse Klf4 and Stau1 gene were cloned into the pLKO.3G vector and knockdown efficiency were measured by western blot (see Supplementary Fig. 7a).

For gene transfections we utilized Lipofectamine 3000 and Amaxa nucleofection (Lonza, http: //www.lonzabio.com/cell-biology/transfection/) for the transfection of mouse NSCs. A range of knockdown efficiencies was observed. The shKlf4 (#1, #2 and #3) clones showed maximum efficiencies and three vectors were used for further experiments (see Fig. 2a). For shStau1 knockdown experiment, Two (we encoded it as shStau1 #1 and #3) out of three clones appeared to be efficiently knocking down Stau1 expression in NSCs (see Fig. 4a and Supplementary Fig. 8a) and both were used for further experiments. For shControl transfection, sequences were chosen based on Sigma-Aldrich shRNA products which had been previously validated as a non-target shRNA control. The plasmids containing the below sequences that does not target any gene, making it useful as a negative control in our experiments and appear no change Klf4 or Stau1 expression (see Supplementary Fig. 7a and 8a).

Gene	shKlf4 #1	Target region
Target sequence<br>Ordered oligo	CATGTTCTAACAGCCTAAATG<br>AATTCATGTTCTAACAGCCTAAATGCTCGAGCATTTAGGCTGTTAGAACATGTTTTTTTAT	3’ UTR
Gene	shKlf4 #2	
Target sequence<br>Ordered oligo	TTGTGGATATCAGGGTATAAA<br>AATTTTGTGGATATCAGGGTATAAACTCGAGTTTATACCCTGATATCCACAATTTTTTTAT	3’ UTR
Gene	shKlf4 #3	
Target sequence<br>Ordered oligo	CTCTCTCACATGAAGCGACTT<br>AATTCTCTCTCACATGAAGCGACTTCTCGAGAAGTCGCTTCATGTGAGAGAGTTTTTTTAT	CDS
Gene	shStau1 #1	
Target sequence<br>Ordered oligo	ATTGCGCTGAAGCGGAATTTG<br>AATTATTGCGCTGAAGCGGAATTTGCTCGAGCAAATTCCGCTTCAGCGCAATTTTTTTTAT	CDS
Gene	shStau1 #2	
Target sequence<br>Ordered oligo	GCCAAGGGATGAATCCTATTA<br>AATTGCCAAGGGATGAATCCTATTACTCGAGTAATAGGATTCATCCCTTGGCTTTTTTTAT	CDS
Gene	shStau1 #3	
Target sequence<br>Ordered oligo	CCAGGGATTCCAGGTTGAATA<br>AATTCCAGGGATTCCAGGTTGAATACTCGAGTATTCAACCTGGAATCCCTGGTTTTTTTAT	CDS

Supplementary Table 2. Primer sequences used for quantitative or RT-PCR analysis.

Genes	Primer Sequences	Tm (℃)
Nestin	5'-CCCTGAAGTCGAGGAGCTG-3'	57.4
	5'-CTGCTGCACCTCTAAGCGA-3'	57.3
Dlx1	5'-ATGCCAGAAAGTCTCAACAGC-3'	60.6
	5'-AACAGTGCATGGAGTAGTGCC-3'	62.4
Dlx2	5'-AAAGAAAGTCCGGAAACCACG-3'	60.1
	5'-TCTTCTTGAACTTGCATCGGC-3'	60.5
Tuj1	5'-TAGACCCCAGCGGCAACTAT-3'	58.2
	5'-GTTCCAGGTTCCAAGTCCACC-3'	58.0
Gad67	5'-GCCACAAACTCAGCGGCATAGAAA-3'	60.0
	5'-AGACGTCATACTGCTTGTCTGGCT-3'	60.0
NeuN	5'-GAAACCGCAAGCCCTCATTTC-3'	60.1
	5'-TTGGATGCCTCTTGGTTTGGT-3'	60
Pitx3	5'-TGCGCTGTCGTTATCGGAC-3'	62.4
	5'-GGTAGCGATTCCTCTGGAAGG-3'	61.7
Pax6	5'-AACCCACGCAAGATGGCTG-3'	62.9
	5'-GCATCCCAGTGCATAAAAACCA-3'	61.4
Dat	5'-AAATGCTCCGTGGGACCAATG-3'	63
	5'-GTCTCCCGCTCTTGAACCTC-3'	61.9
Lmx1a	5'-ACGGCCTGAAGATGGAGGA-3'	62.3
	5'-CAGAAACCTGTCCGAGATGAC-3'	60.1
Foxa2	5'-CCCTACGCCAACATGAACTCG-3'	63
	5'-GTTCTGCCGGTAGAAAGGGA-3'	61.2
En1	5'-CTAAGGCCCGATTTCGGTTG-3'	60.8
	5'-GAGTGAACGGGGTCTCTACCT-3'	62.4
Klf4	5'-GTGCCCCGACTAACCGTTG-3'	63
	5'-GTCGTTGAACTCCTCGGTCT-3'	61.2
Staufen1	5'-GGACCCTCACTCTCGGATG-3'	60.8
	5'-TTCTGGCAGGGGTTCACTCT-3'	62.7
Staufen2	*5'-AGTGACCTCTGGCACAACTCT-3'*	62.8
	5'-TGGCTTCAGCAGTAGGAGATG-3'	61.3
Gapdh	5'-AGGTCGGTGTGAACGGATTTG-3'	57.6
	5'-TGTAGACCATGTAGTTGAGGTCA-3'	55.1

Gapdh : Glyceeraldehyde-3-phosphate dehydrogenase

Supplementary Table 3. Primer sequences used for mRNA decay and PAR-CLIP qPCR analysis.

Gene	Sequence	Tm (°C)	GC (%)
mTuj1-CLIP(1-F)	AGTTGCTCGCAGCTGGGGTGT	58.2	55
mTuj1-CLIP(1-R)	AAAGCTGGGGGCAGTGTCA	59.7	57.9
mTuj1-CLIP(2-F)	CTTCTCACCAGCTCATTAGG	52.5	50
mTuj1-CLIP(2-R)	ACAGAGGTGGCTAAAATGGG	54.9	50
mTuj1-CLIP(3-F)	CCCATTTTAGCCACCTCTGT	54.9	50
mTuj1-CLIP(3-R)	CTGACAGACAGCAGTAAC	50	50
mDlx1-CLIP(1-F)	GGTTATCAGTTCGCACTCAC	53.4	50
mDlx1-CLIP(1-R)	AAGCCCTCTGCCTTCACTGTT	54	52
mDlx1-CLIP(2-F)	TGCTTCTCGGAGCCCCTAAAA	58.6	52.4
mDlx1-CLIP(2-R)	AAGATGAGAGTCTGGTCCGG	55.7	55
mDlx1-CLIP(3-F)	CCGGACCAGACTCTCATCTT	56	55
mDlx1-CLIP(3-R)	GGTCGTAGAATGAGGACAGC	55	55
mDlx1-CLIP(4-F)	CTCCAAAAAGTCACCAGGTCTG	55.7	50
mDlx1-CLIP(4-R)	TCAGCGTCAGATGAAGTCTG	54.3	50
mDlx1-CLIP(5-F)	CAGACTTCATCTGACGCTGA	54.3	50
mDlx1-CLIP(5-R)	ACTTTCCTGTCCTTGTTCCC	54.7	50
mDlx1-CLIP(6-F)	GGAACAAGGACAGGAAAG	50.3	50
mDlx1-CLIP(6-R)	CCAGGTGTTCTTGTCCAAACG	56.4	52.4
mDlx1-CLIP(7-F)	GAACACCTGGCCAAGAAG	53.5	55.6
mDlx1-CLIP(7-R)	GCCGCTGCTTGTGTCTTACT	58	55
mDlx1-CLIP(8-F)	TCGACCACAGAACACAAGTC	54.7	50
mDlx1-CLIP(8-R)	TCGGGTTTACAGCCACACAC	57.7	55
mDlx1-CLIP(9-F)	GTGTGTGGCTGTAAACCCGA	57.7	55
mDlx1-CLIP(9-R)	CTTATTTACAAAGTTCGCCCCC	54.3	45.5
mDlx2-CLIP(1-F)	TCGAGTGGACAGCGTCTGA	57.9	58.2
mDlx2-CLIP(1-R)	AGAAGTGGCTCCACGAC	54.5	58.8
mDlx2-CLIP(2-F)	GTCGTGGAGCCACTTCT	54.5	58.8
mDlx2-CLIP(2-R)	ACCCTGAGGATCACGCTGA	58.3	57.9
mDlx2-CLIP(3-F)	TCAGCGTGATCCTCAGGGT	58.3	57.9
mDlx2-CLIP(3-R)	TGGAGTAGGACCCAGGAG	55.3	61.1
mDlx2-CLIP(4-F)	ACCTCCTGGGTCCTACTCCA	59.6	60
mDlx2-CLIP(4-R)	GGTCATCCGCAAAGGCACCTA	59.8	57.1
mDlx2-CLIP(5-F)	TAGGTGCCTTTGCGGATGACC	59.8	57.1
mDlx2-CLIP(5-R)	GGGGAAATCTGCACAGACACC	53.6	52.6
mDlx2-CLIP(6-F)	GGTGTCTGTGCAGATTTCCCC	58.3	57.1

mDlx2-CLIP(6-R)	ATAGGGACTGCTGAGGTCACTG	58.2	54.5

m*Tuj1*-CLIP

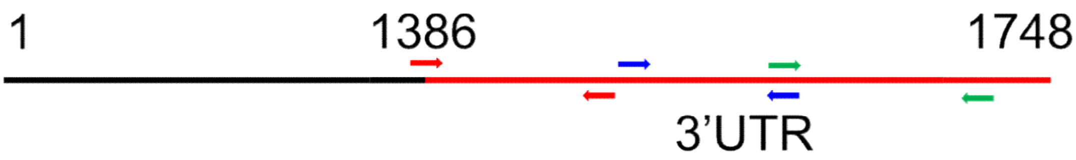

m*Dlx1*-CLIP

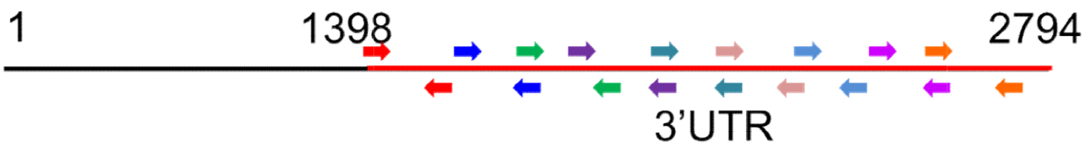

m*Dlx2*-CLIP

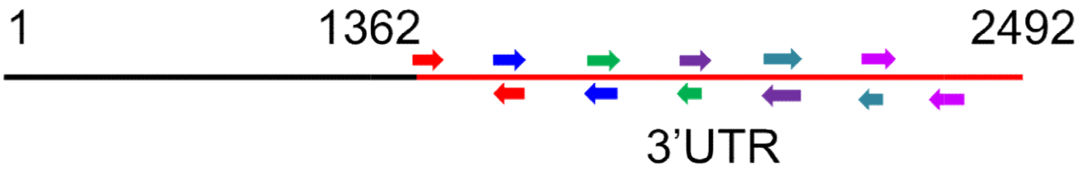

Supplementary Table 4. Values and parameters for the statistical comparisons shown in main and supplementary figures

Fig.1F

Klf4 gene					
Table Analyzed	Data 1				
One-way analysis of variance					
P value	< 0.0001				
P value summary	***				
Are means signif. different	Yes				
Number of groups	4				
F	39.81				
R squared	0.9372				
ANOVA Table	SS	df	MS		
Treatment (between colum	45.17	3	15.06		
Residual (within columns	3.026	8	0.3783		
Total	48.2	11			
Tukey's Multiple Comparis	Mean Diff.	q	Significant?	Summary	95% CI of diff
WT-Un vs cKO-Un	4.106	11.56	Yes	***	2.498 to 5.714
WT-Un vs WT-2DIV	0.7079	1.994	No	ns	-0.9003 to 2.316
WT-Un vs cKO-2DIV	4.294	12.09	Yes	***	2.685 to 5.902
cKO-Un vs WT-2DIV	-3.398	9.569	Yes	***	-5.006 to -1.790
cKO-Un vs cKO-2DIV	0.1878	0.529	No	ns	-1.420 to 1.796
WT-2DIV vs cKO-2DIV	3.586	10.1	Yes	***	1.978 to 5.194

Dlx1 gene					
Table Analyzed	Data 1				
One-way analysis of variance					
P value	0.0002				
P value summary	***				
Are means signif. differe	Yes				
Number of groups	4				
F	24.67				
R squared	0.9024				
ANOVA Table	SS	df	MS		
Treatment (between colu	4184	3	1395		
Residual (within columns	452.3	8	56.53		
Total	4636	11			
Tukey's Multiple Compari	Mean Diff.	q	Significant?	Summary	95% CI of diff
WT-Un vs cKO-Un	-12.75	2.936	No	ns	-32.41 to 6.913
WT-Un vs WT-2DIV	-8.856	2.04	No	*	-28.52 to 10.80
WT-Un vs cKO-2DIV	-48.98	11.28	Yes	***	-68.64 to -29.32
cKO-Un vs WT-2DIV	3.891	0.8963	No	ns	-15.77 to 23.55
cKO-Un vs cKO-2DIV	-36.23	8.347	Yes	**	-55.89 to -16.57
WT-2DIV vs cKO-2DIV	-40.12	9.243	Yes	***	-59.79 to -20.46

Dlx2 gene					
Table Analyzed	Data 1				
One-way analysis of variance					
P value	< 0.0001				
P value summary	***				
Are means signif. diffe	Yes				
Number of groups	4				
F	130.1				
R squared	0.9799				
ANOVA Table	SS	df	MS		
Treatment (between c	42130	3	14040		
Residual (within colum	863.7	8	108		
Total	42990	11			
Tukey's Multiple Comp	Mean Diff.	q	Significant?	Summary	95% CI of diff
WT-Un vs cKO-Un	-3.892	0.6487	No	ns	-31.06 to 23.28
WT-Un vs WT-2DIV	-70.07	11.68	Yes	***	-97.24 to -42.90
WT-Un vs cKO-2DIV	-145.4	24.24	Yes	***	-172.6 to -118.3
cKO-Un vs WT-2DIV	-66.18	11.03	Yes	***	-93.35 to -39.01
cKO-Un vs cKO-2DIV	-141.5	23.59	Yes	***	-168.7 to -114.4
WT-2DIV vs cKO-2DI	-75.37	12.56	Yes	***	-102.5 to -48.20

Tuj1					
Table Analyzed	Data 1				
One-way analysis of variance					
P value	< 0.0001				
P value summary	***				
Are means signif. different	Yes				
Number of groups	4				
F	56.16				
R squared	0.9547				
ANOVA Table	SS	df	MS		
Treatment (between colum	12.43	3	4.142		
Residual (within columns	0.59	8	0.07375		
Total	13.02	11			
Tukey's Multiple Comparis	Mean Diff.	q	Significant?	Summary	95% CI of diff
WT-Un vs cKO-Un	-0.2157	1.375	No	ns	-0.9258 to 0.4945
WT-Un vs WT-2DIV	-0.6959	4.433	No	*	-1.406 to 0.01424
WT-Un vs cKO-2DIV	-2.581	16.45	Yes	***	-3.291 to -1.871
cKO-Un vs WT-2DIV	-0.4802	3.063	No	ns	-1.190 to 0.2299
cKO-Un vs cKO-2DIV	-2.365	15.03	Yes	***	-3.075 to -1.655
WT-2DIV vs cKO-2DIV	-1.885	12.02	Yes	***	-2.595 to -1.175

Gad67					
Table Analyzed	Data 1				
One-way analysis of variance					
P value	< 0.0001				
P value summary	***				
Are means signif. differe	Yes				
Number of groups	4				
F	270				
R squared	0.9902				
ANOVA Table	SS	df	MS		
Treatment (between colu	22290	3	7431		
Residual (within columns	220.2	8	27.52		
Total	22510	11			
Tukey's Multiple Compari	Mean Diff.	q	Significant?	Summary	95% CI of diff
WT-Un vs cKO-Un	2.435	0.8038	No	ns	-11.28 to 16.15
WT-Un vs WT-2DIV	-77.5	25.59	Yes	***	-91.22 to -63.78
WT-Un vs cKO-2DIV	-91.32	30.15	Yes	***	-105.0 to -77.60
cKO-Un vs WT-2DIV	-79.94	26.39	Yes	***	-93.65 to -66.22
cKO-Un vs cKO-2DIV	-93.76	30.96	Yes	***	-107.5 to -80.04
WT-2DIV vs cKO-2DIV	-13.82	4.564	Yes	*	-27.54 to -0.1045

NeuN					
Table Analyzed	Data 1				
One-way analysis of variance					
P value	< 0.0001				
P value summary	***				
Are means signif. diffe	Yes				
Number of groups	4				
F	66.2				
R squared	0.9613				
ANOVA Table	SS	df	MS		
Treatment (between c	25.8	3	8.601		
Residual (within colum	1.039	8	0.1299		
Total	26.84	11			
Tukey's Multiple Comp	Mean Diff.	q	Significant?	Summary	95% CI of diff
WT-Un vs cKO-Un	-0.7715	3.707	No	ns	-1.714 to 0.1710
WT-Un vs WT-2DIV	-2.136	10.26	Yes	***	-3.079 to -1.194
WT-Un vs cKO-2DIV	-3.859	18.54	Yes	***	-4.801 to -2.916
cKO-Un vs WT-2DIV	-1.365	6.557	Yes	**	-2.307 to -0.4221
cKO-Un vs cKO-2DIV	-3.087	14.83	Yes	***	-4.030 to -2.144
WT-2DIV vs cKO-2DI	-1.722	8.276	Yes	**	-2.665 to -0.7798

Fig.4E

Dlx1 gene	Ctrl, shCon (Un)	Ctrl, shStau1 (Un)	Ctrl, shCon (2D)	Ctrl, shStau1 (2D)	Klf4OE, shCon (Un)	Klf4OE, shStau1 (Un	Klf4, shCon (2D	Klf4, shStau1 (2D)
#1	1.069759302	2.46382126	9.984688415	24.01666964	0.926711405	0.935002313	9.283651116	12.58654299
#2	0.909551886	2.665971592	14.00415594	24.37113339	0.828731809	0.672278843	8.74177274	15.31769964
#3	1.444698452	2.378631206	14.79526857	22.71571874	0.780657281	0.466696504	7.985835697	12.74585897
#4	0.951080895	1.248630691	15.77700645	20.42540833	1.126871669	0.881353625	8.565234904	12.0399668

Dlx2 gene	Ctrl, shCon (Un)	Ctrl, shStau1 (Un)	Ctrl, shCon (2D)	Ctrl, shStau1 (2D)	Klf4, shCon (Un)	Klf4, shStau1 (Un)	Klf4, shCtrl (2D)	Klf4, shStau1 (2D)
#1	0.965066036	2.512746642	3.635268872	8.527911457	0.34738677	0.661395462	3.017019473	4.935027906
#2	1.177283526	1.182015294	6.169290248	8.789939254	0.15617808	0.909788567	3.325672286	5.089262976
#3	1.065906349	0.449193517	6.825263311	11.87034979	1.437883091	2.497732889	4.42053774	6.905661495
#4	0.791102936	1.355638102	9.051185027	12.20749952	1.80678117	0.481787838	4.674583653	6.935232111

Tuj1 gene	Ctrl, shCon (Un)	Ctrl, shStau1 (Un)	Ctrl, shCon (2D)	Ctrl, shStau1 (2D)	Klf4, shCon (Un)	Klf4, shStau1 (Un)	Klf4, shCtrl (2D)	Klf4, shStau1 (2D)
#1	0.826743413	0.838455833	6.160350951	6.765273191	0.325433356	0.573939155	2.952918253	4.774892882
#2	0.763991016	0.975436533	6.273800596	8.32815402	0.359191056	0.587458203	3.205470808	3.837915309
#3	0.898354016	0.980976848	5.456800377	8.099558367	0.343887055	0.655208923	3.214136225	4.4862588
#4	1.101268213	1.162782891	4.82753263	7.916576262	0.297050723	0.755092594	3.082827631	4.679724648

Table Analyzed	Data 1		*Dlx1 gene*		
**One-way analysis of variance**					
P value	< 0.0001				
P value summary	***				
Are means signif. different? (P < 0.05)	Yes				
Number of groups	8				
F	168.4				
R squared	0.9801				
**ANOVA Table**	SS	df	MS		
Treatment (between columns)	1859	7	265.6		
Residual (within columns)	37.84	24	1.577		
Total	1897	31			
Tukey's Multiple Comparison Test	Mean Diff.	q	Significant? P < 0	Summary	95% CI of diff
Ctrl shCon (Un) vs Ctrl shStau1 (Un)	-1.095	1.745	No	ns	-4.036 to 1.845
Ctrl shCon (Un) vs Ctrl shCon (2D)	-12.55	19.98	Yes	***	-15.49 to -9.606
Ctrl shCon (Un) vs Ctrl shStau1 (2D)	-21.79	34.7	Yes	***	-24.73 to -18.85
Ctrl shCon (Un) vs Klf4OE shCon (Un)	0.178	0.2836	No	ns	-2.763 to 3.119
Ctrl shCon (Un) vs Klf4OE shStau1 (Un)	0.3549	0.5653	No	ns	-2.586 to 3.296
Ctrl shCon (Un) vs Klf4 shCon (2D)	-7.55	12.03	Yes	***	-10.49 to -4.610
Ctrl shCon (Un) vs Klf4 shStau1 (2D)	-12.08	19.24	Yes	***	-15.02 to -9.138
Ctrl shStau1 (Un) vs Ctrl shCon (2D)	-11.45	18.24	Yes	***	-14.39 to -8.510
Ctrl shStau1 (Un) vs Ctrl shStau1 (2D)	-20.69	32.96	Yes	***	-23.63 to -17.75
Ctrl shStau1 (Un) vs Klf4OE shCon (Un)	1.274	2.028	No	ns	-1.667 to 4.214
Ctrl shStau1 (Un) vs Klf4OE shStau1 (Un)	1.45	2.31	No	ns	-1.490 to 4.391
Ctrl shStau1 (Un) vs Klf4 shCon (2D)	-6.455	10.28	Yes	***	-9.396 to -3.514
Ctrl shStau1 (Un) vs Klf4 shStau1 (2D)	-10.98	17.49	Yes	***	-13.92 to -8.043
Ctrl shCon (2D) vs Ctrl shStau1 (2D)	-9.242	14.72	Yes	***	-12.18 to -6.301
Ctrl shCon (2D) vs Klf4OE shCon (Un)	12.72	20.27	Yes	***	9.784 to 15.67
Ctrl shCon (2D) vs Klf4OE shStau1 (Un)	12.9	20.55	Yes	***	9.961 to 15.84
Ctrl shCon (2D) vs Klf4 shCon (2D)	4.996	7.958	Yes	***	2.055 to 7.937
Ctrl shCon (2D) vs Klf4 shStau1 (2D)	0.4678	0.7451	No	ns	-2.473 to 3.409
Ctrl shStau1 (2D) vs Klf4OE shCon (Un)	21.97	34.99	Yes	***	19.03 to 24.91
Ctrl shStau1 (2D) vs Klf4OE shStau1 (Un)	22.14	35.27	Yes	***	19.20 to 25.08
Ctrl shStau1 (2D) vs Klf4 shCon (2D)	14.24	22.68	Yes	***	11.30 to 17.18
Ctrl shStau1 (2D) vs Klf4 shStau1 (2D)	9.71	15.47	Yes	***	6.769 to 12.65
Klf4OE shCon (Un) vs Klf4OE shStau1 (Un)	0.1769	0.2818	No	ns	-2.764 to 3.118
Klf4OE shCon (Un) vs Klf4 shCon (2D)	-7.728	12.31	Yes	***	-10.67 to -4.788
Klf4OE shCon (Un) vs Klf4 shStau1 (2D)	-12.26	19.52	Yes	***	-15.20 to -9.316
Klf4OE shStau1 (Un) vs Klf4 shCon (2D)	-7.905	12.59	Yes	***	-10.85 to -4.965
Klf4OE shStau1 (Un) vs Klf4 shStau1 (2D)	-12.43	19.8	Yes	***	-15.37 to -9.493
Klf4 shCon (2D) vs Klf4 shStau1 (2D)	-4.528	7.213	Yes	***	-7.469 to -1.588

Table Analyzed	Data 1				
**One-way analysis of variance**			*Dlx2 gene*		
P value	< 0.0001				
P value summary	***				
Are means signif. different? (P < 0.05)	Yes				
Number of groups	8				
F	29.45				
R squared	0.8957				
**ANOVA Table**	SS	df	MS		
Treatment (between columns)	333.6	7	47.66		
Residual (within columns)	38.84	24	1.618		
Total	372.5	31			
Tukey's Multiple Comparison Test	Mean Diff.	q	Significant?	Summary	95% CI of diff
Ctrl shCon (Un) vs Ctrl shStau1 (Un)	-0.3751	0.5896	No	ns	-3.354 to 2.604
Ctrl shCon (Un) vs Ctrl shCon (2D)	-5.42	8.522	Yes	***	-8.400 to -2.441
Ctrl shCon (Un) vs Ctrl shStau1 (2D)	-9.349	14.7	Yes	***	-12.33 to -6.370
Ctrl shCon (Un) vs Klf4OE shCon (Un)	0.06278	0.0987	No	ns	-2.917 to 3.042
Ctrl shCon (Un) vs Klf4OE shStau1 (Un)	-0.1378	0.2167	No	ns	-3.117 to 2.842
Ctrl shCon (Un) vs Klf4 shCon (2D)	-2.86	4.496	No	ns	-5.839 to 0.1198
Ctrl shCon (Un) vs Klf4 shStau1 (2D)	-4.966	7.808	Yes	***	-7.946 to -1.987
Ctrl shStau1 (Un) vs Ctrl shCon (2D)	-5.045	7.932	Yes	***	-8.025 to -2.066
Ctrl shStau1 (Un) vs Ctrl shStau1 (2D)	-8.974	14.11	Yes	***	-11.95 to -5.995
Ctrl shStau1 (Un) vs Klf4OE shCon (Un)	0.4378	0.6883	No	ns	-2.542 to 3.417
Ctrl shStau1 (Un) vs Klf4OE shStau1 (Un)	0.2372	0.3729	No	ns	-2.742 to 3.217
Ctrl shStau1 (Un) vs Klf4 shCon (2D)	-2.485	3.906	No	ns	-5.464 to 0.4949
Ctrl shStau1 (Un) vs Klf4 shStau1 (2D)	-4.591	7.218	Yes	***	-7.571 to -1.612
Ctrl shCon (2D) vs Ctrl shStau1 (2D)	-3.929	6.176	Yes	**	-6.908 to -0.9493
Ctrl shCon (2D) vs Klf4OE shCon (Un)	5.483	8.62	Yes	***	2.504 to 8.463
Ctrl shCon (2D) vs Klf4OE shStau1 (Un)	5.283	8.305	Yes	***	2.303 to 8.262
Ctrl shCon (2D) vs Klf4 shCon (2D)	2.561	4.026	No	ns	-0.4186 to 5.540
Ctrl shCon (2D) vs Klf4 shStau1 (2D)	0.454	0.7137	No	ns	-2.525 to 3.433
Ctrl shStau1 (2D) vs Klf4OE shCon (Un)	9.412	14.8	Yes	***	6.432 to 12.39
Ctrl shStau1 (2D) vs Klf4OE shStau1 (Un)	9.211	14.48	Yes	***	6.232 to 12.19
Ctrl shStau1 (2D) vs Klf4 shCon (2D)	6.489	10.2	Yes	***	3.510 to 9.469
Ctrl shStau1 (2D) vs Klf4 shStau1 (2D)	4.383	6.89	Yes	**	1.403 to 7.362
Klf4OE shCon (Un) vs Klf4OE shStau1 (Un)	-0.2006	0.3154	No	ns	-3.180 to 2.779
Klf4OE shCon (Un) vs Klf4 shCon (2D)	-2.922	4.594	No	ns	-5.902 to 0.05701
Klf4OE shCon (Un) vs Klf4 shStau1 (2D)	-5.029	7.907	Yes	***	-8.009 to -2.050
Klf4OE shStau1 (Un) vs Klf4 shCon (2D)	-2.722	4.279	No	ns	-5.701 to 0.2576
Klf4OE shStau1 (Un) vs Klf4 shStau1 (2D)	-4.829	7.591	Yes	***	-7.808 to -1.849
Klf4 shCon (2D) vs Klf4 shStau1 (2D)	-2.107	3.312	No	ns	-5.086 to 0.8726

Table Analyzed	Data 1				
**One-way analysis of variance**				*Tuj1 gene*	
P value	< 0.0001				
P value summary	***				
Are means signif. different? (P < 0.05)	Yes				
Number of groups	8				
F	206.8				
R squared	0.9837				
**ANOVA Table**	SS	df	MS		
Treatment (between columns)	213	7	30.42		
Residual (within columns)	3.53	24	0.1471		
Total	216.5	31			
Tukey's Multiple Comparison Test	Mean Diff.	q	Significant?	Summary	95% CI of diff
Ctrl shCon (Un) vs Ctrl shStau1 (Un)	-0.09182	0.4788	No	ns	-0.9901 to 0.8064
Ctrl shCon (Un) vs Ctrl shCon (2D)	-4.782	24.94	Yes	***	-5.680 to -3.884
Ctrl shCon (Un) vs Ctrl shStau1 (2D)	-6.88	35.88	Yes	***	-7.778 to -5.982
Ctrl shCon (Un) vs Klf4OE shCon (Un)	0.5662	2.953	No	ns	-0.3320 to 1.464
Ctrl shCon (Un) vs Klf4OE shStau1 (Un)	0.2547	1.328	No	ns	-0.6436 to 1.153
Ctrl shCon (Un) vs Klf4 shCon (2D)	-2.216	11.56	Yes	***	-3.114 to -1.318
Ctrl shCon (Un) vs Klf4 shStau1 (2D)	-3.547	18.5	Yes	***	-4.445 to -2.649
Ctrl shStau1 (Un) vs Ctrl shCon (2D)	-4.69	24.46	Yes	***	-5.588 to -3.792
Ctrl shStau1 (Un) vs Ctrl shStau1 (2D)	-6.788	35.4	Yes	***	-7.686 to -5.890
Ctrl shStau1 (Un) vs Klf4OE shCon (Un)	0.658	3.431	No	ns	-0.2402 to 1.556
Ctrl shStau1 (Un) vs Klf4OE shStau1 (Un)	0.3465	1.807	No	ns	-0.5518 to 1.245
Ctrl shStau1 (Un) vs Klf4 shCon (2D)	-2.124	11.08	Yes	***	-3.023 to -1.226
Ctrl shStau1 (Un) vs Klf4 shStau1 (2D)	-3.455	18.02	Yes	***	-4.354 to -2.557
Ctrl shCon (2D) vs Ctrl shStau1 (2D)	-2.098	10.94	Yes	***	-2.996 to -1.200
Ctrl shCon (2D) vs Klf4OE shCon (Un)	5.348	27.89	Yes	***	4.450 to 6.246
Ctrl shCon (2D) vs Klf4OE shStau1 (Un)	5.037	26.26	Yes	***	4.138 to 5.935
Ctrl shCon (2D) vs Klf4 shCon (2D)	2.566	13.38	Yes	***	1.668 to 3.464
Ctrl shCon (2D) vs Klf4 shStau1 (2D)	1.235	6.44	Yes	**	0.3367 to 2.133
Ctrl shStau1 (2D) vs Klf4OE shCon (Un)	7.446	38.83	Yes	***	6.548 to 8.344
Ctrl shStau1 (2D) vs Klf4OE shStau1 (Un)	7.134	37.2	Yes	***	6.236 to 8.033
Ctrl shStau1 (2D) vs Klf4 shCon (2D)	4.664	24.32	Yes	***	3.765 to 5.562
Ctrl shStau1 (2D) vs Klf4 shStau1 (2D)	3.333	17.38	Yes	***	2.434 to 4.231
Klf4OE shCon (Un) vs Klf4OE shStau1 (Un)	-0.3115	1.625	No	ns	-1.210 to 0.5867
Klf4OE shCon (Un) vs Klf4 shCon (2D)	-2.782	14.51	Yes	***	-3.681 to -1.884
Klf4OE shCon (Un) vs Klf4 shStau1 (2D)	-4.113	21.45	Yes	***	-5.012 to -3.215
Klf4OE shStau1 (Un) vs Klf4 shCon (2D)	-2.471	12.88	Yes	***	-3.369 to -1.573
Klf4OE shStau1 (Un) vs Klf4 shStau1 (2D)	-3.802	19.82	Yes	***	-4.700 to -2.904
Klf4 shCon (2D) vs Klf4 shStau1 (2D)	-1.331	6.94	Yes	**	-2.229 to -0.4326

Fig. 4F

Dlx1	shCtrl, Con (Un)	shCtrl, Stau1(Un)	shCtrl, Con (2D)	shCtrl, Stau1 (2D	shKlf4, co	shKlf4, Stau1 (Un)	shKlf4, Con (2D)	shKlf4, Stau1 (2D)
#1	1.069759302	2.169755875	10.53626173	5.920189314	1.741103	2.184900061	12.44316026	12.39402367
#2	0.909551886	2.144786276	10.28792915	6.177786754	2.720821	2.938738039	12.71818888	11.42056414
#3	1.444698452	1.492609724	11.24509769	6.833302565	2.106961	2.713790826	12.76090171	11.92492809
#4	0.951080895	2.160721509	10.68844589	7.165911786	2.149585	3.165500502	12.10797026	11.7530874

Dlx2 gene	shCtrl, Con (Un)	shCtrl, Stau1 (Un)	shCtrl, Con (2D)	shCtrl, Stau1 (2D)	shKlf4, Co	shKlf4, Stau1 (Un)	shKlf4, Con (2D)	shKlf4, Stau1 (2D)
#1	1.853753638	0.599323917	2.856691349	0.678412726	2.260144	2.495650739	4.171146195	2.214324422
#2	1.1317075	0.746443009	3.01586456	0.973294929	1.429833	1.822035407	4.217559396	3.474375519
#3	1.04496984	0.299661958	3.979457145	2.158648588	2.260144	0.720489853	4.248293808	4.026211793
#4	0.650994938	1.492886017	4.571195877	2.528817334	1.967569	0.469849524	5.934922233	6.948751039

Tuj1 gene	shCtrl, Con (Un)	shCtrl, Stau1 (Un)	shCtrl, Con (2D)	shCtrl, Stau1 (2D)	shKlf4, Co	shKlf4, Stau1 (Un)	shKlf4, Con (2D)	shKlf4, Stau1 (2D)
#1	0.975795752	0.912986062	4.513575155	3.976421577	2.091804	1.974751795	5.906842256	7.43022508
#2	0.847073512	0.961248113	5.051520162	4.066799141	1.686488	1.834067492	8.23459716	5.283077712
#3	1.291390299	0.74245841	5.723339338	4.342514466	1.378666	1.684127965	7.524381659	6.552093861
#4	0.953190002	0.869904095	6.325027609	4.353160052	2.195247	2.194408291	7.607513575	6.541231627

Table Analyzed	Data 1				
One-way analysis of variance				***Dlx1 gene***	
P value	< 0.0001				
P value summary	***				
Are means signif. different? (P < 0.05)	Yes				
Number of groups	8				
F	594.9				
R squared	0.9943				
ANOVA Table	SS	df	MS		
Treatment (between columns)	656.3	7	93.76		
Residual (within columns)	3.782	24	0.1576		
Total	660.1	31			
Tukey's Multiple Comparison Test	Mean Diff.	q	Significant? P < 0.(	Summary	95% CI of diff
shCtrl Con (Un) vs shCtrl Stau1(Un)	-0.8982	4.525	No	ns	-1.828 to 0.03154
shCtrl Con (Un) vs shCtrl Con (2D)	-9.596	48.34	Yes	***	-10.53 to -8.666
shCtrl Con (Un) vs shCtrl Stau1 (2D)	-5.431	27.36	Yes	***	-6.360 to -4.501
shCtrl Con (Un) vs shKlf4 con (Un)	-1.086	5.47	Yes	*	-2.016 to -0.1561
shCtrl Con (Un) vs shKlf4 Stau1 (Un)	-1.657	8.348	Yes	***	-2.587 to -0.7272
shCtrl Con (Un) vs shKlf4 Con (2D)	-11.41	57.5	Yes	***	-12.34 to -10.48
shCtrl Con (Un) vs shKlf4 Stau1 (2D)	-10.78	54.31	Yes	***	-11.71 to -9.850
shCtrl Stau1(Un) vs shCtrl Con (2D)	-8.697	43.82	Yes	***	-9.627 to -7.768
shCtrl Stau1(Un) vs shCtrl Stau1 (2D)	-4.532	22.83	Yes	***	-5.462 to -3.603
shCtrl Stau1(Un) vs shKlf4 con (Un)	-0.1876	0.9454	No	ns	-1.117 to 0.7421
shCtrl Stau1(Un) vs shKlf4 Stau1 (Un)	-0.7588	3.823	No	ns	-1.689 to 0.1710
shCtrl Stau1(Un) vs shKlf4 Con (2D)	-10.52	52.98	Yes	***	-11.45 to -9.586
shCtrl Stau1(Un) vs shKlf4 Stau1 (2D)	-9.881	49.78	Yes	***	-10.81 to -8.951
shCtrl Con (2D) vs shCtrl Stau1 (2D)	4.165	20.98	Yes	***	3.235 to 5.095
shCtrl Con (2D) vs shKlf4 con (Un)	8.51	42.87	Yes	***	7.580 to 9.440
shCtrl Con (2D) vs shKlf4 Stau1 (Un)	7.939	40	Yes	***	7.009 to 8.868
shCtrl Con (2D) vs shKlf4 Con (2D)	-1.818	9.16	Yes	***	-2.748 to -0.8884
shCtrl Con (2D) vs shKlf4 Stau1 (2D)	-1.184	5.964	Yes	**	-2.113 to -0.2540
shCtrl Stau1 (2D) vs shKlf4 con (Un)	4.345	21.89	Yes	***	3.415 to 5.274
shCtrl Stau1 (2D) vs shKlf4 Stau1 (Un)	3.774	19.01	Yes	***	2.844 to 4.703
shCtrl Stau1 (2D) vs shKlf4 Con (2D)	-5.983	30.14	Yes	***	-6.913 to -5.054
shCtrl Stau1 (2D) vs shKlf4 Stau1 (2D)	-5.349	26.95	Yes	***	-6.279 to -4.419
shKlf4 con (Un) vs shKlf4 Stau1 (Un)	-0.5711	2.877	No	ns	-1.501 to 0.3586
shKlf4 con (Un) vs shKlf4 Con (2D)	-10.33	52.03	Yes	***	-11.26 to -9.398
shKlf4 con (Un) vs shKlf4 Stau1 (2D)	-9.694	48.84	Yes	***	-10.62 to -8.764
shKlf4 Stau1 (Un) vs shKlf4 Con (2D)	-9.757	49.15	Yes	***	-10.69 to -8.827
shKlf4 Stau1 (Un) vs shKlf4 Stau1 (2D)	-9.122	45.96	Yes	***	-10.05 to -8.193
shKlf4 Con (2D) vs shKlf4 Stau1 (2D)	0.6344	3.196	No	ns	-0.2953 to 1.564

Table Analyzed	Data 1				
One-way analysis of variance				***Dlx2 gene***	
P value	< 0.0001				
P value summary	***				
Are means signif. different? (P < 0.05)	Yes				
Number of groups	8				
F	9.163				
R squared	0.7277				
ANOVA Table	SS	df	MS		
Treatment (between columns)	62.45	7	8.921		
Residual (within columns)	23.37	24	0.9736		
Total	85.81	31			
Tukey's Multiple Comparison Test	Mean Diff.	q	Significant?	Summary	95% CI of diff
shCtrl Con (Un) vs shCtrl Stau1(Un)	0.3858	0.782	No	ns	-1.925 to 2.697
shCtrl Con (Un) vs shCtrl Con (2D)	-2.435	4.937	Yes	*	-4.746 to -0.1246
shCtrl Con (Un) vs shCtrl Stau1 (2D)	-0.4144	0.8401	No	ns	-2.725 to 1.896
shCtrl Con (Un) vs shKlf4 con (Un)	-0.8091	1.64	No	ns	-3.120 to 1.502
shCtrl Con (Un) vs shKlf4 Stau1 (Un)	-0.2066	0.4189	No	ns	-2.517 to 2.104
shCtrl Con (Un) vs shKlf4 Con (2D)	-3.473	7.039	Yes	***	-5.783 to -1.162
shCtrl Con (Un) vs shKlf4 Stau1 (2D)	-2.996	6.072	Yes	**	-5.306 to -0.6847
shCtrl Stau1(Un) vs shCtrl Con (2D)	-2.821	5.719	Yes	**	-5.132 to -0.5104
shCtrl Stau1(Un) vs shCtrl Stau1 (2D)	-0.8002	1.622	No	ns	-3.111 to 1.511
shCtrl Stau1(Un) vs shKlf4 con (Un)	-1.195	2.422	No	ns	-3.506 to 1.116
shCtrl Stau1(Un) vs shKlf4 Stau1 (Un)	-0.5924	1.201	No	ns	-2.903 to 1.718
shCtrl Stau1(Un) vs shKlf4 Con (2D)	-3.858	7.821	Yes	***	-6.169 to -1.548
shCtrl Stau1(Un) vs shKlf4 Stau1 (2D)	-3.381	6.854	Yes	**	-5.692 to -1.070
shCtrl Con (2D) vs shCtrl Stau1 (2D)	2.021	4.097	No	ns	-0.2898 to 4.332
shCtrl Con (2D) vs shKlf4 con (Un)	1.626	3.297	No	ns	-0.6845 to 3.937
shCtrl Con (2D) vs shKlf4 Stau1 (Un)	2.229	4.518	No	ns	-0.08205 to 4.540
shCtrl Con (2D) vs shKlf4 Con (2D)	-1.037	2.102	No	ns	-3.348 to 1.274
shCtrl Con (2D) vs shKlf4 Stau1 (2D)	-0.5601	1.135	No	ns	-2.871 to 1.751
shCtrl Stau1 (2D) vs shKlf4 con (Un)	-0.3946	0.7999	No	ns	-2.705 to 1.916
shCtrl Stau1 (2D) vs shKlf4 Stau1 (Un)	0.2078	0.4212	No	ns	-2.103 to 2.519
shCtrl Stau1 (2D) vs shKlf4 Con (2D)	-3.058	6.199	Yes	**	-5.369 to -0.7473
shCtrl Stau1 (2D) vs shKlf4 Stau1 (2D)	-2.581	5.232	Yes	*	-4.892 to -0.2703
shKlf4 con (Un) vs shKlf4 Stau1 (Un)	0.6024	1.221	No	ns	-1.708 to 2.913
shKlf4 con (Un) vs shKlf4 Con (2D)	-2.664	5.399	Yes	*	-4.974 to -0.3527
shKlf4 con (Un) vs shKlf4 Stau1 (2D)	-2.186	4.432	No	ns	-4.497 to 0.1243
shKlf4 Stau1 (Un) vs shKlf4 Con (2D)	-3.266	6.62	Yes	**	-5.577 to -0.9551
shKlf4 Stau1 (Un) vs shKlf4 Stau1 (2D)	-2.789	5.653	Yes	*	-5.100 to -0.4781
shKlf4 Con (2D) vs shKlf4 Stau1 (2D)	0.4771	0.967	No	ns	-1.834 to 2.788

Table Analyzed	Data 1				
One-way analysis of variance				***Tuj1 gene***	
P value	< 0.0001				
P value summary	***				
Are means signif. different? (P < 0.05)	Yes				
Number of groups	8				
F	78.33				
R squared	0.9581				
ANOVA Table	SS	df	MS		
Treatment (between columns)	182.3	7	26.05		
Residual (within columns)	7.981	24	0.3325		
Total	190.3	31			
Tukey's Multiple Comparison Test	Mean Diff.	q	Significant?	Summary	95% CI of diff
shCtrl Con (Un) vs shCtrl Stau1(Un)	0.1452	0.5036	No	ns	-1.205 to 1.496
shCtrl Con (Un) vs shCtrl Con (2D)	-4.387	15.21	Yes	***	-5.737 to -3.036
shCtrl Con (Un) vs shCtrl Stau1 (2D)	-3.168	10.99	Yes	***	-4.518 to -1.817
shCtrl Con (Un) vs shKlf4 con (Un)	-0.8212	2.848	No	ns	-2.172 to 0.5293
shCtrl Con (Un) vs shKlf4 Stau1 (Un)	-0.905	3.139	No	ns	-2.256 to 0.4456
shCtrl Con (Un) vs shKlf4 Con (2D)	-6.301	21.86	Yes	***	-7.652 to -4.951
shCtrl Con (Un) vs shKlf4 Stau1 (2D)	-5.435	18.85	Yes	***	-6.785 to -4.084
shCtrl Stau1(Un) vs shCtrl Con (2D)	-4.532	15.72	Yes	***	-5.882 to -3.181
shCtrl Stau1(Un) vs shCtrl Stau1 (2D)	-3.313	11.49	Yes	***	-4.664 to -1.963
shCtrl Stau1(Un) vs shKlf4 con (Un)	-0.9664	3.352	No	ns	-2.317 to 0.3841
shCtrl Stau1(Un) vs shKlf4 Stau1 (Un)	-1.05	3.642	No	ns	-2.401 to 0.3003
shCtrl Stau1(Un) vs shKlf4 Con (2D)	-6.447	22.36	Yes	***	-7.797 to -5.096
shCtrl Stau1(Un) vs shKlf4 Stau1 (2D)	-5.58	19.35	Yes	***	-6.931 to -4.229
shCtrl Con (2D) vs shCtrl Stau1 (2D)	1.219	4.227	No	ns	-0.1319 to 2.569
shCtrl Con (2D) vs shKlf4 con (Un)	3.565	12.37	Yes	***	2.215 to 4.916
shCtrl Con (2D) vs shKlf4 Stau1 (Un)	3.482	12.07	Yes	***	2.131 to 4.832
shCtrl Con (2D) vs shKlf4 Con (2D)	-1.915	6.642	Yes	**	-3.265 to -0.5644
shCtrl Con (2D) vs shKlf4 Stau1 (2D)	-1.048	3.636	No	ns	-2.399 to 0.3022
shCtrl Stau1 (2D) vs shKlf4 con (Un)	2.347	8.139	Yes	***	0.9961 to 3.697
shCtrl Stau1 (2D) vs shKlf4 Stau1 (Un)	2.263	7.848	Yes	***	0.9124 to 3.613
shCtrl Stau1 (2D) vs shKlf4 Con (2D)	-3.134	10.87	Yes	***	-4.484 to -1.783
shCtrl Stau1 (2D) vs shKlf4 Stau1 (2D)	-2.267	7.862	Yes	***	-3.617 to -0.9164
shKlf4 con (Un) vs shKlf4 Stau1 (Un)	-0.08379	0.2906	No	ns	-1.434 to 1.267
shKlf4 con (Un) vs shKlf4 Con (2D)	-5.48	19.01	Yes	***	-6.831 to -4.130
shKlf4 con (Un) vs shKlf4 Stau1 (2D)	-4.614	16	Yes	***	-5.964 to -3.263
shKlf4 Stau1 (Un) vs shKlf4 Con (2D)	-5.396	18.72	Yes	***	-6.747 to -4.046
shKlf4 Stau1 (Un) vs shKlf4 Stau1 (2D)	-4.53	15.71	Yes	***	-5.880 to -3.179
shKlf4 Con (2D) vs shKlf4 Stau1 (2D)	0.8667	3.006	No	ns	-0.4839 to 2.217

Fig. 6B

Figure 6B — **One way Anova test (Tukey's multiple comparison test)**

Dlx1 gene — Table Analyzed: 0hr

One-way analysis of variance			
P value	0.9796		
P value summary	ns		
Are means signif. different? (P < 0.05)	No		
Number of groups	4		
F	0.05968		
R squared	0.02189		
ANOVA Table	SS	df	MS
Treatment (between columns)	0.005129	3	0.00171
Residual (within columns)	0.2292	8	0.02865
Total	0.2343	11	

	Tukey's Multiple Comparison Test	Mean Diff.	q	Significant? P < 0.05?	Summary	95% CI of diff
0hr	shCon vs S1OE	-0.002811	0.02877	No	ns	-0.4454 to 0.4398
0hr	shCon vs shKlf4	-0.04951	0.5066	No	ns	-0.4921 to 0.3931
0hr	shCon vs shKlf4/S1OE	-0.002676	0.02739	No	ns	-0.4453 to 0.4399
0hr	S1OE vs shKlf4	-0.04669	0.4778	No	ns	-0.4893 to 0.3959
0hr	S1OE vs shKlf4/S1OE	0.0001349	0.001381	No	ns	-0.4425 to 0.4427
0hr	shKlf4 vs shKlf4/S1OE	0.04683	0.4792	No	ns	-0.3958 to 0.4894

Dlx1 gene — Table Analyzed: 15min

One-way analysis of variance			
P value	< 0.0001		
P value summary	***		
Are means signif. different? (P < 0.05)	Yes		
Number of groups	4		
F	241.7		
R squared	0.9891		
ANOVA Table	SS	df	MS
Treatment (between columns)	3.711	3	1.237
Residual (within columns)	0.04095	8	0.005119
Total	3.752	11	

	Tukey's Multiple Comparison Test	Mean Diff.	q	Significant? P < 0.05?	Summary	95% CI of diff
15min	shCon vs S1OE	0.9142	22.13	Yes	***	0.7271 to 1.101
15min	shCon vs shKlf4	-0.6209	15.03	Yes	***	-0.8080 to -0.4338
15min	shCon vs shKlf4/S1OE	0.3412	8.26	Yes	**	0.1541 to 0.5283
15min	S1OE vs shKlf4	-1.535	37.16	Yes	***	-1.722 to -1.348
15min	S1OE vs shKlf4/S1OE	-0.573	13.87	Yes	***	-0.7601 to -0.3859
15min	shKlf4 vs shKlf4/S1OE	0.9621	23.29	Yes	***	0.7750 to 1.149

Dlx1 gene — Table Analyzed: 30min

One-way analysis of variance			
P value	< 0.0001		
P value summary	***		
Are means signif. different? (P < 0.05)	Yes		
Number of groups	4		
F	272		
R squared	0.9903		
ANOVA Table	SS	df	MS
Treatment (between columns)	0.7792	3	0.2597
Residual (within columns)	0.007639	8	0.0009549
Total	0.7869	11	

	Tukey's Multiple Comparison Test	Mean Diff.	q	Significant? P < 0.05?	Summary	95% CI of diff
30min	shCon vs S1OE	0.6096	34.17	Yes	***	0.5288 to 0.6904
30min	shCon vs shKlf4	-0.02529	1.417	No	ns	-0.1061 to 0.05551
30min	shCon vs shKlf4/S1OE	0.236	13.23	Yes	***	0.1552 to 0.3168
30min	S1OE vs shKlf4	-0.6349	35.59	Yes	***	-0.7157 to -0.5541
30min	S1OE vs shKlf4/S1OE	-0.3737	20.94	Yes	***	-0.4545 to -0.2929
30min	shKlf4 vs shKlf4/S1OE	0.2612	14.64	Yes	***	0.1804 to 0.3420

Dlx1 gene — Table Analyzed: 1hr

One-way analysis of variance			
P value	< 0.0001		
P value summary	***		
Are means signif. different? (P < 0.05)	Yes		
Number of groups	4		
F	39.38		
R squared	0.9366		
ANOVA Table	SS	df	MS
Treatment (between columns)	0.06922	3	0.02307
Residual (within columns)	0.004687	8	0.0005859
Total	0.07391	11	

	Tukey's Multiple Comparison Test	Mean Diff.	q	Significant? P < 0.05?	Summary	95% CI of diff
1hr	shCon vs S1OE	0.1312	9.385	Yes	***	0.06786 to 0.1944
1hr	shCon vs shKlf4	-0.05903	4.224	No	ns	-0.1223 to 0.004265
1hr	shCon vs shKlf4/S1OE	-0.05009	3.585	No	ns	-0.1134 to 0.01320
1hr	S1OE vs shKlf4	-0.1902	13.61	Yes	***	-0.2535 to -0.1269
1hr	S1OE vs shKlf4/S1OE	-0.1812	12.97	Yes	***	-0.2445 to -0.1180
1hr	shKlf4 vs shKlf4/S1OE	0.008932	0.6391	No	ns	-0.05436 to 0.07222

Dlx1 gene — Table Analyzed: 2hr

One-way analysis of variance			
P value	< 0.0001		
P value summary	***		
Are means signif. different? (P < 0.05)	Yes		
Number of groups	4		
F	59.73		
R squared	0.9573		
ANOVA Table	SS	df	MS
Treatment (between columns)	0.02662	3	0.008874
Residual (within columns)	0.001189	8	0.0001486
Total	0.02781	11	

	Tukey's Multiple Comparison Test	Mean Diff.	q	Significant? P < 0.05?	Summary	95% CI of diff
2hr	shCon vs S1OE	-0.004005	0.5692	No	ns	-0.03588 to 0.02787
2hr	shCon vs shKlf4	-0.08701	12.36	Yes	***	-0.1189 to -0.05514
2hr	shCon vs shKlf4/S1OE	-0.1038	14.75	Yes	***	-0.1357 to -0.07194
2hr	S1OE vs shKlf4	-0.083	11.79	Yes	***	-0.1149 to -0.05113
2hr	S1OE vs shKlf4/S1OE	-0.09981	14.18	Yes	***	-0.1317 to -0.06793
2hr	shKlf4 vs shKlf4/S1OE	-0.0168	2.388	No	ns	-0.04868 to 0.01507

Dlx1 gene — Table Analyzed: 4hr

One-way analysis of variance			
P value	0.0003		
P value summary	***		
Are means signif. different? (P < 0.05)	Yes		
Number of groups	4		
F	22.58		
R squared	0.8944		
ANOVA Table	SS	df	MS
Treatment (between columns)	0.0223	3	0.007435
Residual (within columns)	0.002634	8	0.0003293
Total	0.02494	11	

	Tukey's Multiple Comparison Test	Mean Diff.	q	Significant? P < 0.05?	Summary	95% CI of diff
4hr	shCon vs S1OE	0.006932	0.6617	No	ns	-0.04051 to 0.05438
4hr	shCon vs shKlf4	-0.0849	8.104	Yes	**	-0.1323 to -0.03745
4hr	shCon vs shKlf4/S1OE	-0.08022	7.657	Yes	**	-0.1277 to -0.03277
4hr	S1OE vs shKlf4	-0.09183	8.765	Yes	**	-0.1393 to -0.04438
4hr	S1OE vs shKlf4/S1OE	-0.08715	8.319	Yes	**	-0.1346 to -0.03970
4hr	shKlf4 vs shKlf4/S1OE	0.004681	0.4469	No	ns	-0.04277 to 0.05213

Dlx1 gene — Table Analyzed: 8hr

One-way analysis of variance			
P value	0.4899		
P value summary	ns		
Are means signif. different? (P < 0.05)	No		
Number of groups	4		
F	0.8827		
R squared	0.2487		
ANOVA Table	SS	df	MS
Treatment (between columns)	0.0005623	3	0.0001874
Residual (within columns)	0.001699	8	0.0002123
Total	0.002261	11	

	Tukey's Multiple Comparison Test	Mean Diff.	q	Significant? P < 0.05?	Summary	95% CI of diff
8hr	shCon vs S1OE	0.0002918	0.03468	No	ns	-0.03781 to 0.03839
8hr	shCon vs shKlf4	-0.003907	0.4644	No	ns	-0.04201 to 0.03420
8hr	shCon vs shKlf4/S1OE	-0.01654	1.966	No	ns	-0.05465 to 0.02156
8hr	S1OE vs shKlf4	-0.004199	0.4991	No	ns	-0.04230 to 0.03390
8hr	S1OE vs shKlf4/S1OE	-0.01684	2.001	No	ns	-0.05494 to 0.02127
8hr	shKlf4 vs shKlf4/S1OE	-0.01264	1.502	No	ns	-0.05074 to 0.02547

Dlx2 gene — Table Analyzed: 0hr

One-way analysis of variance			
P value	0.4137		
P value summary	ns		
Are means signif. different? (P < 0.05)	No		
Number of groups	4		
F	1.072		
R squared	0.2868		
ANOVA Table	SS	df	MS
Treatment (between columns)	0.08272	3	0.02757
Residual (within columns)	0.2057	8	0.02571
Total	0.2884	11	

	Tukey's Multiple Comparison Test	Mean Diff.	q	Significant? P < 0.05?	Summary	95% CI of diff
0hr	shCon vs S1OE	-0.2238	2.418	No	ns	-0.6431 to 0.1955
0hr	shCon vs shKlf4	-0.09173	0.9909	No	ns	-0.5110 to 0.3275
0hr	shCon vs shKlf4/S1OE	-0.05034	0.5438	No	ns	-0.4696 to 0.3689
0hr	S1OE vs shKlf4	0.1321	1.427	No	ns	-0.2872 to 0.5513
0hr	S1OE vs shKlf4/S1OE	0.1735	1.874	No	ns	-0.2458 to 0.5927
0hr	shKlf4 vs shKlf4/S1OE	0.04139	0.4471	No	ns	-0.3779 to 0.4607

Dlx2 gene — Table Analyzed: 15min

One-way analysis of variance			
P value	< 0.0001		
P value summary	***		
Are means signif. different? (P < 0.05)	Yes		
Number of groups	4		
F	203.7		
R squared	0.9871		
ANOVA Table	SS	df	MS
Treatment (between columns)	3.846	3	1.282
Residual (within columns)	0.05034	8	0.006292
Total	3.896	11	

Tukey's Multiple Comparison Test	Mean Diff.	q	Significant? P < 0.05?	Summary	95% CI of diff
shCon vs S1OE	0.9783	21.36	Yes	***	0.7709 to 1.186
shCon vs shKlf4	-0.4833	10.56	Yes	***	-0.6912 to -0.2764
shCon vs shKlf4/S1OE	0.6442	14.07	Yes	***	0.4368 to 0.8516
S1OE vs shKlf4	-1.462	31.93	Yes	***	-1.670 to -1.255
S1OE vs shKlf4/S1OE	-0.3341	7.296	Yes	**	-0.5416 to -0.1267
shKlf4 vs shKlf4/S1OE	1.123	24.63	Yes	***	0.9206 to 1.335

Dlx2 gene — Table Analyzed: 30min

One-way analysis of variance			
P value	< 0.0001		
P value summary	***		
Are means signif. different? (P < 0.05)	Yes		
Number of groups	4		
F	43.25		
R squared	0.9419		
ANOVA Table	SS	df	MS
Treatment (between columns)	0.6268	3	0.2089
Residual (within columns)	0.03864	8	0.004831
Total	0.6654	11	

Tukey's Multiple Comparison Test	Mean Diff.	q	Significant? P < 0.05?	Summary	95% CI of diff
shCon vs S1OE	0.5063	12.63	Yes	***	0.3250 to 0.6885
shCon vs shKlf4	-0.06531	1.628	No	ns	-0.2470 to 0.1164
shCon vs shKlf4/S1OE	0.2779	6.925	Yes	**	0.09614 to 0.4596
S1OE vs shKlf4	-0.5721	14.26	Yes	***	-0.7538 to -0.3903
S1OE vs shKlf4/S1OE	-0.2289	5.704	Yes	*	-0.4106 to -0.04715
shKlf4 vs shKlf4/S1OE	0.3432	8.552	Yes	**	0.1614 to 0.5249

Dlx2 gene — Table Analyzed: 1hr

One-way analysis of variance			
P value	0.0128		
P value summary	*		
Are means signif. different? (P < 0.05)	Yes		
Number of groups	4		
F	6.963		
R squared	0.7231		
ANOVA Table	SS	df	MS
Treatment (between columns)	0.01114	3	0.003712
Residual (within columns)	0.004265	8	0.0005331
Total	0.0154	11	

Tukey's Multiple Comparison Test	Mean Diff.	q	Significant? P < 0.05?	Summary	95% CI of diff
shCon vs S1OE	0.07829	5.873	Yes	*	0.01792 to 0.1387
shCon vs shKlf4	0.008037	0.6029	No	ns	-0.05234 to 0.06841
shCon vs shKlf4/S1OE	0.03033	2.279	No	ns	-0.03000 to 0.09075
S1OE vs shKlf4	-0.07026	5.27	Yes	*	-0.1306 to -0.009882
S1OE vs shKlf4/S1OE	-0.04792	3.594	No	ns	-0.1083 to 0.01246
shKlf4 vs shKlf4/S1OE	0.02231	1.676	No	ns	-0.03804 to 0.08272

Dlx2 gene — Table Analyzed: 2hr

One-way analysis of variance			
P value	0.085		
P value summary	ns		
Are means signif. different? (P < 0.05)	No		
Number of groups	4		
F	3.176		
R squared	0.5436		
ANOVA Table	SS	df	MS
Treatment (between columns)	0.002942	3	0.0009805
Residual (within columns)	0.00247	8	0.0003087
Total	0.005412	11	

Tukey's Multiple Comparison Test	Mean Diff.	q	Significant? P < 0.05?	Summary	95% CI of diff
shCon vs S1OE	0.01056	1.041	No	ns	-0.03539 to 0.05650
shCon vs shKlf4	-0.005739	0.5657	No	ns	-0.05168 to 0.04021
shCon vs shKlf4/S1OE	-0.03194	3.148	No	ns	-0.07788 to 0.01401
S1OE vs shKlf4	-0.01629	1.606	No	ns	-0.06224 to 0.02965
S1OE vs shKlf4/S1OE	-0.04249	4.189	No	ns	-0.08844 to 0.003451
shKlf4 vs shKlf4/S1OE	-0.0262	2.583	No	ns	-0.07214 to 0.01975

Dlx2 gene — Table Analyzed: 4hr

One-way analysis of variance			
P value	0.4885		
P value summary	ns		
Are means signif. different? (P < 0.05)	No		
Number of groups	4		
F	0.8859		
R squared	0.2494		
ANOVA Table	SS	df	MS
Treatment (between columns)	0.0004035	3	0.0001345
Residual (within columns)	0.001215	8	0.0001518
Total	0.001618	11	

Tukey's Multiple Comparison Test	Mean Diff.	q	Significant? P < 0.05?	Summary	95% CI of diff
shCon vs S1OE	0.01339	1.882	No	ns	-0.01883 to 0.04561
shCon vs shKlf4	0.00432	0.6072	No	ns	-0.02790 to 0.03654
shCon vs shKlf4/S1OE	-0.001497	0.2104	No	ns	-0.03372 to 0.03072
S1OE vs shKlf4	-0.009072	1.275	No	ns	-0.04129 to 0.02315
S1OE vs shKlf4/S1OE	-0.01489	2.093	No	ns	-0.04711 to 0.01733
shKlf4 vs shKlf4/S1OE	-0.005817	0.8176	No	ns	-0.03804 to 0.02640

Dlx2 gene — Table Analyzed: 8hr

One-way analysis of variance			
P value	0.1131		
P value summary	ns		
Are means signif. different? (P < 0.05)	No		
Number of groups	4		
F	2.74		
R squared	0.5068		
ANOVA Table	SS	df	MS
Treatment (between columns)	0.0006699	3	0.0002233
Residual (within columns)	0.0006519	8	0.00008149
Total	0.001322	11	

Tukey's Multiple Comparison Test	Mean Diff.	q	Significant? P < 0.05?	Summary	95% CI of diff
shCon vs S1OE	0.01793	3.45	No	ns	-0.005625 to 0.04158
shCon vs shKlf4	-3.073E-05	0.0005897	No	ns	-0.02361 to 0.02360
shCon vs shKlf4/S1OE	0.009206	1.766	No	ns	-0.01440 to 0.03281
S1OE vs shKlf4	-0.01793	3.45	No	ns	-0.04159 to 0.005622
S1OE vs shKlf4/S1OE	-0.008773	1.683	No	ns	-0.03238 to 0.01483
shKlf4 vs shKlf4/S1OE	0.009209	1.767	No	ns	-0.01439 to 0.03281

Tuj1 gene — Table Analyzed: 0hr

One-way analysis of variance			
P value	0.0127		
P value summary	*		
Are means signif. different? (P < 0.05)	Yes		
Number of groups	4		
F	6.972		
R squared	0.7233		
ANOVA Table	SS	df	MS
Treatment (between columns)	0.3784	3	0.1261
Residual (within columns)	0.1447	8	0.01809
Total	0.5232	11	

Tukey's Multiple Comparison Test	Mean Diff.	q	Significant? P < 0.05?	Summary	95% CI of diff
shCon vs S1OE	0.4395	5.66	Yes	*	0.08781 to 0.7913
shCon vs shKlf4	0.01979	0.2548	No	ns	-0.3319 to 0.3715
shCon vs shKlf4/S1OE	0.09107	1.173	No	ns	-0.2607 to 0.4428
S1OE vs shKlf4	-0.4197	5.405	Yes	*	-0.7715 to -0.06802
S1OE vs shKlf4/S1OE	-0.3485	4.487	No	ns	-0.7002 to 0.003253
shKlf4 vs shKlf4/S1OE	0.07128	0.9178	No	ns	-0.2804 to 0.4230

Tuj1 gene — Table Analyzed: 15min

One-way analysis of variance			
P value	< 0.0001		
P value summary	***		
Are means signif. different? (P < 0.05)	Yes		
Number of groups	4		
F	265.1		
R squared	0.99		
ANOVA Table	SS	df	MS
Treatment (between columns)	2.314	3	0.7715
Residual (within columns)	0.02328	8	0.00291
Total	2.338	11	

Tukey's Multiple Comparison Test	Mean Diff.	q	Significant? P < 0.05?	Summary	95% CI of diff
shCon vs S1OE	0.791	25.4	Yes	***	0.6499 to 0.9320
shCon vs shKlf4	-0.4238	13.61	Yes	***	-0.5649 to -0.2828
shCon vs shKlf4/S1OE	0.2443	7.845	Yes	**	0.1033 to 0.3854
S1OE vs shKlf4	-1.215	39	Yes	***	-1.356 to -1.074
S1OE vs shKlf4/S1OE	-0.5466	17.55	Yes	***	-0.6877 to -0.4056
shKlf4 vs shKlf4/S1OE	0.6681	21.45	Yes	***	0.5271 to 0.8092

Tuj1 gene — Table Analyzed: 30min

One-way analysis of variance			
P value	< 0.0001		
P value summary	***		
Are means signif. different? (P < 0.05)	Yes		
Number of groups	4		
F	42.01		
R squared	0.9403		
ANOVA Table	SS	df	MS
Treatment (between columns)	2.003	3	0.6677
Residual (within columns)	0.1272	8	0.01589
Total	2.13	11	

Tukey's Multiple Comparison Test	Mean Diff.	q	Significant? P < 0.05?	Summary	95% CI of diff
shCon vs S1OE	0.6603	9.072	Yes	***	0.3307 to 0.9900
shCon vs shKlf4	-0.4162	5.718	Yes	*	-0.7459 to -0.08654
shCon vs shKlf4/S1OE	0.4046	5.559	Yes	*	0.07495 to 0.7343
S1OE vs shKlf4	-1.077	14.79	Yes	***	-1.406 to -0.7469
S1OE vs shKlf4/S1OE	-0.2557	3.513	No	ns	-0.5854 to 0.07396
shKlf4 vs shKlf4/S1OE	0.8208	11.28	Yes	***	0.4911 to 1.150

Tuj1 gene — Table Analyzed: 1hr

One-way analysis of variance			
P value	0.0007		
P value summary	***		
Are means signif. different? (P < 0.05)	Yes		
Number of groups	4		
F	17.56		
R squared	0.8682		
ANOVA Table	SS	df	MS
Treatment (between columns)	0.1399	3	0.04664
Residual (within columns)	0.02124	8	0.002655
Total	0.1612	11	

Tukey's Multiple Comparison Test	Mean Diff.	q	Significant? P < 0.05?	Summary	95% CI of diff
shCon vs S1OE	0.1809	6.08	Yes	*	0.04615 to 0.3156
shCon vs shKlf4	-0.1013	3.404	No	ns	-0.2360 to 0.03347
shCon vs shKlf4/S1OE	-0.06117	2.056	No	ns	-0.1959 to 0.07358
S1OE vs shKlf4	-0.2822	9.484	Yes	***	-0.4169 to -0.1474
S1OE vs shKlf4/S1OE	-0.2421	8.136	Yes	**	-0.3768 to -0.1073
shKlf4 vs shKlf4/S1OE	0.0401	1.348	No	ns	-0.09464 to 0.1748

Tuj1 gene — Table Analyzed: 2hr

One-way analysis of variance			
P value	0.0002		
P value summary	***		
Are means signif. different? (P < 0.05)	Yes		
Number of groups	4		
F	25.73		
R squared	0.9061		
ANOVA Table	SS	df	MS
Treatment (between columns)	0.123	3	0.04099
Residual (within columns)	0.01274	8	0.001593
Total	0.1357	11	

Tukey's Multiple Comparison Test	Mean Diff.	q	Significant? P < 0.05?	Summary	95% CI of diff
shCon vs S1OE	0.1104	4.789	Yes	*	0.005991 to 0.2147
shCon vs shKlf4	-0.09253	4.017	No	ns	-0.1969 to 0.01179
shCon vs shKlf4/S1OE	-0.1588	6.89	Yes	**	-0.2631 to -0.05441
S1OE vs shKlf4	-0.2029	8.806	Yes	**	-0.3073 to -0.09857
S1OE vs shKlf4/S1OE	-0.2691	11.68	Yes	***	-0.3735 to -0.1648
shKlf4 vs shKlf4/S1OE	-0.0662	2.873	No	ns	-0.1706 to 0.03817

Tuj1 gene — Table Analyzed: 4hr

One-way analysis of variance			
P value	0.0352		
P value summary	*		
Are means signif. different? (P < 0.05)	Yes		
Number of groups	4		
F	4.721		
R squared	0.639		
ANOVA Table	SS	df	MS
Treatment (between columns)	0.021	3	0.006999
Residual (within columns)	0.01186	8	0.001483
Total	0.03286	11	

Tukey's Multiple Comparison Test	Mean Diff.	q	Significant? P < 0.05?	Summary	95% CI of diff
shCon vs S1OE	0.05713	2.57	No	ns	-0.04355 to 0.1578
shCon vs shKlf4	-0.06042	2.718	No	ns	-0.1611 to 0.04026
shCon vs shKlf4/S1OE	-0.01192	0.5361	No	ns	-0.1126 to 0.08876
S1OE vs shKlf4	-0.1176	5.288	Yes	*	-0.2182 to -0.01687
S1OE vs shKlf4/S1OE	-0.06905	3.106	No	ns	-0.1697 to 0.03163
shKlf4 vs shKlf4/S1OE	0.0485	2.182	No	ns	-0.05217 to 0.1492

Tuj1 gene — Table Analyzed: 8hr

One-way analysis of variance			
P value	0.0228		
P value summary	*		
Are means signif. different? (P < 0.05)	Yes		
Number of groups	4		
F	5.614		
R squared	0.678		
ANOVA Table	SS	df	MS
Treatment (between columns)	0.009445	3	0.003148
Residual (within columns)	0.004486	8	0.0005608
Total	0.01393	11	

Tukey's Multiple Comparison Test	Mean Diff.	q	Significant? P < 0.05?	Summary	95% CI of diff
shCon vs S1OE	-0.03624	2.651	No	ns	-0.09816 to 0.02568
shCon vs shKlf4	0.009889	0.7233	No	ns	-0.05203 to 0.07181
shCon vs shKlf4/S1OE	-0.06002	4.39	No	ns	-0.1219 to 0.001903
S1OE vs shKlf4	0.04613	3.374	No	ns	-0.01579 to 0.1081
S1OE vs shKlf4/S1OE	-0.02373	1.739	No	ns	-0.08570 to 0.03814
shKlf4 vs shKlf4/S1OE	-0.06991	5.113	Yes	*	-0.1318 to -0.007986

Fig. 6C

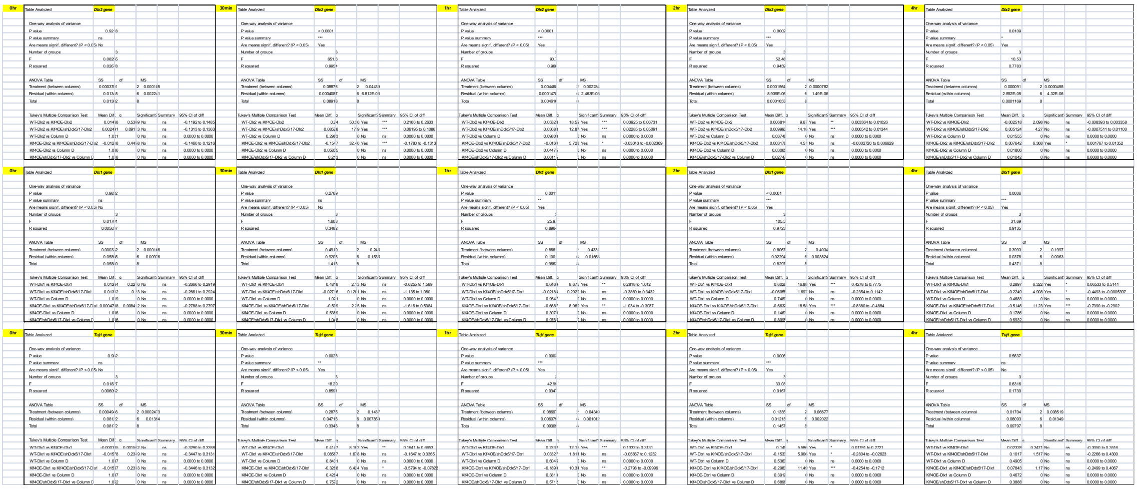

Fig. 7A

Table Analyzed	Data 1				
One-way analysis of variance					
P value	< 0.0001				
P value summary	***				
Are means signif. different? (P < 0.05)	Yes				
Number of groups	6				
F	17.49				
R squared	0.862				
ANOVA Table	SS	df	MS		
Treatment (between columns)	8065	5	1613		
Residual (within columns)	1291	14	92.21		
Total	9355	19			
**Tukey's Multiple Comparison Test**	Mean Diff.	q	Significant?	Summary	95% CI of diff
Klf4KONSC GFP-control (Un) vs Klf4KONSC GFP-mKlf4(Un)	29.87	6.222	Yes	**	7.599 to 52.15
Klf4KONSC GFP-control (Un) vs Klf4KONSC GFP-dC(Un)	12.71	2.647	No	ns	-9.566 to 34.98
Klf4KONSC GFP-control (Un) vs Klf4KONSC GFP-control (2D)	52.16	10.06	Yes	***	28.10 to 76.22
Klf4KONSC GFP-control (Un) vs Klf4KONSC GFP-mKlf4(2D)	49.66	9.576	Yes	***	25.60 to 73.72
Klf4KONSC GFP-control (Un) vs Klf4KONSC GFP-dC(2D)	48.35	8.223	Yes	***	21.07 to 75.63
Klf4KONSC GFP-mKlf4(Un) vs Klf4KONSC GFP-dC(Un)	-17.17	3.575	No	ns	-39.44 to 5.108
Klf4KONSC GFP-mKlf4(Un) vs Klf4KONSC GFP-control (2D)	22.29	4.297	No	ns	-1.771 to 46.34
Klf4KONSC GFP-mKlf4(Un) vs Klf4KONSC GFP-mKlf4(2D)	19.79	3.816	No	ns	-4.271 to 43.84
Klf4KONSC GFP-mKlf4(Un) vs Klf4KONSC GFP-dC(2D)	18.48	3.143	No	ns	-8.799 to 45.76
Klf4KONSC GFP-dC(Un) vs Klf4KONSC GFP-control (2D)	39.45	7.607	Yes	**	15.39 to 63.51
Klf4KONSC GFP-dC(Un) vs Klf4KONSC GFP-mKlf4(2D)	36.95	7.125	Yes	**	12.89 to 61.01
Klf4KONSC GFP-dC(Un) vs Klf4KONSC GFP-dC(2D)	35.65	6.062	Yes	**	8.367 to 62.92
Klf4KONSC GFP-control (2D) vs Klf4KONSC GFP-mKlf4(2D)	-2.499	0.4508	No	ns	-28.22 to 23.22
Klf4KONSC GFP-control (2D) vs Klf4KONSC GFP-dC(2D)	-3.806	0.6141	No	ns	-32.56 to 24.95
Klf4KONSC GFP-mKlf4(2D) vs Klf4KONSC GFP-dC(2D)	-1.307	0.2109	No	ns	-30.06 to 27.45

Fig.7B

Dlx1	Con-GFP (Un)	Klf4-GFP (Un)	Klf4(dC)-GFP (Un)	Con-GFP (2D)	Klf4-GFP (2D)	Klf4(dC)-GFP (2D)
#1	1.063181427	1.159388847	1.079743708	4.715546079	2.248161856	3.053965623
#2	0.97286996	1.064555289	0.985695169	4.825178117	2.93434501	3.823184385
#3	1.00672099	0.822600863	0.933405183	5.821571039	3.12124418	3.962168841
Dlx2	Con-GFP (Un)	Klf4-GFP (Un)	Klf4(dC)-GFP (Un)	Con-GFP (2D)	Klf4-GFP (2D)	Klf4(dC)-GFP (2D)
#1	1.580623248	1.23410714	1.887443338	7.676026815	1.932785564	3.944571212
#2	0.62585646	0.61705357	0.36538448	10.15292219	2.148768113	4.881520628
#3	0.916726256	1.151462677	0.877895082	7.022123894	2.571121961	6.523077157
Tuj1	Con-GFP (Un)	Klf4-GFP (Un)	Klf4(dC)-GFP (Un)	Con-GFP (2D)	Klf4-GFP (2D)	Klf4(dC)-GFP (2D)
#1	0.943198633	1.343447853	0.979410077	1.151606585	0.628564116	1.461071135
#2	1.355111055	0.687222757	1.059884505	1.298430184	0.842810928	1.285802098
#3	0.811788701	1.073481696	1.069578947	1.714672977	0.791026804	1.519104916

			0.943198633	1.343447853	0.97941	1.151607	0.628564	1.261071
0.943198633	1.355111055	0.811788701	1.355111055	0.687222757	1.059885	1.29843	0.842811	1.285802
1.343447853	0.687222757	1.073481696	0.811788701	1.073481696	1.069579	1.714673	0.791027	1.519105
0.979410077	1.059884505	1.069578947						
1.151606585	1.298430184	1.714672977						
0.628564116	0.842810928	0.791026804						
1.261071135	1.285802098	1.519104916						

	Data 1				
**One-way analysis of variance**					
P value	< 0.0001				
P value summar	***				
Are means signi	Yes				
Number of group	6				
F	61.99				
R squared	0.9627				
ANOVA Table	SS	df	MS		
Treatment (betw	44.41	5	8.883		
Residual (within	1.719	12	0.1433		
Total	46.13	17			
Tukey's Multiple	Mean Diff.	q	Significant? P <	Summary	95% CI of diff
Con-GFP (Un) v	-0.001258	0.005754	No	ns	-1.040 to 1.037
Con-GFP (Un) v	0.01464	0.067	No	ns	-1.024 to 1.053
Con-GFP (Un) v	-4.107	18.79	Yes	***	-5.145 to -3.068
Con-GFP (Un) v	-1.754	8.024	Yes	**	-2.792 to -0.7153
Con-GFP (Un) v	-2.599	11.89	Yes	***	-3.637 to -1.561
Klf4-GFP (Un) vs	0.0159	0.07275	No	ns	-1.022 to 1.054
Klf4-GFP (Un) vs	-4.105	18.78	Yes	***	-5.144 to -3.067
Klf4-GFP (Un) vs	-1.752	8.018	Yes	**	-2.791 to -0.7141
Klf4-GFP (Un) vs	-2.598	11.89	Yes	***	-3.636 to -1.559
Klf4(dC)-GFP (U	-4.121	18.86	Yes	***	-5.159 to -3.083
Klf4(dC)-GFP (U	-1.768	8.091	Yes	**	-2.807 to -0.7300
Klf4(dC)-GFP (U	-2.613	11.96	Yes	***	-3.652 to -1.575
Con-GFP (2D) v	2.353	10.77	Yes	***	1.315 to 3.391
Con-GFP (2D) v	1.508	6.899	Yes	**	0.4693 to 2.546
Klf4-GFP (2D) v	-0.8452	3.867	No	ns	-1.884 to 0.1931

Table Analyzed	Data 1				
**One-way analysis of variance**					
P value	< 0.0001				
P value summary	***				
Are means signif.	Yes				
Number of groups	6				
F	29.25				
R squared	0.9242				
ANOVA Table	SS	df	MS		
Treatment (betwee	133.8	5	26.75		
Residual (within c	10.98	12	0.9146		
Total	144.7	17			
Tukey's Multiple C	Mean Diff.	q	Significant? P <	Summary	95% CI of diff
Con-GFP (Un) vs	0.04019	0.07279	No	ns	-2.583 to 2.663
Con-GFP (Un) vs	-0.002506	0.004538	No	ns	-2.626 to 2.621
Con-GFP (Un) vs	-7.243	13.12	Yes	***	-9.866 to -4.619
Con-GFP (Un) vs	-1.176	2.131	No	ns	-3.800 to 1.447
Con-GFP (Un) vs	-4.075	7.381	Yes	**	-6.699 to -1.452
Klf4-GFP (Un) vs	-0.0427	0.07733	No	ns	-2.666 to 2.581
Klf4-GFP (Un) vs	-7.283	13.19	Yes	***	-9.906 to -4.660
Klf4-GFP (Un) vs	-1.217	2.204	No	ns	-3.840 to 1.407
Klf4-GFP (Un) vs	-4.116	7.454	Yes	**	-6.739 to -1.492
Klf4(dC)-GFP (Ur	-7.24	13.11	Yes	***	-9.863 to -4.617
Klf4(dC)-GFP (Ur	-1.174	2.126	No	ns	-3.797 to 1.449
Klf4(dC)-GFP (Ur	-4.073	7.376	Yes	**	-6.696 to -1.450
Con-GFP (2D) vs	6.066	10.99	Yes	***	3.443 to 8.689
Con-GFP (2D) vs	3.167	5.736	Yes	*	0.5440 to 5.791
Klf4-GFP (2D) vs	-2.899	5.25	Yes	*	-5.522 to -0.2755

Table Analy	Data 1				
**One-way analysis of variance**					
P value	0.0279				
P value sun	*				
Are means	Yes				
Number of	6				
F	3.762				
R squared	0.6105				
ANOVA Tal	SS	df	MS		
Treatment (	0.9535	5	0.1907		
Residual (w	0.6083	12	0.05069		
Total	1.562	17			
Tukey's Mul	Mean Diff.	q	Significant?	Summary	95% CI of diff
Con-GFP (U	0.001982	0.01525	No	ns	-0.6156 to 0.6196
Con-GFP (U	0.0004082	0.00314	No	ns	-0.6172 to 0.6180
Con-GFP (U	-0.3515	2.704	No	ns	-0.9691 to 0.2660
Con-GFP (U	0.2826	2.174	No	ns	-0.3350 to 0.9001
Con-GFP (U	-0.3853	2.964	No	ns	-1.003 to 0.2323
Klf4-GFP (U	-0.001574	0.01211	No	ns	-0.6192 to 0.6160
Klf4-GFP (U	-0.3535	2.72	No	ns	-0.9711 to 0.2641
Klf4-GFP (U	0.2806	2.159	No	ns	-0.3370 to 0.8982
Klf4-GFP (U	-0.3873	2.979	No	ns	-1.005 to 0.2303
Klf4(dC)-GF	-0.3519	2.707	No	ns	-0.9695 to 0.2656
Klf4(dC)-GF	0.2822	2.171	No	ns	-0.3354 to 0.8997
Klf4(dC)-GF	-0.3857	2.967	No	ns	-1.003 to 0.2319
Con-GFP (2	0.6341	4.878	Yes	*	0.01652 to 1.252
Con-GFP (2	-0.03376	0.2597	No	ns	-0.6513 to 0.5838
Klf4-GFP (2	-0.6679	5.138	Yes	*	-1.285 to -0.05028

Fig. S9B

Stau1	Con/shcon(Un)	Con/shStau1(Un)	Klf4/shcon(Un)	Klf4/shStau1(Un)	Con/shcon(2D)	Con/shStau1(2D)	Klf4/shcon(2D)	Klf4/shStau1(2D)
	1.00521214	0.195128985	1.111493876	0.124136562	0.251157919	0.078563336	0.228062318	0.038875364
	0.964263938	0.304075187	1.199555576	0.102237757	0.261823531	0.078020659	0.273099769	0.026923317
	0.984525173	0.165224553	1.294594249	0.111878134	0.195693354	0.083620472	0.228062318	0.044041274
	1.1076484	0.224144344	1.303598864	0.07588718	0.244289992	0.085971364	0.223368863	0.050942021
Stau2	Con/shcon(Un)	Con/shStau1(Un)	Klf4/shcon(Un)	Klf4/shStau1(Un)	Con/shcon(2D)	Con/shStau1(2D)	Klf4/shcon(2D)	Klf4/shStau1(2D)
	0.993092495	1.84889932	2.034959384	1.342572503	1.510472586	1.652900636	1.349570179	1.711190051
	0.952637998	1.967913307	2.077718207	1.664397469	1.399585866	1.596596773	1.387511761	1.618884433
	1.028113827	1.501772904	1.885569072	0.837987135	1.121166078	1.419123356	1.497441871	1.419123356
	1.049716684	1.761331747	1.796264746	1.459020344	1.176906737	1.808758755	1.349570179	1.399585866

Table Analyzed	Data 1				
		Stau1 gene			
**One-way analysis of variance**					
P value	< 0.0001				
P value summary	***				
Are means signif. different? (P < 0.05)	Yes				
Number of groups	8				
F	377.7				
R squared	0.991				
ANOVA Table	SS	df	MS		
Treatment (between columns)	5.863	7	0.8376		
Residual (within columns)	0.05323	24	0.002218		
Total	5.916	31			
Tukey's Multiple Comparison Test	Mean Diff.	q	Significant? P < 0.(	Summary	95% CI of diff
Con/shcon(Un) vs Con/shStau1(Un)	0.7933	33.69	Yes	***	0.6830 to 0.9036
Con/shcon(Un) vs Klf4/shcon(Un)	-0.2119	8.999	Yes	***	-0.3222 to -0.1016
Con/shcon(Un) vs Klf4/shStau1(Un)	0.9119	38.73	Yes	***	0.8016 to 1.022
Con/shcon(Un) vs Con/shcon(2D)	0.7772	33.01	Yes	***	0.6669 to 0.8875
Con/shcon(Un) vs Con/shStau1(2D)	0.9339	39.66	Yes	***	0.8236 to 1.044
Con/shcon(Un) vs Klf4/shcon(2D)	0.7773	33.01	Yes	***	0.6670 to 0.8876
Con/shcon(Un) vs Klf4/shStau1(2D)	0.9752	41.42	Yes	***	0.8649 to 1.086
Con/shStau1(Un) vs Klf4/shcon(Un)	-1.005	42.69	Yes	***	-1.115 to -0.8949
Con/shStau1(Un) vs Klf4/shStau1(Un)	0.1186	5.037	Yes	*	0.008317 to 0.2289
Con/shStau1(Un) vs Con/shcon(2D)	-0.0161	0.6837	No	ns	-0.1264 to 0.09419
Con/shStau1(Un) vs Con/shStau1(2D)	0.1406	5.971	Yes	**	0.03031 to 0.2509
Con/shStau1(Un) vs Klf4/shcon(2D)	-0.01601	0.6797	No	ns	-0.1263 to 0.09429
Con/shStau1(Un) vs Klf4/shStau1(2D)	0.1819	7.727	Yes	***	0.07166 to 0.2922
Klf4/shcon(Un) vs Klf4/shStau1(Un)	1.124	47.73	Yes	***	1.013 to 1.234
Klf4/shcon(Un) vs Con/shcon(2D)	0.9891	42.01	Yes	***	0.8788 to 1.099
Klf4/shcon(Un) vs Con/shStau1(2D)	1.146	48.66	Yes	***	1.035 to 1.256
Klf4/shcon(Un) vs Klf4/shcon(2D)	0.9892	42.01	Yes	***	0.8789 to 1.099
Klf4/shcon(Un) vs Klf4/shStau1(2D)	1.187	50.42	Yes	***	1.077 to 1.297
Klf4/shStau1(Un) vs Con/shcon(2D)	-0.1347	5.721	Yes	**	-0.2450 to -0.02441
Klf4/shStau1(Un) vs Con/shStau1(2D)	0.02199	0.9339	No	ns	-0.08830 to 0.1323
Klf4/shStau1(Un) vs Klf4/shcon(2D)	-0.1346	5.717	Yes	**	-0.2449 to -0.02432
Klf4/shStau1(Un) vs Klf4/shStau1(2D)	0.06334	2.69	No	ns	-0.04695 to 0.1736
Con/shcon(2D) vs Con/shStau1(2D)	0.1567	6.655	Yes	**	0.04641 to 0.2670
Con/shcon(2D) vs Klf4/shcon(2D)	0.00009288	0.003944	No	ns	-0.1102 to 0.1104
Con/shcon(2D) vs Klf4/shStau1(2D)	0.198	8.411	Yes	***	0.08775 to 0.3083
Con/shStau1(2D) vs Klf4/shcon(2D)	-0.1566	6.651	Yes	**	-0.2669 to -0.04631
Con/shStau1(2D) vs Klf4/shStau1(2D)	0.04135	1.756	No	ns	-0.06894 to 0.1516
Klf4/shcon(2D) vs Klf4/shStau1(2D)	0.198	8.407	Yes	***	0.08766 to 0.3082

Table Analyzed	Data 1				
			Stau2 gene		
**One-way analysis of variance**					
P value	< 0.0001				
P value summary	***				
Are means signif. different? (P < 0.05)	Yes				
Number of groups	8				
F	10.4				
R squared	0.7521				
ANOVA Table	SS	df	MS		
Treatment (between columns)	2.452	7	0.3503		
Residual (within columns)	0.8082	24	0.03368		
Total	3.261	31			
Tukey's Multiple Comparison Test	Mean Diff.	q	Significant?	Summary	95% CI of diff
Con/shcon(Un) vs Con/shStau1(Un)	-0.7641	8.328	Yes	***	-1.194 to -0.3343
Con/shcon(Un) vs Klf4/shcon(Un)	-0.9427	10.27	Yes	***	-1.373 to -0.5130
Con/shcon(Un) vs Klf4/shStau1(Un)	-0.3201	3.489	No	ns	-0.7499 to 0.1097
Con/shcon(Un) vs Con/shcon(2D)	-0.2961	3.228	No	ns	-0.7259 to 0.1336
Con/shcon(Un) vs Con/shStau1(2D)	-0.6135	6.686	Yes	**	-1.043 to -0.1837
Con/shcon(Un) vs Klf4/shcon(2D)	-0.3901	4.252	No	ns	-0.8199 to 0.03965
Con/shcon(Un) vs Klf4/shStau1(2D)	-0.5313	5.79	Yes	**	-0.9611 to -0.1015
Con/shStau1(Un) vs Klf4/shcon(Un)	-0.1786	1.947	No	ns	-0.6084 to 0.2511
Con/shStau1(Un) vs Klf4/shStau1(Un)	0.444	4.839	Yes	*	0.01421 to 0.8738
Con/shStau1(Un) vs Con/shcon(2D)	0.4679	5.1	Yes	*	0.03817 to 0.8977
Con/shStau1(Un) vs Con/shStau1(2D)	0.1506	1.642	No	ns	-0.2791 to 0.5804
Con/shStau1(Un) vs Klf4/shcon(2D)	0.374	4.076	No	ns	-0.05582 to 0.8037
Con/shStau1(Un) vs Klf4/shStau1(2D)	0.2328	2.537	No	ns	-0.1970 to 0.6626
Klf4/shcon(Un) vs Klf4/shStau1(Un)	0.6226	6.786	Yes	**	0.1929 to 1.052
Klf4/shcon(Un) vs Con/shcon(2D)	0.6466	7.047	Yes	***	0.2168 to 1.076
Klf4/shcon(Un) vs Con/shStau1(2D)	0.3293	3.589	No	ns	-0.1005 to 0.7591
Klf4/shcon(Un) vs Klf4/shcon(2D)	0.5526	6.023	Yes	**	0.1228 to 0.9824
Klf4/shcon(Un) vs Klf4/shStau1(2D)	0.4114	4.484	No	ns	-0.01835 to 0.8412
Klf4/shStau1(Un) vs Con/shcon(2D)	0.02396	0.2611	No	ns	-0.4058 to 0.4537
Klf4/shStau1(Un) vs Con/shStau1(2D)	-0.2934	3.197	No	ns	-0.7231 to 0.1364
Klf4/shStau1(Un) vs Klf4/shcon(2D)	-0.07003	0.7632	No	ns	-0.4998 to 0.3598
Klf4/shStau1(Un) vs Klf4/shStau1(2D)	-0.2112	2.302	No	ns	-0.6410 to 0.2186
Con/shcon(2D) vs Con/shStau1(2D)	-0.3173	3.458	No	ns	-0.7471 to 0.1125
Con/shcon(2D) vs Klf4/shcon(2D)	-0.09399	1.024	No	ns	-0.5238 to 0.3358
Con/shcon(2D) vs Klf4/shStau1(2D)	-0.2352	2.563	No	ns	-0.6649 to 0.1946
Con/shStau1(2D) vs Klf4/shcon(2D)	0.2233	2.434	No	ns	-0.2065 to 0.6531
Con/shStau1(2D) vs Klf4/shStau1(2D)	0.08215	0.8953	No	ns	-0.3476 to 0.5119
Klf4/shcon(2D) vs Klf4/shStau1(2D)	-0.1412	1.539	No	ns	-0.5710 to 0.2886

Fig. S9D

shCon&Con OE			
	mCherry&	Tuj1&mCherry&GFP	Tuj1&mCheery&GFP/mCherry&GFP
	5	2	**40**
	4	1	**25**
	7	2	**28.57143**
shStau1&Con OE			
	4	2	**50**
	7	4	**57.14286**
	5	3	**60**
shCon&Klf4 OE			
	7	1	**14.28571**
	6	1	**16.66667**
	5	1	**20**
shStau1&Klf4 OE			
	10	4	**40**
	6	3	**50**
	7	3	**42.85714**
	4	2	**50**
	5	2	**40**
	4	2	**50**

	**shCon&Con OE**	**shStau1&Con OE**	**shCon&Klf4 OE**	**shStau1&Klf4 OE**
#1	25	50	14.28571429	40
#2	28.57142857	57.14285714	16.66666667	50
#3	40	60	20	42.85714286
#4				50
#5				40
#6				50

Table Analyzed	Data 1				
**One-way analysis of variance**					
P value	< 0.0001				
P value summary	***				
Are means signif. different? (P < 0.05)	Yes				
Number of groups	4				
F	31.19				
R squared	0.8948				
ANOVA Table	SS	df	MS		
Treatment (between columns)	2727	3	908.9		
Residual (within columns)	320.6	11	29.14		
Total	3047	14			
Tukey's Multiple Comparison Test	Mean Diff.	q	Significant?	Summary	95% CI of diff
shCon&Con OE vs shStau1&Con OE	-24.52	7.868	Yes	**	-40.74 to -8.311
shCon&Con OE vs shCon&Klf4 OE	14.21	4.558	No	ns	-2.007 to 30.42
shCon&Con OE vs shStau1&Klf4 OE	-14.29	5.293	Yes	*	-28.33 to -0.2447
shStau1&Con OE vs shCon&Klf4 OE	38.73	12.43	Yes	***	22.52 to 54.94
shStau1&Con OE vs shStau1&Klf4 OE	10.24	3.793	No	ns	-3.803 to 24.28
shCon&Klf4 OE vs shStau1&Klf4 OE	-28.49	10.56	Yes	***	-42.53 to -14.45

Fig. S11B

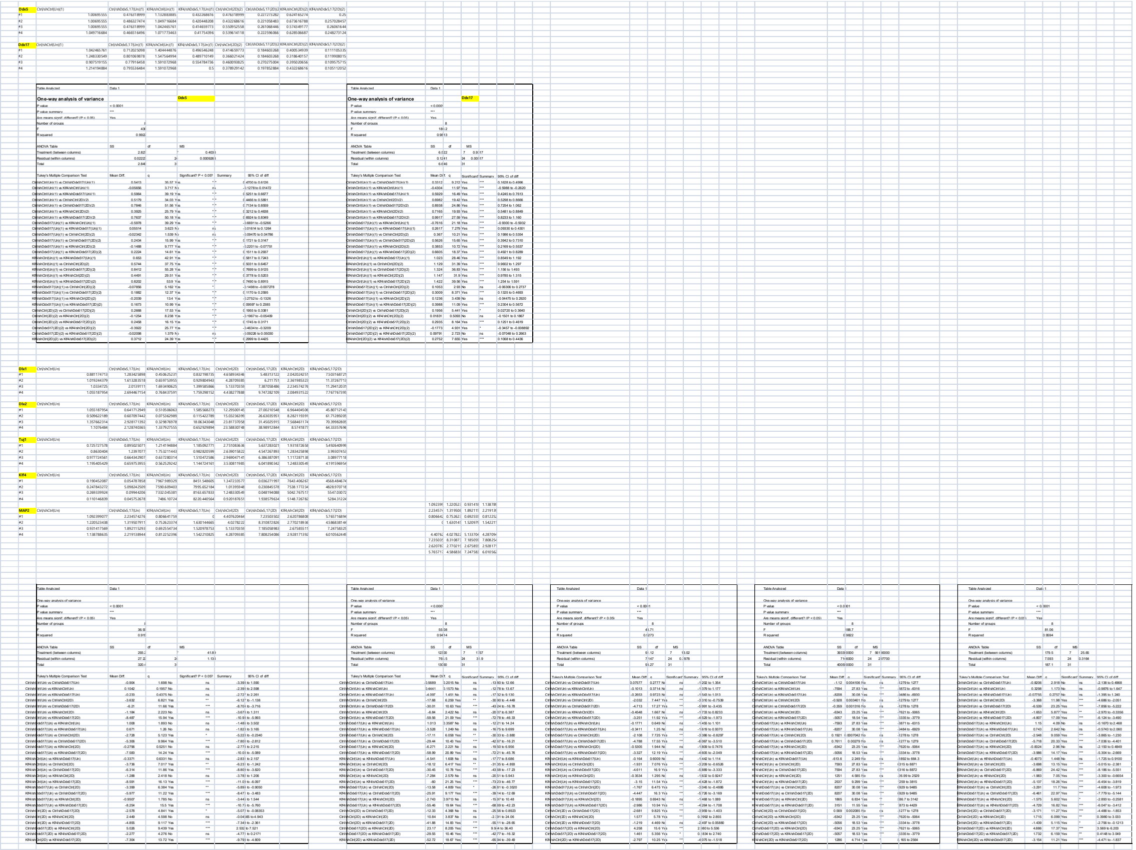

Fig. S12A

Dlx1		Ctrl/shCon	Ctrl/shDdx5,17	Stau1/shCon	Stau1/shDdx5,17
0hr	#1	1.008428528	0.965624783	0.852251748	0.932097127
	#2	1.043990683	1.020682816	1.132374808	1.012942443
	#3	1.051252213	1.027782226	0.95883306	0.998996965
	#4	0.896334317	0.985914612	1.056543054	1.055957814
Dlx1					
30min	#1	0.812032198	0.792528438	0.538437784	0.681153663
	#2	0.742060561	0.729274881	0.589209076	0.943472387
	#3	0.736934774	0.760244094	0.725401462	0.771668008
	#4	0.76822927	0.749777589	0.740643689	0.815667005
Dlx1					
1hr	#1	0.55367406	0.716746375	0.36712711	0.75578731
	#2	0.740775781	0.747183555	0.337825833	0.821340418
	#3	0.597540679	0.682799832	0.3354923	0.782440092
	#4	0.542279613	0.784331044	0.38007382	0.793362548
Dlx1					
2hr	#1	0.538533814	0.706878716	0.295627331	0.747969942
	#2	0.55367406	0.682799832	0.297683582	0.742803336
	#3	0.58931416	0.706878716	0.250321057	0.727516665
	#4	0.523807578	0.747183555	0.35646975	0.737672419
Dlx1					
4hr	#1	0.548897562	0.666434322	0.277748603	0.727516665
	#2	0.477843082	0.704433101	0.266434269	0.732576943
	#3	0.512139535	0.724237411	0.279680497	0.737672419
	#4	0.442763695	0.724237411	0.272032619	0.688272704

Dlx2		Ctrl/shCon	Ctrl/shDdx5,17	Stau1/shCon	Stau1/shDdx5,17
0hr	#1	0.917921921	1.030918903	1.157734294	1.386623644
	#2	0.735319601	1.030918903	0.927426069	0.953679404
	#3	1.381698986	1.35090933	0.993990649	0.813140336
	#4	0.963557955	0.621545041	0.927426069	0.877564011
Dlx2					
30min	#1	0.628046528	1.074697659	0.402986715	0.594222965
	#2	0.71150379	1.135974813	0.528071134	0.408689269
	#3	0.66385651	0.948638843	0.358191792	0.438022312
	#4	0.71150379	0.792196838	0.341227112	0.499679961
Dlx2					
1hr	#1	0.536423129	0.863896778	0.202192892	0.546796634
	#2	0.737872446	0.882049085	0.287932827	0.558285993
	#3	0.599338662	0.773209275	0.261304556	0.355784673
	#4	0.707814562	0.731500547	0.272401053	0.414394358
Dlx2					
2hr	#1	0.625873652	0.612988047	0.170318161	0.385305922
	#2	0.630226947	1.023797826	0.229458346	0.367057063
	#3	0.670794771	0.723934385	0.240866254	0.398893706
	#4	0.617257064	0.837366349	0.269116767	0.519097076
Dlx2					
4hr	#1	0.316755889	0.590058888	0.216704976	0.530004409
	#2	0.227106885	0.531790846	0.247209173	0.326255984
	#3	0.250250238	0.562112518	0.179717672	0.377376441
	#4	0.312395017	0.45657724	0.14597611	0.369610146

Tuj1		Ctrl/shCon	Ctrl/shDdx5,17	Stau1/shCon	Stau1/shDdx5,17
0hr	#1	0.936553418	0.994125972	0.96657503	1.008056335
	#2	0.969580903	1.015014672	1.014629935	1.134122333
	#3	1.113755988	0.980439546	0.973298091	0.841815668
	#4	0.976324871	1.015014672	1.050410782	1.008056335
Tuj1					
30min	#1	0.670325252	0.859458915	0.575725744	0.606708852
	#2	0.738634825	0.853522199	0.395967418	0.663917675
	#3	0.698791124	1.008003453	0.464404427	0.673185621
	#4	0.699760526	0.92755233	0.467634615	0.590118367
Tuj1					
1hr	#1	0.649739441	0.730269348	0.383807187	0.682582942
	#2	0.623271733	0.827310376	0.402888807	0.594222965
	#3	0.772674766	0.777276919	0.341144209	0.586042121
	#4	0.686786311	0.720215514	0.425860737	0.562169179
Tuj1					
2hr	#1	0.611503723	0.862442741	0.467634615	0.480989154
	#2	0.64190566	0.720215514	0.349521627	0.494511599
	#3	0.646370467	0.710300094	0.395967418	0.504902339
	#4	0.586593581	0.766575918	0.332967583	0.448778749
Tuj1					
4hr	#1	0.577516147	0.635736392	0.256308571	0.430497351
	#2	0.506253958	0.740463528	0.304803977	0.43650687
	#3	0.565631036	0.868441504	0.364996271	0.407275311
	#4	0.524106966	0.799129147	0.385807643	0.568044661

0hr

One-way analysis of variance

Tukey's Multiple Comparison Test	Mean Diff.	q	Significant? P < 0.05?	Summary	95% CI of diff
Ctrl/shCon vs Ctrl/shDdx517	3.576E-07	9.357E-06	No	ns	-0.1956 to 0.1956
Ctrl/shCon vs Stau1/shCon	7.153E-07	1.871E-05	No	ns	-0.1956 to 0.1956
Ctrl/shCon vs Stau1/shDdx517	2.861E-06	7.486E-05	No	ns	-0.1956 to 0.1956
Ctrl/shCon vs Column E	1	0	No	ns	0.0000 to 0.0000
Ctrl/shCon vs Column F	1	0	No	ns	0.0000 to 0.0000
Ctrl/shCon vs Column G	1	0	No	ns	0.0000 to 0.0000
Ctrl/shCon vs Column H	1	0	No	ns	0.0000 to 0.0000
Ctrl/shDdx517 vs Stau1/shCon	3.576E-07	9.357E-06	No	ns	-0.1956 to 0.1956
Ctrl/shDdx517 vs Stau1/shDdx517	2.503E-06	0.0000655	No	ns	-0.1956 to 0.1956
Ctrl/shDdx517 vs Column E	1	0	No	ns	0.0000 to 0.0000
Ctrl/shDdx517 vs Column F	1	0	No	ns	0.0000 to 0.0000
Ctrl/shDdx517 vs Column G	1	0	No	ns	0.0000 to 0.0000
Ctrl/shDdx517 vs Column H	1	0	No	ns	0.0000 to 0.0000
Stau1/shCon vs Stau1/shDdx517	2.146E-06	5.614E-05	No	ns	-0.1956 to 0.1956
Stau1/shCon vs Column E	1	0	No	ns	0.0000 to 0.0000
Stau1/shCon vs Column F	1	0	No	ns	0.0000 to 0.0000
Stau1/shCon vs Column G	1	0	No	ns	0.0000 to 0.0000
Stau1/shCon vs Column H	1	0	No	ns	0.0000 to 0.0000
Stau1/shDdx517 vs Column E	1	0	No	ns	0.0000 to 0.0000
Stau1/shDdx517 vs Column F	1	0	No	ns	0.0000 to 0.0000
Stau1/shDdx517 vs Column G	1	0	No	ns	0.0000 to 0.0000
Stau1/shDdx517 vs Column H	1	0	No	ns	0.0000 to 0.0000
Column E vs Column F	0	0	No	ns	0.0000 to 0.0000
Column E vs Column G	0	0	No	ns	0.0000 to 0.0000
Column E vs Column H	0	0	No	ns	0.0000 to 0.0000
Column F vs Column G	0	0	No	ns	0.0000 to 0.0000
Column F vs Column H	0	0	No	ns	0.0000 to 0.0000
Column G vs Column H	0	0	No	ns	0.0000 to 0.0000

30min

[illegible]

1hr

[illegible]

2hr

[illegible]

4hr

[illegible]

0hr

Tukey's Multiple Comparison Test	Mean Diff.	q	Significant	Summary	95% CI of diff
Ctrl/shCon vs Ctrl/shDdx517	-0.008948	0.07263	No	ns	-0.6396 to 0.6217
Ctrl/shCon vs Stau1/shCon	-0.00202	0.01639	No	ns	-0.6327 to 0.6286
Ctrl/shCon vs Stau1/shDdx517	-0.008127	0.06597	No	ns	-0.6388 to 0.6225
Ctrl/shCon vs Column E	0.9996	0	No	ns	0.0000 to 0.0000
Ctrl/shCon vs Column F	0.9996	0	No	ns	0.0000 to 0.0000
Ctrl/shCon vs Column G	0.9996	0	No	ns	0.0000 to 0.0000
Ctrl/shCon vs Column H	0.9996	0	No	ns	0.0000 to 0.0000
Ctrl/shDdx517 vs Stau1/shCon	0.006929	0.05624	No	ns	-0.6237 to 0.6376
Ctrl/shDdx517 vs Stau1/shDdx517	0.0008212	0.006666	No	ns	-0.6298 to 0.6315
Ctrl/shDdx517 vs Column E	1.009	0	No	ns	0.0000 to 0.0000
Ctrl/shDdx517 vs Column F	1.009	0	No	ns	0.0000 to 0.0000
[illegible]	[illegible]	[illegible]	[illegible]	[illegible]	[illegible]

30min

[illegible]

1hr

[illegible]

2hr

[illegible]

4hr

[illegible]

0hr

[illegible]

30min

[illegible]

1hr

[illegible]

2hr

[illegible]

4hr

[illegible]

Fig. S12B

Dlx1	Ctrl/shCtrl(Un)	Ctrl/shDdx5,17(Un)	Stau1/shCtrl(Un)	Stau1/shDdx5,	Ctrl/shCtrl(2D)	Ctrl/shDdx5,17(2	Stau1/shCtrl(2D)	Stau1/shDdx5,17(2D)
#1	0.768437591	1.489677463	0.864537231	1.940820463	3.095129987	3.555370725	0.926588062	3.850378
#2	0.965936329	1.370782805	0.920187651	1.609560345	3.031433133	4.055837919	1.156688184	3.149232
#3	1.613283518	1.28788163	1.404444876	1.543993487	2.4794154	4.563054863	0.993092495	3.149232
#4	1.558329159	1.859609885	0.979420298	1.861759432	3.810551992	3.580100284	1.283425898	3.193194

Dlx2	Ctrl/shCtrl(Un)	Ctrl/shDdx5,17(Un)	Stau1/shCtrl(Un)	Stau1/shDdx5,	Ctrl/shCtrl(2D)	Ctrl/shDdx5,17(2	Stau1/shCtrl(2D)	Stau1/shDdx5,17(2D)
#1	1	1.283425898	0.52123288	1.464085696	3.010493495	3.784230587	1.802500925	1.834008
#2	0.986232704	1.647182035	0.392292049	1.292352831	3.20427951	4.34693945	1.474269217	3.084422
#3	1.042465761	1.00695555	1.109569472	1.453972517	3.540615058	3.20427951	1.433955248	2.419988
#4	1.071773463	1.292352831	1.035264924	1.189207115	3.91768119	4	1.433955248	2.685145

Tuj1	Ctrl/shCtrl(Un)	Ctrl/shDdx5,17(Un)	Stau1/shCtrl(Un)	Stau1/shDdx5,	Ctrl/shCtrl(2D)	Ctrl/shDdx5,17(2	Stau1/shCtrl(2D)	Stau1/shDdx5,17(2D)
#1	0.852634892	0.680657058	0.829319546	0.930879716	1.815038311	2.694467154	1.064370182	2.305373
#2	0.801069878	0.86154616	0.716977624	0.87458267	3.530811985	2.531513188	0.624165274	1.01748
#3	0.768437591	0.942784536	0.687770909	0.833160684	3.363585661	3.555370725	0.702222438	1.563739
#4	1.414213562	1.759298152	0.939522749	1.565546833	2.203810232	2.808889751	1.231144413	3.215404

Stau1	Ctrl/shCtrl(Un)	Ctrl/shDdx5,17(Un)	Stau1/shCtrl(Un)	Stau1/shDdx5,	Ctrl/shCtrl(2D)	Ctrl/shDdx5,17(2	Stau1/shCtrl(2D)	Stau1/shDdx5,17(2D)
#1	1.866065983	5.917550037	34877.28214	59337.91716	24.25146506	4.723970646	11268.43857	28036.14
#2	3.031433133	5.010657754	48644.86836	51656.65721	24.7610399	7.621103984	13216.01858	33806.19
#3	2.989698497	3.282966435	51776.14741	63157.50971	19.8353232	3.20427951	12590.08149	35486.93
#4	2.4794154	3.237768866	32093.64431	49210.09451	21.25897303	4.59479342	12677.65243	26158.64

Table Analyzed: Data 1 — **Dlx1**

One-way analysis of variance

P value	< 0.0001
P value summary	***
Are means signif. different? (P < 0.05)	Yes
Number of groups	8
F	42.14
R squared	0.9248

ANOVA Table	SS	df	MS
Treatment (between columns)	37.21	7	5.315
Residual (within columns)	3.027	24	0.1261
Total	40.23	31	

Tukey's Multiple Comparison Test	Mean Diff.	q	Significant? P < 0.05?	Summary	95% CI of diff
Ctrl/shCtrl(Un) vs Ctrl/shDdx517(Un)	-0.2755	1.551	No	ns	-1.107 to 0.5563
Ctrl/shCtrl(Un) vs Stau1/shCtrl(Un)	0.1843	1.038	No	ns	-0.6474 to 1.016
Ctrl/shCtrl(Un) vs Stau1/shDdx517(Un)	-0.5125	2.886	No	ns	-1.344 to 0.3192
Ctrl/shCtrl(Un) vs Ctrl/shCtrl(2D)	-1.878	10.57	Yes	***	-2.709 to -1.046
Ctrl/shCtrl(Un) vs Ctrl/shDdx517(2D)	-2.712	15.27	Yes	***	-3.544 to -1.880
Ctrl/shCtrl(Un) vs Stau1/shCtrl(2D)	0.1365	0.769	No	ns	-0.6952 to 0.9683
Ctrl/shCtrl(Un) vs Stau1/shDdx517(2D)	-2.109	11.88	Yes	***	-2.941 to -1.277
Ctrl/shDdx517(Un) vs Stau1/shCtrl(Un)	0.4598	2.59	No	ns	-0.3719 to 1.292
Ctrl/shDdx517(Un) vs Stau1/shDdx517(Un)	-0.237	1.335	No	ns	-1.069 to 0.5947
Ctrl/shDdx517(Un) vs Ctrl/shCtrl(2D)	-1.602	9.022	Yes	***	-2.434 to -0.7704
Ctrl/shDdx517(Un) vs Ctrl/shDdx517(2D)	-2.437	13.72	Yes	***	-3.268 to -1.605
Ctrl/shDdx517(Un) vs Stau1/shCtrl(2D)	0.412	2.32	No	ns	-0.4197 to 1.244
Ctrl/shDdx517(Un) vs Stau1/shDdx517(2D)	-1.834	10.33	Yes	***	-2.665 to -1.002
Stau1/shCtrl(Un) vs Stau1/shDdx517(Un)	-0.6969	3.924	No	ns	-1.529 to 0.1349
Stau1/shCtrl(Un) vs Ctrl/shCtrl(2D)	-2.062	11.61	Yes	***	-2.894 to -1.230
Stau1/shCtrl(Un) vs Ctrl/shDdx517(2D)	-2.896	16.31	Yes	***	-3.728 to -2.065
Stau1/shCtrl(Un) vs Stau1/shCtrl(2D)	-0.0478	0.2692	No	ns	-0.8796 to 0.7840
Stau1/shCtrl(Un) vs Stau1/shDdx517(2D)	-2.293	12.91	Yes	***	-3.125 to -1.462
Stau1/shDdx517(Un) vs Ctrl/shCtrl(2D)	-1.365	7.687	Yes	***	-2.197 to -0.5333
Stau1/shDdx517(Un) vs Ctrl/shDdx517(2D)	-2.2	12.39	Yes	***	-3.031 to -1.368
Stau1/shDdx517(Un) vs Stau1/shCtrl(2D)	0.6491	3.655	No	ns	-0.1827 to 1.481
Stau1/shDdx517(Un) vs Stau1/shDdx517(2D)	-1.596	8.99	Yes	***	-2.428 to -0.7647
Ctrl/shCtrl(2D) vs Ctrl/shDdx517(2D)	-0.8345	4.699	Yes	*	-1.666 to -0.002696
Ctrl/shCtrl(2D) vs Stau1/shCtrl(2D)	2.014	11.34	Yes	***	1.182 to 2.846
Ctrl/shCtrl(2D) vs Stau1/shDdx517(2D)	-0.2314	1.303	No	ns	-1.063 to 0.6004
Ctrl/shDdx517(2D) vs Stau1/shCtrl(2D)	2.849	16.04	Yes	***	2.017 to 3.680
Ctrl/shDdx517(2D) vs Stau1/shDdx517(2D)	0.6031	3.396	No	ns	-0.2287 to 1.435
Stau1/shCtrl(2D) vs Stau1/shDdx517(2D)	-2.246	12.65	Yes	***	-3.077 to -1.414

Table Analyzed: Data 1 — **Dlx2**

One-way analysis of variance

P value	< 0.0001
P value summary	***
Are means signif. different? (P < 0.05)	Yes
Number of groups	8
F	46.1
R squared	0.9308

ANOVA Table	SS	df	MS
Treatment (between columns)	36.86	7	5.266
Residual (within columns)	2.742	24	0.1142
Total	39.61	31	

Tukey's Multiple Comparison Test	Mean Diff.	q	Significant?	Summary	95% CI of diff
Ctrl/shCtrl(Un) vs Ctrl/shDdx517(Un)	-0.2824	1.671	No	ns	-1.074 to 0.5092
Ctrl/shCtrl(Un) vs Stau1/shCtrl(Un)	0.2605	1.542	No	ns	-0.5310 to 1.052
Ctrl/shCtrl(Un) vs Stau1/shDdx517(Un)	-0.3248	1.922	No	ns	-1.116 to 0.4668
Ctrl/shCtrl(Un) vs Ctrl/shCtrl(2D)	-2.393	14.16	Yes	***	-3.185 to -1.602
Ctrl/shCtrl(Un) vs Ctrl/shDdx517(2D)	-2.809	16.62	Yes	***	-3.600 to -2.017
Ctrl/shCtrl(Un) vs Stau1/shCtrl(2D)	-0.5111	3.024	No	ns	-1.303 to 0.2805
Ctrl/shCtrl(Un) vs Stau1/shDdx517(2D)	-1.481	8.762	Yes	***	-2.272 to -0.6892
Ctrl/shDdx517(Un) vs Stau1/shCtrl(Un)	0.5429	3.212	No	ns	-0.2487 to 1.334
Ctrl/shDdx517(Un) vs Stau1/shDdx517(Un)	-0.04243	0.251	No	ns	-0.8340 to 0.7491
Ctrl/shDdx517(Un) vs Ctrl/shCtrl(2D)	-2.111	12.49	Yes	***	-2.902 to -1.319
Ctrl/shDdx517(Un) vs Ctrl/shDdx517(2D)	-2.526	14.95	Yes	***	-3.318 to -1.735
Ctrl/shDdx517(Un) vs Stau1/shCtrl(2D)	-0.2287	1.353	No	ns	-1.020 to 0.5629
Ctrl/shDdx517(Un) vs Stau1/shDdx517(2D)	-1.198	7.091	Yes	***	-1.990 to -0.4068
Stau1/shCtrl(Un) vs Stau1/shDdx517(Un)	-0.5853	3.464	No	ns	-1.377 to 0.2063
Stau1/shCtrl(Un) vs Ctrl/shCtrl(2D)	-2.654	15.7	Yes	***	-3.445 to -1.862
Stau1/shCtrl(Un) vs Ctrl/shDdx517(2D)	-3.069	18.16	Yes	***	-3.861 to -2.278
Stau1/shCtrl(Un) vs Stau1/shCtrl(2D)	-0.7716	4.566	No	ns	-1.563 to 0.01999
Stau1/shCtrl(Un) vs Stau1/shDdx517(2D)	-1.741	10.3	Yes	***	-2.533 to -0.9497
Stau1/shDdx517(Un) vs Ctrl/shCtrl(2D)	-2.068	12.24	Yes	***	-2.860 to -1.277
Stau1/shDdx517(Un) vs Ctrl/shDdx517(2D)	-2.484	14.7	Yes	***	-3.276 to -1.692
Stau1/shDdx517(Un) vs Stau1/shCtrl(2D)	-0.1863	1.102	No	ns	-0.9778 to 0.6053
Stau1/shDdx517(Un) vs Stau1/shDdx517(2D)	-1.156	6.84	Yes	**	-1.948 to -0.3644
Ctrl/shCtrl(2D) vs Ctrl/shDdx517(2D)	-0.4156	2.459	No	ns	-1.207 to 0.3760
Ctrl/shCtrl(2D) vs Stau1/shCtrl(2D)	1.882	11.14	Yes	***	1.091 to 2.674
Ctrl/shCtrl(2D) vs Stau1/shDdx517(2D)	0.9124	5.399	Yes	*	0.1208 to 1.704
Ctrl/shDdx517(2D) vs Stau1/shCtrl(2D)	2.298	13.6	Yes	***	1.506 to 3.089
Ctrl/shDdx517(2D) vs Stau1/shDdx517(2D)	1.328	7.858	Yes	***	0.5364 to 2.120
Stau1/shCtrl(2D) vs Stau1/shDdx517(2D)	-0.9697	5.738	Yes	**	-1.761 to -0.1781

Table Analyzed: Data 1 — **Tuj1**

One-way analysis of variance

P value	< 0.0001
P value summary	***
Are means signif. different? (P < 0.05)	Yes
Number of groups	8
F	10.14
R squared	0.7472

ANOVA Table	SS	df	MS
Treatment (between columns)	21.02	7	3.003
Residual (within columns)	7.111	24	0.2963
Total	28.13	31	

Tukey's Multiple Comparison Test	Mean Diff.	q	Significant?	Summary	95% CI of diff
Ctrl/shCtrl(Un) vs Ctrl/shDdx517(Un)	-0.102	0.3747	No	ns	-1.377 to 1.173
Ctrl/shCtrl(Un) vs Stau1/shCtrl(Un)	0.1657	0.6088	No	ns	-1.109 to 1.441
Ctrl/shCtrl(Un) vs Stau1/shDdx517(Un)	-0.09195	0.3379	No	ns	-1.367 to 1.183
Ctrl/shCtrl(Un) vs Ctrl/shCtrl(2D)	-1.769	6.501	Yes	**	-3.044 to -0.4944
Ctrl/shCtrl(Un) vs Ctrl/shDdx517(2D)	-1.938	7.122	Yes	***	-3.213 to -0.6637
Ctrl/shCtrl(Un) vs Stau1/shCtrl(2D)	0.05361	0.197	No	ns	-1.221 to 1.328
Ctrl/shCtrl(Un) vs Stau1/shDdx517(2D)	-1.066	3.918	No	ns	-2.341 to 0.2084
Ctrl/shDdx517(Un) vs Stau1/shCtrl(Un)	0.2677	0.9835	No	ns	-1.007 to 1.542
Ctrl/shDdx517(Un) vs Stau1/shDdx517(Un)	0.01003	0.03685	No	ns	-1.265 to 1.285
Ctrl/shDdx517(Un) vs Ctrl/shCtrl(2D)	-1.667	6.126	Yes	**	-2.942 to -0.3924
Ctrl/shDdx517(Un) vs Ctrl/shDdx517(2D)	-1.836	6.748	Yes	**	-3.111 to -0.5617
Ctrl/shDdx517(Un) vs Stau1/shCtrl(2D)	0.1556	0.5717	No	ns	-1.119 to 1.430
Ctrl/shDdx517(Un) vs Stau1/shDdx517(2D)	-0.9644	3.544	No	ns	-2.239 to 0.3104
Stau1/shCtrl(Un) vs Stau1/shDdx517(Un)	-0.2576	0.9466	No	ns	-1.532 to 1.017
Stau1/shCtrl(Un) vs Ctrl/shCtrl(2D)	-1.935	7.109	Yes	***	-3.210 to -0.6601
Stau1/shCtrl(Un) vs Ctrl/shDdx517(2D)	-2.104	7.731	Yes	***	-3.379 to -0.8293
Stau1/shCtrl(Un) vs Stau1/shCtrl(2D)	-0.1121	0.4118	No	ns	-1.387 to 1.163
Stau1/shCtrl(Un) vs Stau1/shDdx517(2D)	-1.232	4.527	No	ns	-2.507 to 0.04272
Stau1/shDdx517(Un) vs Ctrl/shCtrl(2D)	-1.677	6.163	Yes	**	-2.952 to -0.4024
Stau1/shDdx517(Un) vs Ctrl/shDdx517(2D)	-1.847	6.785	Yes	**	-3.121 to -0.5717
Stau1/shDdx517(Un) vs Stau1/shCtrl(2D)	0.1456	0.5348	No	ns	-1.129 to 1.420
Stau1/shDdx517(Un) vs Stau1/shDdx517(2D)	-0.9745	3.58	No	ns	-2.249 to 0.3004
Ctrl/shCtrl(2D) vs Ctrl/shDdx517(2D)	-0.1692	0.6219	No	ns	-1.444 to 1.106
Ctrl/shCtrl(2D) vs Stau1/shCtrl(2D)	1.823	6.698	Yes	**	0.5480 to 3.098
Ctrl/shCtrl(2D) vs Stau1/shDdx517(2D)	0.7028	2.582	No	ns	-0.5720 to 1.978
Ctrl/shDdx517(2D) vs Stau1/shCtrl(2D)	1.992	7.319	Yes	***	0.7173 to 3.267
Ctrl/shDdx517(2D) vs Stau1/shDdx517(2D)	0.8721	3.204	No	ns	-0.4028 to 2.147
Stau1/shCtrl(2D) vs Stau1/shDdx517(2D)	-1.12	4.115	No	ns	-2.395 to 0.1548

Table Analyzed: Data 1 — **Stau1**

One-way analysis of variance

P value	< 0.0001
P value summary	***
Are means signif. different? (P < 0.05)	Yes
Number of groups	8
F	100.2
R squared	0.9669

ANOVA Table	SS	df	MS
Treatment (between columns)	1.396E+10	7	1.995E+09
Residual (within columns)	478000000	24	19920000
Total	1.444E+10	31	

Tukey's Multiple Comparison Test	Mean Diff.	q	Significant?	Summary	95% CI of diff
Ctrl/shCtrl(Un) vs Ctrl/shDdx517(Un)	-1.771	0.0007935	No	ns	-10450 to 10450
Ctrl/shCtrl(Un) vs Stau1/shCtrl(Un)	-41850	18.75	Yes	***	-52300 to -31390
Ctrl/shCtrl(Un) vs Stau1/shDdx517(Un)	-55840	25.02	Yes	***	-66290 to -45390
Ctrl/shCtrl(Un) vs Ctrl/shCtrl(2D)	-19.94	0.008934	No	ns	-10470 to 10430
Ctrl/shCtrl(Un) vs Ctrl/shDdx517(2D)	-2.444	0.001095	No	ns	-10450 to 10450
Ctrl/shCtrl(Un) vs Stau1/shCtrl(2D)	-12440	5.573	Yes	*	-22890 to -1984
Ctrl/shCtrl(Un) vs Stau1/shDdx517(2D)	-30870	13.83	Yes	***	-41320 to -20420
Ctrl/shDdx517(Un) vs Stau1/shCtrl(Un)	-41840	18.75	Yes	***	-52300 to -31390
Ctrl/shDdx517(Un) vs Stau1/shDdx517(Un)	-55840	25.02	Yes	***	-66290 to -45380
Ctrl/shDdx517(Un) vs Ctrl/shCtrl(2D)	-18.16	0.008141	No	ns	-10470 to 10430
Ctrl/shDdx517(Un) vs Ctrl/shDdx517(2D)	-0.6738	0.000302	No	ns	-10450 to 10450
Ctrl/shDdx517(Un) vs Stau1/shCtrl(2D)	-12430	5.572	Yes	*	-22890 to -1982
Ctrl/shDdx517(Un) vs Stau1/shDdx517(2D)	-30870	13.83	Yes	***	-41320 to -20420
Stau1/shCtrl(Un) vs Stau1/shDdx517(Un)	-13990	6.271	Yes	**	-24440 to -3541
Stau1/shCtrl(Un) vs Ctrl/shCtrl(2D)	41830	18.74	Yes	***	31370 to 52280
Stau1/shCtrl(Un) vs Ctrl/shDdx517(2D)	41840	18.75	Yes	***	31390 to 52290
Stau1/shCtrl(Un) vs Stau1/shCtrl(2D)	29410	13.18	Yes	***	18960 to 39860
Stau1/shCtrl(Un) vs Stau1/shDdx517(2D)	10980	4.919	Yes	*	524.5 to 21430
Stau1/shDdx517(Un) vs Ctrl/shCtrl(2D)	55820	25.02	Yes	***	45370 to 66270
Stau1/shDdx517(Un) vs Ctrl/shDdx517(2D)	55840	25.02	Yes	***	45380 to 66290
Stau1/shDdx517(Un) vs Stau1/shCtrl(2D)	43400	19.45	Yes	***	32950 to 53850
Stau1/shDdx517(Un) vs Stau1/shDdx517(2D)	24970	11.19	Yes	***	14520 to 35420
Ctrl/shCtrl(2D) vs Ctrl/shDdx517(2D)	17.49	0.007839	No	ns	-10430 to 10470
Ctrl/shCtrl(2D) vs Stau1/shCtrl(2D)	-12420	5.564	Yes	*	-22870 to -1964
Ctrl/shCtrl(2D) vs Stau1/shDdx517(2D)	-30850	13.83	Yes	***	-41300 to -20400
Ctrl/shDdx517(2D) vs Stau1/shCtrl(2D)	-12430	5.572	Yes	*	-22880 to -1982
Ctrl/shDdx517(2D) vs Stau1/shDdx517(2D)	-30870	13.83	Yes	***	-41320 to -20420
Stau1/shCtrl(2D) vs Stau1/shDdx517(2D)	-18430	8.261	Yes	***	-28890 to -7982

Supplementary References

1. Sugimoto, Y. *et al.* hiCLIP reveals the in vivo atlas of mRNA secondary structures recognized by Staufen 1. *Nature* **519**, 491-494 (2015).
2. Ricci, E.P. *et al.* Staufen1 senses overall transcript secondary structure to regulate translation. *Nat. Struct. Mol. Biol.* **21**, 26-35 (2014).
